# *Operando* Surface Dynamics Govern the pH-Universal Activity and Stability of Cobalt Phosphide for Hydrogen Evolution

**DOI:** 10.1021/jacs.6c17411

**Published:** 2026-10-29

**Authors:** Zhongyuan Guo, Yihong Yu, Songbo Ye, Du Chen, Chaohuang Chen, Xue Jia, Di Zhang, Li Wei, Min Zhou, Song Li, Jiang Xu, Hao Li

**Affiliations:** † State Key Laboratory of Soil Pollution Control and Safety, 12377 Zhejiang University, Hangzhou 310058, China; ‡ College of Environmental and Resource Sciences, Key Laboratory of Environmental Remediation and Ecological Health, Ministry of Education, Zhejiang University, Hangzhou 310058, China; § Advanced Institute for Materials Research (WPI-AIMR), 13101 Tohoku University, Sendai 980-8577, Japan; ∥ Key Lab for Anisotropy and Texture of Materials, School of Materials Science and Engineering, 12434 Northeastern University, Shenyang 110819, China; ⊥ School of Chemical and Biomolecule Engineering, 4334 The University of Sydney, Sydney, New South Wales 2006, Australia; # Key Laboratory of Electroanalytical Chemistry, 58277 Changchun Institute of Applied Chemistry, Chinese Academy of Sciences, Changchun 130022, China; ∇ Innovation Center of Yangtze River Delta, Zhejiang University, Jiashan 314100, China

## Abstract

Cobalt phosphide (CoP) has been established as a highly promising, pH-universal electrocatalyst for the hydrogen evolution reaction (HER). However, the *operando* surface structure–activity relationship and the mechanistic origins of its high stability remain elusive. Here, we combine large-scale data mining (2,282 experimental observations), surface Pourbaix diagrams, *ab initio* molecular dynamics (AIMD), and microkinetic modeling to reveal the *operando* surface configurations of CoP. We demonstrate that at low overpotentials (<100 mV), the surface is dominated by HO*-covered configurations featuring P–OH groups. This P–OH enrichment critically modulates the electronic structure of adjacent Co active sites, optimizing H* adsorption and facilitating water activation. At high overpotentials (>100 mV), the surface undergoes a structural transition to a dense H* coverage (comprising Co–H and P–H species), which maintains highly favorable adsorption thermodynamics of H* near the Sabatier optimum (Δ*G*_H*_ = 0 eV) across the wide pH range of 0–14 under the applied electric field. Surface coverage mapping and 2D microkinetic volcano analyses, using Δ*G*_HO*_ and Δ*G*_H*_ as key descriptors, further corroborate these potential-dependent surface species and the resulting high HER activity. Crucially, these potential-dependent surface adsorbates protect CoP from detrimental element leaching. Moreover, comprehensive experimental characterizations validate our theoretical predictions. This work exemplifies the capabilities of combining AI-driven data platforms with precise simulations in accelerating scientific discoveries and uncovering underlying catalytic mechanisms.

## Graphical abstract

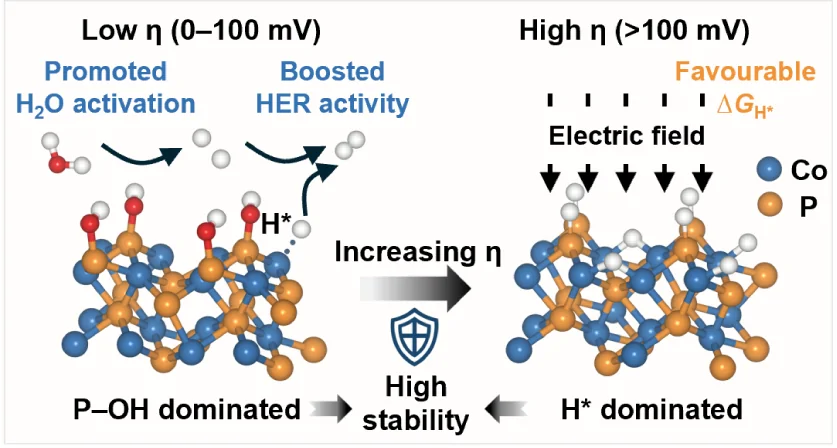

## Introduction

Hydrogen stands as a sustainable energy carrier that offers a promising, environmentally benign alternative to address the future global energy demands.^1^ Unlike conventional methane steam reforming, electrocatalytic water splitting has emerged as one of the most efficient technologies for producing carbon-neutral green hydrogen.^2,3^ Crucially, this technology enables direct integration with intermittent renewable energy sources, such as solar and wind, facilitating the conversion of sustainable energy into storable hydrogen. However, the production of hydrogen via water splitting necessitates low-cost, highly efficient, and durable electrocatalytic materials. While noble metals, such as platinum (Pt),^4^ currently remain the most active electrocatalysts for the hydrogen evolution reaction (HER), their widespread application in large-scale hydrogen production is hindered by prohibitive costs and low natural elemental abundance. Consequently, the development of earth-abundant electrocatalysts that simultaneously deliver high HER activity at low overpotentials and exceptional long-term stability is imperative for realizing a scalable, sustainable hydrogen economy.

Transition metal phosphides (TMPs) have come to the forefront as highly promising electrocatalytic candidates for electrochemical water splitting, owing to their tunable electronic structures, tailorable electrical conductivity, and excellent catalytic activities toward HER.^5^ Among transition metal phosphides, cobalt phosphide (CoP) has established as a highly promising HER electrocatalyst due to its cost-effectiveness, low overpotentials, and exceptional durability across broad pH (0–14) ranges of electrolytes.^6–8^ Furthermore, since its first report in 2014,^9^ the HER performance of CoP has been enhanced through diverse structural and electronic engineering strategies, including heteroatom doping,^10,11^ defect engineering,^12,13^ the fabrication of porous architectures,^14,15^ and the construction of interfacial heterojunctions.^16,17^ Consequently, optimized CoP-based materials now demonstrate exceptional HER performance that closely rivals benchmark noble-metal catalysts (*e.g*., Pt/C).

However, the precise *operando* structure-performance relationship remains obscure during HER over CoP: (i) according to the bulk Pourbaix diagram, CoP is thermodynamically unstable under HER conditions, resulting in its electroreduction to metallic Co (Supplementary Figure 1), which contradicts the experimental observation of high stability of as-synthesized CoP-based electrocatalysts; (ii) conventionally, electron-rich surface P sites are widely considered as the primary proton acceptor (*H) with the optimal hydrogen adsorption energy (ΔG_*H_). Nevertheless, these conventional studies typically overlook the dynamic complexities of the electrochemical environment, including electrochemistry-induced adsorbate coverage, surface reconstruction, and interfacial electric fields. Consequently, despite the extensive exploration of CoP-based electrocatalysts (Supplementary Table 1), the identity of the active surface structures under *operando* conditions remains poorly understood.

The resting surface of electrocatalysts under electrochemical conditions dictates catalytic activity and stability.^18,19^ Modulated by the applied electrode potential, the exposed catalyst surface experiences adsorbate coverage (*e.g*., O* and HO*)^20^ and, frequently, electrochemically driven reconstruction. The electrochemistry-induced generation of surface defects (*e.g*., oxygen and sulfur vacancies),^21–23^ further facilitates this structural reconstruction, consequently leading to fundamentally altered surface configurations. For example, Wang et al.^24^ found that Co_3_O_4_(111) and (110) could be fully covered by O* species under acidic oxygen evolution reaction (OER) conditions, which passivated surface Co sites but enhanced Co_3_O_4_’s structural stability. Additionally, Wang et al. and Guo et al. discovered that the exposed surfaces of Sn-based oxides could foster oxygen vacancies (V_O_) and then reconstruct into the metallic Sn under the conditions of electrochemical CO_2_ reduction, which significantly contributed to the activity toward C_1_ products.^22,23^ Therefore, the *operando* state of an electrocatalyst frequently deviates from its pristine structure. Under working conditions, the surface undergoes dynamic evolution, such as adsorbate coverage or defect formation, which fundamentally dictates both its intrinsic activity and structural stability. Furthermore, the interfacial electric field critically governs catalytic performance by modulating both the surface electronic structure and the dipole moments of reaction intermediates. Demonstrating this, our group previously elucidated the mechanistic origins of oxygen reduction reaction (ORR) activity on transition metal oxides (TMOs) and M–N–C single-atom catalysts (SACs) by employing a pH-electric-field coupling microkinetic model.^25,26^ Similarly, Chen et al. and Resasco et al. have underscored the importance of electric field effects in exploring electrochemical CO_2_ reduction.^27,28^ Therefore, it is crucial to consider electrochemistry-induced surface configurations and electric field effects when accurately unraveling the origin of the activity and stability of CoP during HER.

In this study, our comprehensive exploration includes surface coverage analysis, advanced pH-dependent simulations on the reversible hydrogen electrode (RHE) scale, the microkinetic modeling, and experimental validations. We leveraged our recently advanced Digital Catalysis Platform (*DigCat*: https://www.digcat.org/) to expedite the mechanistic elucidation of CoP as a highly active and stable HER electrocatalyst. The *DigCat* platform curates a vast repository of HER performance metrics derived from large-scale data mining, encompassing 2,282 experimental observations across ∼886 CoP-based materials extracted from ∼700 publications. Furthermore, our established theoretical frameworks, including surface Pourbaix analyses^29,30^ and advanced activity volcano models,^26,31–34^ are integrated into this AI-driven platform. This synergy empowers *DigCat* to map unexplored chemical spaces and accelerate the discovery of high-performance catalytic materials.^35^ Empowered by the well-trained large language model (LLM; namely, the *DigCat* AI Agent), the *DigCat* platform can extract and synthesize critical mechanistic insights across multiple catalytic dimensions, including highly active facets and atomistic sites, surface-covered species for stability, *operando* structure–activity relationships under applied potentials or adsorption energies of intermediates under the pH-electric-field coupling, and surface reconstructions triggered by electrochemistry-induced migration of active atoms (Figure 1A).

**Figure 1:**
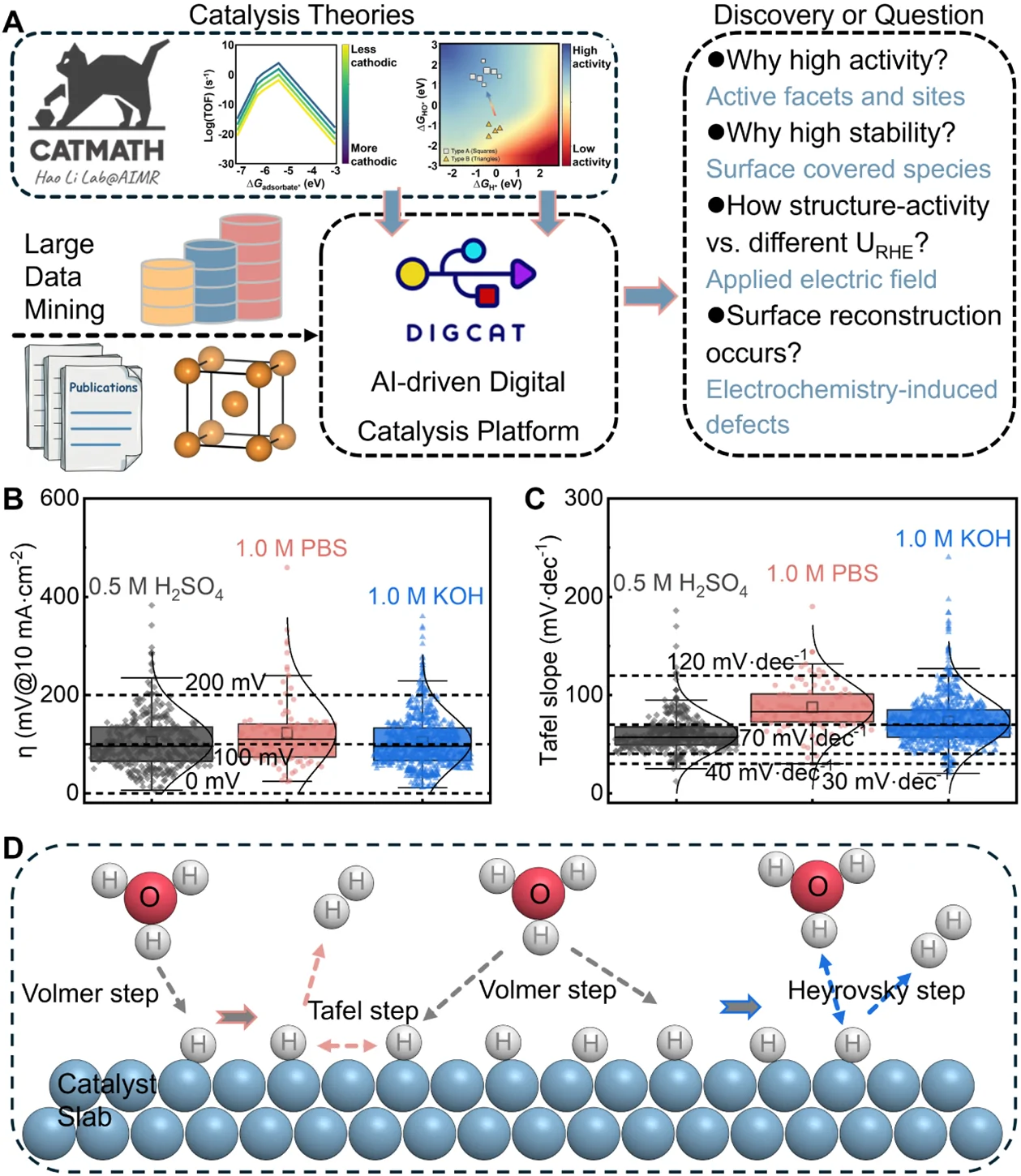
Location of questions on CoP-based HER via data mining and AI-driven digital catalysis platform (*DigCat*). A, Schematic representation of the integrated theoretical framework, which couples large-scale data mining via the AI-driven *DigCat* platform (https://www.digcat.org/) with advanced catalysis models to identify unexplored catalytic phenomena and systematically establish *operando* structure-performance relationships. B–C, Data-mined statistical distributions of HER performance metrics (overpotential and Tafel slope) for CoP-based HER electrocatalysts across a broad pH range (0–14): B, overpotentials required to reach a current density of 10 mA·cm^–2^ (η_10_) and C, corresponding Tafel slopes evaluated in typical acidic (0.5 M H_2_SO_4_), neutral (1.0 M PBS), and alkaline (1.0 M KOH) electrolytes. The average values of η_10_ and Tafel slope are 100 mV and 70 mV·dec^–1^ across different electrolytes, respectively. The corresponding rate-determining steps, assigned to the Volmer, Tafel, and Heyrovsky processes with characteristic Tafel slopes of 120, 30, and 40 mV·dec^–1^ respectively, are also explicitly presented. Detailed extracted data sets and the corresponding references are provided in Supplementary Table 1. D, HER mechanisms, including the Volmer–Tafel and Volmer-Heyrovsky pathways.

Interestingly, it is found that the electrochemistry-induced HO* species occupy the surface P active sites of CoP at a wide electrolyte pH range under the low overpotential (<100 mV), where the current densities reach to ∼10 mA·cm^–2^. The surface-formed HO* species can not only promote water adsorption by hydrogen bonding and modulate the adsorbed H* energy with surface Co as main active sites but also protect the surface from being dissolved to form phosphates. Under the high overpotential (>100 mV), the electrochemistry-induced H* species cover the exposed CoP surfaces with high coverages, where surface H* species still demonstrate favorable Gibbs adsorption energies with both Co and P as active sites at various electric fields. Surface state analyses suggest that CoP surfaces possess high stability at *operando* HER conditions except extremely high external electric fields are employed to create P defects on them. Furthermore, taking the coupling of HO* and H* adsorption into consideration, our developed theoretical models confirm the electrochemistry-indued surface coverage species and the HER activity close to the theoretical activity-volcano peak under different overpotentials. Finally, our experimental results validate the high HER performance and surface key species accountable for high activity and stability of CoP-based materials. Under the framework of the 4th/4^th+^ research paradigm,^36,37^ our research not only provides crucial insights into the surface-active structures and the corresponding origins of high activity and stability for CoP but also accelerates the design of high-performance electrocatalysts at the complex interfacial microenvironment.

## Results and Discussion

### Data Mining and Database-Driven Analysis on HER Mechanisms

Figure 1A demonstrates how the AI-driven *DigCat* platform aids in accelerating the identification of critical knowledge gaps and high-performance catalyst discovery, fundamentally underpinned by large-scale data mining and established catalysis theories. The *DigCat* platform can extract mechanistic insights regarding catalytic activity, stability, and electrochemical behaviors under operational conditions. Furthermore, by integrating surface state evaluations (CATMATH) with advanced pH-electric-field-coupled activity volcano models, *DigCat* predictively designs optimal catalyst architectures. Therefore, these capabilities provided the strategic roadmap for this study, directing our focus toward resolving the underlying stability and *operando* structure–activity relationships of CoP during the HER. Figure 1B–C and Supplementary Figures 2–3 present the comprehensive data mining results encompassing 2,282 experimental observations across ∼886 CoP-based HER electrocatalysts extracted from ∼700 publications. The analysis evaluates the overpotentials at the standard current density of 10 mA·cm^–2^ (η_10_) and the corresponding Tafel slopes across acidic (0.5 M H_2_SO_4_), neutral (1.0 M PBS), and alkaline (1.0 M KOH) electrolytes. According to the statistics of overpotentials and Tafel slopes, it is found that CoP-based materials have similar distributions for η_10_ and Tafel slopes at different electrolytes, indicating they may share analogous surface-active configurations at the wide pH range. To be specific, the η_10_ of the majority of reported CoP-based materials ranges from 0 to 200 mV with the average value of 100 mV, and the Tafel slope typically locates between 40 and 120 mV·dec^–1^ with the average value of 70 mV·dec^–1^. Furthermore, across different electrolytes, both η_10_ and Tafel slope performance metrics demonstrate highly comparable statistical distributions with close medians and averages. The fundamental HER mechanistic pathways are illustrated in Figure 1D. The process initiates with the Volmer step, corresponding to the initial adsorption of a proton onto an active site (* + H^+^ + e^–^ → H*, where * represents the active site). Subsequent H_2_ evolution proceeds either via the Tafel step, through the recombination of two adjacent adsorbed intermediates (H* + H* → * + * + H_2_), or via the Heyrovsky step, where an adsorbed H* reacts with a solvated proton from the electrolyte (H* + H^+^ + e^–^ → * + H_2_). According to the HER kinetic models, the rate-determining steps of Volmer, Tafel, and Heyrovsky processes have the Tafel slopes of 120, 30, and 40 mV·dec^–1^, respectively.^38,39^ Therefore, our statistical findings, particularly the average slope of ∼70 mV·dec^–1^, not only underscore the inherently fast kinetics and low overpotentials for most CoP-based electrocatalysts, but also indicate that a Volmer-Heyrovsky pathway predominantly governs their electrocatalytic process.

### Surface Pourbaix and *Ab Initio* Molecular Dynamics (AIMD) of Exposed CoP Surfaces

The surface configurations of CoP materials during HER are responsible for the delivered η_10_ and Volmer-Heyrovsky mechanism. Therefore, we systematically evaluated the surface energies (γ, J·m^–2^) of its thermodynamically accessible facets. The computed bulk CoP lattice parameters agree well with previously reported values (Supplementary Table 2). To systematically identify the most thermodynamically stable facets, seven low-index surfaces, specifically, the CoP(011), (111), (001), (010), (102), (201), and (100) planes, were considered (Supplementary Figure 4) and their γ values were evaluated (Supplementary Figure 5 and Supplementary Table 3) with CoP(011) as the most stable surface, in accordance with the previous report.^6^ The surface energy of CoP surfaces follows the order of CoP(011) < CoP(111) < CoP(001) < CoP(010) < CoP(102) < CoP(201) < CoP(100). Generally, crystal facets with lower surface energies are thermodynamically more stable and thus preferentially exposed. Therefore, these four facets of CoP(011), (111), (001), and (010) are selected as the representative crystal planes of experimentally synthesized CoP materials. However, the complex electrochemical interface can fundamentally alter these pristine configurations, driving the surface to restructure into a dynamic *operando* resting state governed by coupled pH and electric field effects.^18,19,30^ Therefore, the surface coverages of four CoP planes have been analyzed by the surface Pourbaix diagrams, where H*, O*. HO*, P_V_* (phosphorus vacancy), and H_2_O* species are taken into account (Figure 2A–D and Supplementary Figures 6–9). Based on the *operando* η_10_ of most CoP-based electrocatalysts spanning from 0 to 200 mV, it is noticed that the surface covering species of exposed CoP surfaces evolves from HO* to H* species (Figure 2A–D). At the low η_10_ window of ranging from 0 to 100 mV, only surface P active sites of CoP are selectively occupied by HO* species with the formation of P–OH groups, effectively passivating them and leaving only the surface Co sites exposed (Figure 2E). At the high η_10_ window of ranging from 100 to 200 mV, the surface P and Co active sites of CoP are occupied by H* species with the formation of P–H and Co–H bonds, consequently forming a high coverage of H* for CoP electrocatalysts operated in high overpotential (Figure 2F). AIMD simulations, conducted over a 100 ps time scale to investigate the surface dynamics of the electrochemically induced configurations (CoP–HO* and CoP–H*), indicate that the resting surface states of CoP remain stable without obvious reconstruction during the HER (Supplementary Figures 10–13). Moreover, AIMD simulations indicate that the HO*/H*-covered CoP can suppress the direct H_2_O adsorption on surface active sites, avoiding surface oxidation toward element leaching during HER (Supplementary Figure 14). Hence, experimentally reported CoP-based electrocatalysts have distinct resting surface configurations at the η_10_ window, which can influence the HER activity differently. Meanwhile, based on AIMD results, the electrochemistry-induced covering species of HO* and H* cannot cause surface reconstruction, and the surface HO* and H*-covered species protect CoP from being oxidized by H_2_O into possible element leaching, accordingly promising the high stability of CoP-based electrocatalysts, which will be further discussed in experimental HER tests.

**Figure 2:**
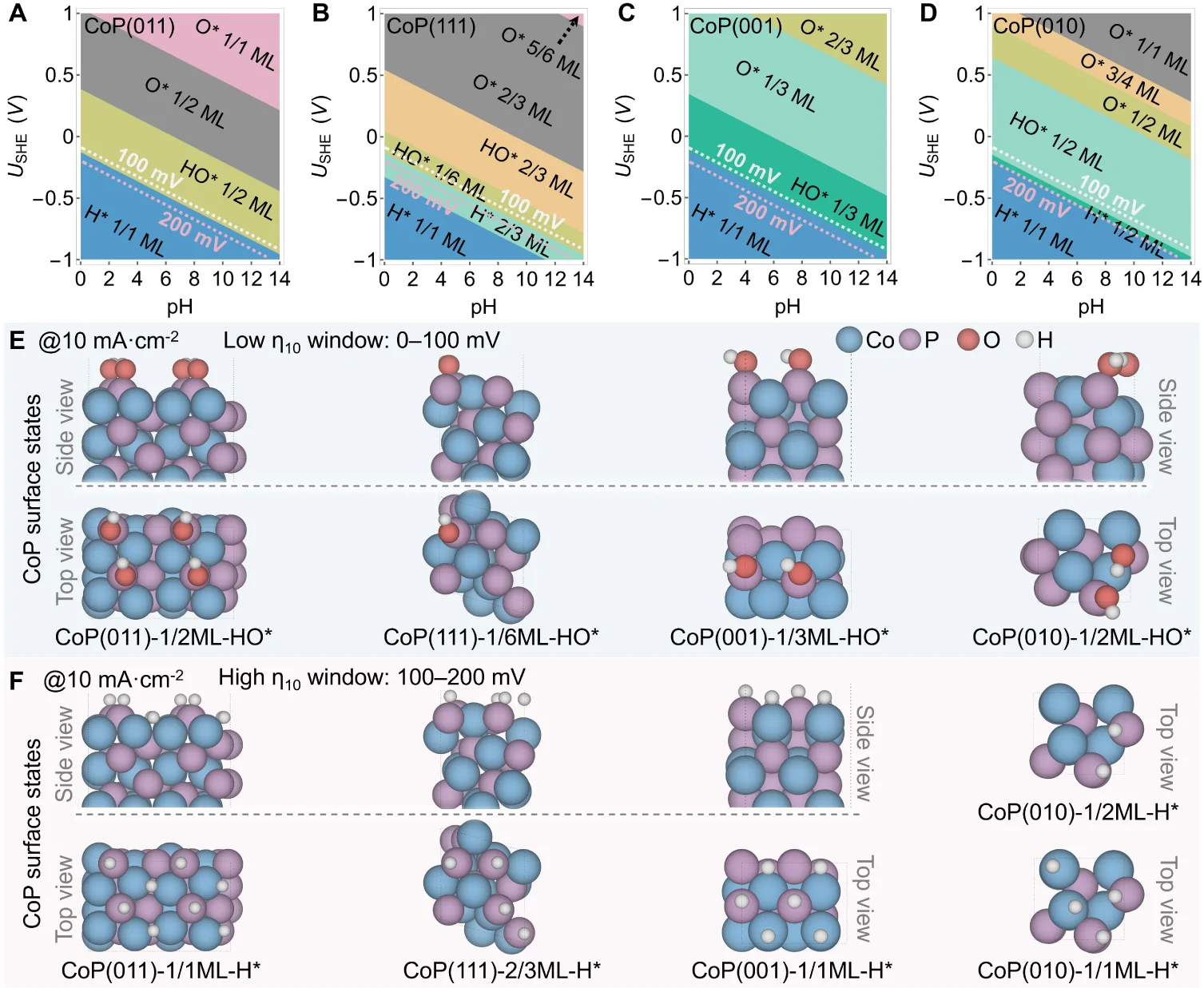
Surface state analysis of CoP under *in situ* conditions. Surface Pourbaix diagrams of exposed CoP surfaces, including (A) CoP(011), (B) CoP(111), (C) CoP(001), and (D) CoP(010). The 100 mV and 200 mV lines demarcate the investigated overpotential regimes. The 100 mV line reflects the statistical average η_10_ of CoP-based materials and illustrates the thermodynamic transition from an HO*-dominated resting state at the low overpotential window (0–100 mV) to an H*-dominated state at the high overpotential window (100–200 mV) under *operando* HER conditions. E, Surface configurations of various CoP facets with the HO* coverage within a low η_10_ window of 0–100 mV. F, Surface configurations of various CoP facets with the H* coverage within a high η_10_ window of 100–200 mV.

### Effects of Surface Adsorbed Hydroxyl on HER under Low Overpotentials

The emergence of HO*-covered surface configurations profoundly alters the structure–activity relationship for the HER in the low-overpotential regime (0 < η_10_ < 100 mV). Figure 3A illustrates the structural evolution of the CoP electrochemical interface from a pristine state to an HO*-terminated surface within this potential window. Following this electrochemically driven surface evolution, nearly all surface P sites become hydroxylated to form P–OH groups, thereby selectively leaving the Co sites exposed as the primary active centers. For instance, on the thermodynamically most stable CoP(011) facet, HO* species saturate all surface P sites, resulting in a net coverage of 0.5 ML (denoted as CoP(011)-1/2 ML-HO*). Our comprehensive data mining reveals that most reported CoP-based electrocatalysts proceed via the Volmer–Heyrovsky mechanism, exhibiting an average Tafel slope of ∼70 mV·dec^–1^. Consequently, we systematically evaluated the adsorption behavior of active H* on both the pristine (Figure 3B–E) and the HO*-covered CoP surfaces (Figure 3F–I). It is found that surface-adsorbed HO* species intrinsically modulate HER activity by altering both the preferred binding sites and the local electronic structure for active H* intermediates, as discussed below. On pristine CoP surfaces (Figure 3B–E), both P and Co sites can adsorb H*; however, the P sites generally exhibit more favorable hydrogen adsorption free energies (ΔG_H*_), with the P site on CoP(011) approaching near-thermoneutrality (Figure 3B, ΔG_H*_ = 0.04 eV). In contrast, the presence of surface HO* fundamentally shifts both the primary active site and the associated binding energetics (Figure 3F–I). On these HO*-covered surfaces, the exposed Co atoms emerge as the dominant H* adsorption sites. Notably, Co atoms act as the exclusive H* binding centers on the CoP(011)-1/2 ML-HO*, CoP(001)-1/3 ML-HO*, and CoP(010)-1/2 ML-HO*. Moreover, the surface-adsorbed HO* species intrinsically enhance H* binding at these Co sites, ultimately yielding a highly favorable ΔG_H*_ for the HER. For instance, upon establishing a 1/2 ML HO* coverage on the CoP(011) facet, the ΔG_H*_ at the exposed Co sites shifts favorably from 0.28 to 0.12 eV. To elucidate the mechanistic origins of the HO*-induced activity enhancement at Co sites, we analyzed the electronic structures of exposed Co on both pristine and HO*-covered CoP surfaces (Supplementary Figure 15). The Co *d*-band density of states (DOS) reveals that surface-adsorbed HO* induces a slight downshift in the *d*-band center, which typically attenuates H* binding (Supplementary Figure 15A–D). However, Bader charge analysis indicates that these HO* adsorbates concurrently decrease the partial positive charge on the Co active sites (with the exception of CoP(001)), thereby strengthening H* adsorption (Supplementary Figure 15E–H). On the CoP(001) facet, HO* coverage instead changes the surface Co–P bonding, creating a distinct local coordination environment that enhances H* affinity (Figure 3H, ΔG_H*_ from 0.40 to 0.19 and 0.25 eV after the adsorption of HO*). Beyond modulating H* energetics, surface HO* species significantly promote H_2_O adsorption via hydrogen bonding, a critical prerequisite for water activation (Figure 3J–M). Furthermore, the low H_2_O dissociation barriers to yield H* on these HO*-covered surfaces indicate highly favorable reaction kinetics for proton supply, even in neutral or alkaline electrolytes (Supplementary Figure 16).

**Figure 3:**
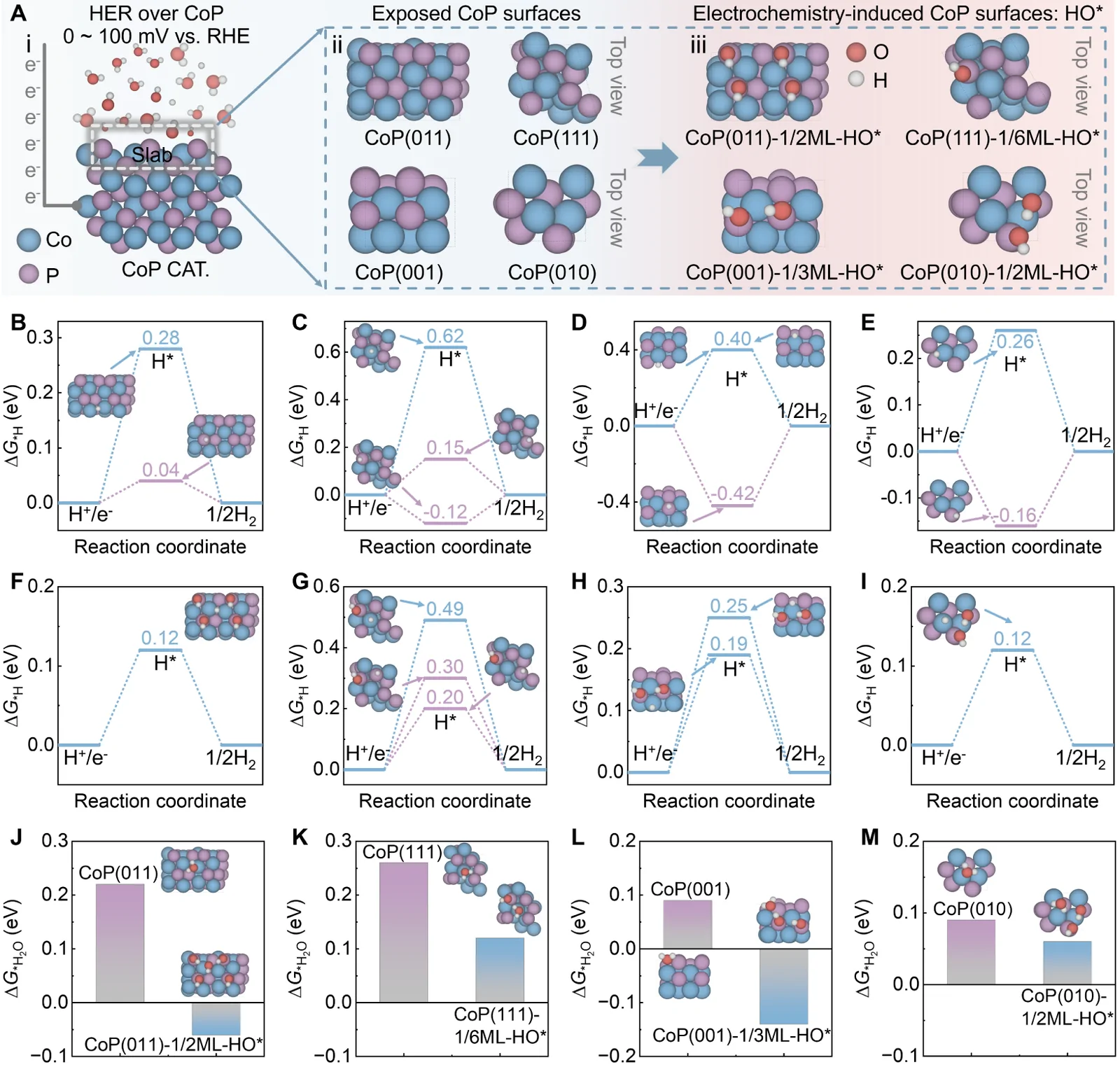
Influence of adsorbed HO* on the HER structure–activity relationship and H_2_O adsorption at low overpotentials. A, Surface HO*-covered configurations at the low overpotential window (0–100 mV). B–E, Free energy evolutions of HER on various pristine CoP surfaces, including (B) CoP(011), (C) CoP(111), (D) CoP(001), and (E) CoP(010). F–I, Free energy evolutions of HER on various HO*-covered CoP surfaces, including (F) CoP(011)-1/2 ML-HO*, (G) CoP(111)-1/6 ML-HO*, (H) CoP(001)-1/3 ML-HO*, and (I) CoP(010)-1/2 ML-HO*. J–M, Comparison of the calculated H_2_O adsorption free energies on the pristine versus the *operando* HO*-covered CoP surfaces: (J) CoP(011), (K) CoP(111), (L) CoP(001), and (M) CoP(010). The insets in Figure 3B–M illustrate the corresponding top-view atomic models.

### Effects of Surface Coverage and Electric Field on Surface Adsorbed Hydrogen under High Overpotentials

Interfacial electric fields fundamentally dictate the pH-dependence of electrocatalytic systems by modulating the binding energetics of reaction intermediates, a process highly sensitive to their inherent dipole moments (μ) and polarizabilities (α).^20,25,40^ Therefore, explicitly incorporating these field effects is indispensable for a rigorous evaluation of CoP HER activity, particularly within the elevated overpotential regime (high η_10_ window: 100 < η_10_ < 200 mV). Furthermore, the electric field effect varies across different resting surfaces due to the changes in both surface coverage-induced dipole moments and surface adsorption configurations. The elevated overpotential regime (100 < η_10_ < 200 mV) drives a structural evolution of the catalyst surface, transitioning from an HO*-covered configuration to an H*-dominated resting state, as exemplified in Figure 4A. This potential-induced evolution yields a densely H*-populated CoP surface, characterized by the explicit formation of Co–H and P–H bonds. Extensive surface coverage fundamentally modulates the hydrogen adsorption free energy. As illustrated in Figure 4B–E, evaluating the coverage-dependent Δ*G*_H*_ across the CoP(011), (111), (001), and (010) facets, reveals a systematic trend: elevated H* coverages universally weaken the hydrogen binding strength across the catalyst surface. Remarkably, under the elevated overpotential regime, the specific *operando* surface coverages, namely, CoP(011)-1/1 ML-H*, CoP(111)-2/3 ML-H*, CoP(001)­1/1 ML-H*, CoP(010)-1/2 ML-H*, and CoP(010)-1/1 ML-H*, precisely tune the Δ*G*_H*_ into the optimal thermodynamic window of −0.1 to 0.1 eV. This near-thermoneutral binding energetics mechanistically dictates the exceptional macroscopic HER activity of CoP. Furthermore, at such high H* coverage conditions, Figure 4F–I and Supplementary Figures 17–18 demonstrate how H* intermediates interact with different electric fields. We observed that both μ and α of H* are relatively small over different CoP surfaces, suggesting a small electric field effect. This is mainly because adsorbed H* has the compact atomic volume, consequently leading to a smaller adsorption-induced dipole moment. While the perturbation of the electric field on H* adsorption is relatively small, incorporating this parameter is nonetheless essential for achieving precise thermodynamic evaluation at high overpotentials. Figure 4J–M and Supplementary Figure 18B further delineate the pH-dependent HER energetics as a function of applied overpotential. At a constant overpotential, an increase in pH systematically strengthens H* adsorption, yielding favorable Δ*G*_H*_ values at elevated overpotentials of 150 and 200 mV. Prompted by our large-scale data mining, which reveals a distinct subset of CoP-based electrocatalysts operating with η_10_ between 200 and 400 mV (Figure 1B–C and Supplementary Figures 2–3), we extended our analysis to higher overpotential regimes (300 and 400 mV). This extended evaluation demonstrates that while operational overpotentials predictably boost H* adsorption affinity, the Δ*G*_H*_ values across the four predominantly exposed CoP facets remain remarkably pinned near the Sabatier optimum (Δ*G*_H*_ = 0 eV) across the wide pH range of 0 to 14. Ultimately, this thermodynamic resilience under coupled pH and electric field effects fundamentally elucidates the intrinsic origin of the exceptional HER activity on CoP.

**Figure 4:**
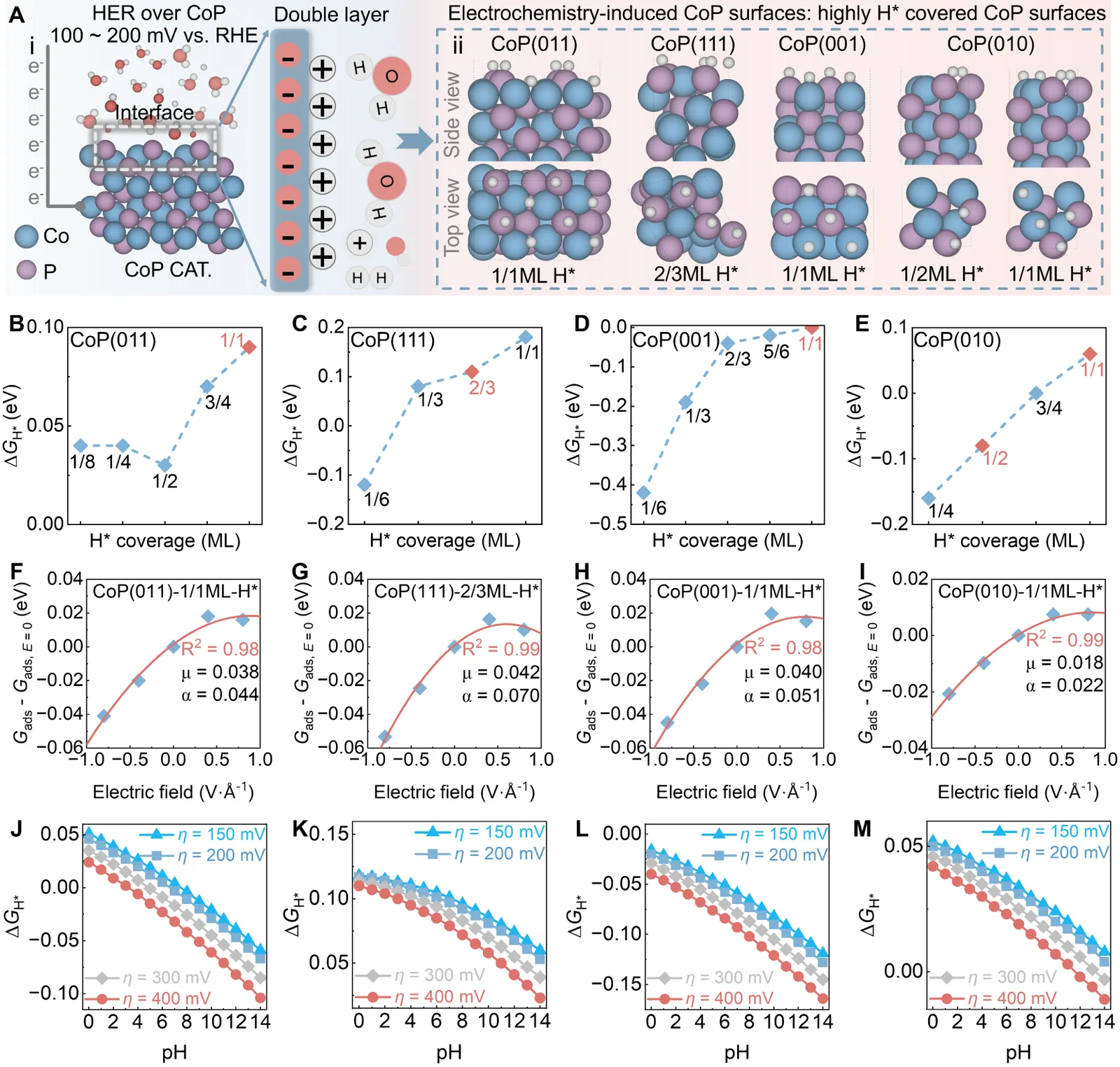
Influence of the electric field on the HER structure–activity relationship at high overpotentials. A, H*-covered surfaces at the high overpotential window (100–200 mV). B–E, The adsorption free energy (Δ*G*_H*_) of H* on (B) CoP(011), (C) CoP(111), (D) CoP(001), and (E) CoP(010) under different coverages. F–I, Electric field effect on the adsorption of H* on (F) CoP(011)-1/1 ML-H*, (G) CoP(111)-2/3 ML-H*, (H) CoP(001)-1/1 ML-H*, and (I) CoP(010)-1/1 ML-H* with the fitted values for μ (dipole moment, e·Å) and α (polarizability, e^2^·V^–1^). J–M, Δ*G*_H*_ of H* on (J) CoP(011)-1/1 ML-H*, (K) CoP(111)-2/3 ML-H*, (L) CoP(001)-1/1 ML-H*, and (M) CoP(010)-1/1 ML-H* under different overpotentials.

### Formation of Surface Phosphorus Vacancies and the HER Volcano Model

High stability is one of core requirements for highly active CoP catalysts. Introducing phosphorus vacancies can improve the HER performance through activating neighboring cobalt active sites.^12,41^ However, the introduction of phosphorus defects will reduce the stability of the CoP-based material, which may lead to the continuous collapse of the CoP catalyst under a strong electric field. To rigorously assess the electrochemical stability of CoP and preclude the spontaneous formation of phosphorus vacancies (P_V_*) under applied electric fields, we constructed surface Pourbaix diagrams for six distinct facets (Figure 5A–F). These include the thermodynamically favored CoP(011), (111), (001), and (010) planes, alongside the experimentally observed (101) and (211) surfaces.^42–44^ Our thermodynamic analysis reveals that P_V_* generation is strictly confined to extreme overpotential regimes. Specifically, among them, even the most susceptible facets, CoP(111) and CoP(101), require profound cathodic potentials of −1.3 and −1.8 V_RHE_, respectively, to induce P depletion. Crucially, the predominantly exposed CoP(011) surface demonstrates exceptional resilience, requiring an applied potential exceeding −2.0 V_RHE_ to form surface vacancies. Together, these surface Pourbaix diagram analyses definitively confirm the intrinsic operational stability of CoP, ruling out spontaneous defect generation of P_V_* in the absence of intentional external perturbation. For comparison, we evaluated the average surface P vacancy formation energy (Δ*E*_f_) of CoP relative to various P reference states. Regardless of the chosen reference state, most CoP surfaces exhibit high Δ*E*_f_ values (Supplementary Figure 20), indicating that the formation of surface P vacancies is thermodynamically unfeasible. Furthermore, we analyzed the formation thermodynamics of P–nH* (n = 1, 2, 3) species over CoP (Supplementary Figures 21–22), finding that only the P–1H* species is energetically favorable. Additionally, from the kinetics perspective, we investigated the P migration barriers with the adsorption of hydrogen (H*) on P sites (P–0H* → P–1H* → P–2H* → P–3H*) (Supplementary Figure 23). At low H* coverage (e.g., P–1H*), the P migration barrier is very high, and remains largely unaffected or even increases (Supplementary Figure 23A and D). Only at higher H* coverages (P–2H* and P–3H*) does the surface P stability decline alongside a reduced activation barrier. However, the formation of P–2H* and P–3H* species is thermodynamically unfeasible (Supplementary Figure 21). Consequently, the formation of surface P vacancies (P_V_*) is thermodynamically and kinetically precluded under typical HER operating conditions.

**Figure 5:**
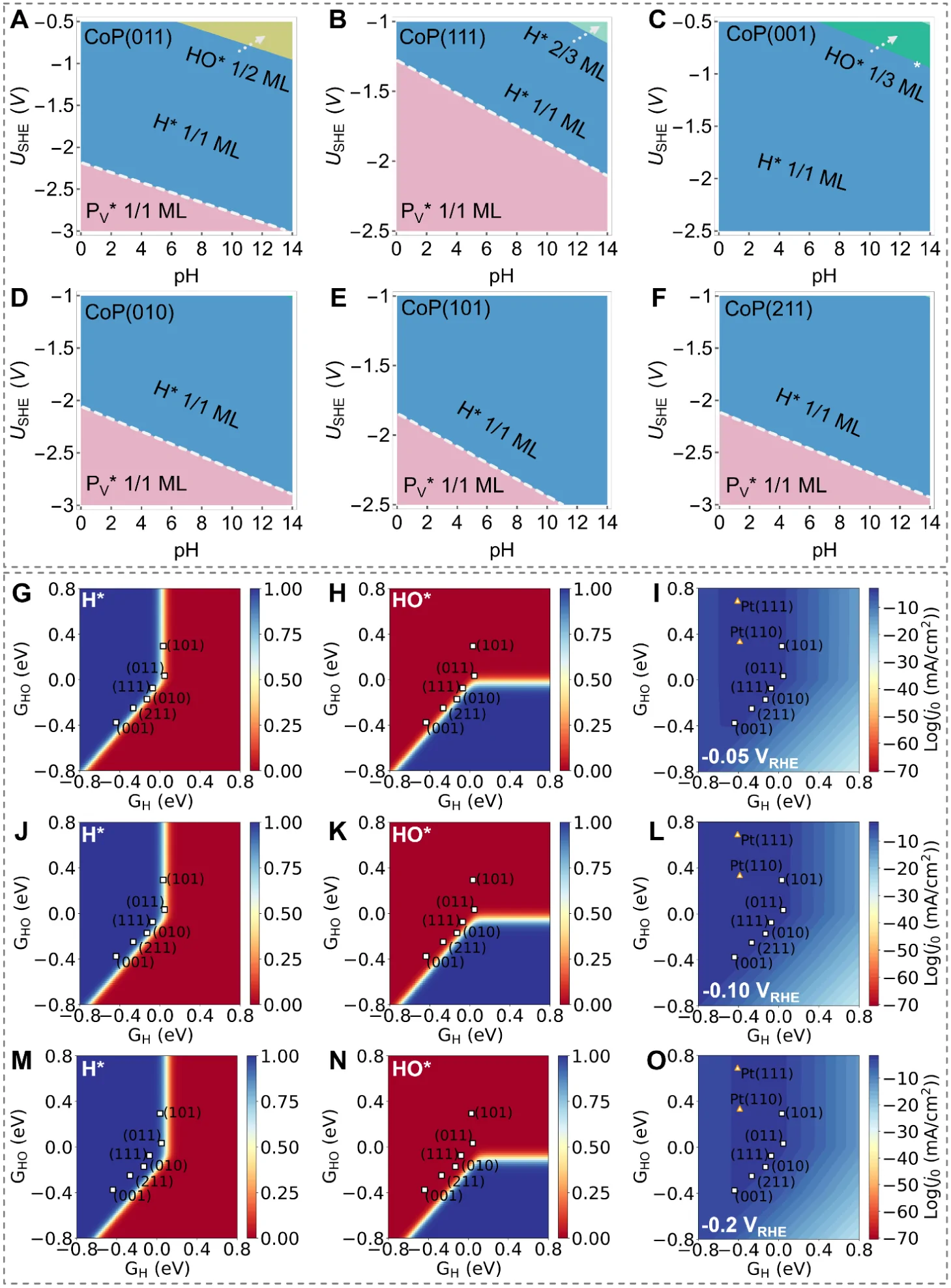
The surface stability and volcano activity prediction based on Δ*G*_H*_ and Δ*G*_HO*_. A–F, Analysis of surface phosphorus vacancies through surface Pourbaix diagrams, including (A) CoP(011), (B) CoP(111), (C) CoP(001), (D) CoP(010), (E) CoP(101), and (F) CoP(211). G–I, The coverages of (G) H* and (H) HO*, (I) 2D microkinetic volcano of HER for CoP and Pt at −0.05 V_RHE_. J–L, The coverages of (J) H* and (K) HO*, (L) 2D microkinetic volcano of HER for CoP and Pt at −0.10 V_RHE_. M–O, The coverages of (M) H* and (N) HO*, (O) predication of volcano activity for CoP and Pt at −0.20 V_RHE_.

According to our previous research,^32^ in the electrochemical interface environment, there is competition between the adsorption of HO* and H*, which synergistically affects the surface coverage and HER activity. Therefore, we further employed the state-of-the-art theoretical models developed with *G*_H*_ and *G*_HO*_ as dual-descriptors to predict the H* and HO* coverages on different CoP surfaces at different external potentials, as well as the HER activities of different CoP surfaces (Figure 5G–O). It can be found that at a low external potential (−0.05 V_RHE_), the CoP surface is mainly covered by HO* and a small amount of H*, which is consistent with our surface Pourbaix diagram analysis (Figure 5G–H). As the potential gradually decreases, the surface of CoP is gradually dominated by H*. Especially at a high external potential (−0.20 V_RHE_), all surfaces of CoP are occupied by H* (Figure 5M–N). Further prediction using the activity volcano model shows that at different potentials, the HER activities of different CoP surfaces and Pt(111) and (110) are always near the apex of the volcano (Figure 5I, L, and O), indicating that CoP has an excellent activity at different overpotentials comparable to the noble Pt electrocatalysts.

### Electrochemical HER and Identification of the Surface Species by *In Situ* Characterizations

To validate the mechanistic insights derived from our computational models, we synthesized CoP electrocatalysts for subsequent HER evaluation. To ensure a rigorous correlation between theory and experiment, it is first imperative to confirm that the bulk and surface properties of the as-synthesized CoP material align with our theoretical model. The powder X-ray diffraction (XRD) pattern (Figure 6A) of as-synthesized CoP samples has demonstrated the characteristic diffraction peaks of the pure orthorhombic CoP phase (PDF#29-0497) and confirms that the bulk sample consists of high-purity MnP-type CoP.^9,10,38^ The bulk structure and morphology of as-synthesized CoP have been characterized by the scanning electron microscopy (SEM) and the high-resolution transmission electron microscopy (HRTEM), demonstrating the perfect nanosheet structure and clear lattice distances of different exposed CoP surfaces, like CoP(011) (Figure 6B–C, and Supplementary Figure 25). To gain deeper insights into the electronic structure and local coordination environment of the as-synthesized CoP samples, the synchrotron-based X-ray absorption spectroscopy (XAS) was performed. The Co K-edge X-ray absorption near-edge structure (XANES) spectra show an absorption edge for CoP positioned between those characteristics of metallic Co foil (Co^0^) and cobalt oxides (e.g., CoO, CoOOH), deducing that the Co atoms of CoP bear a partial positive charge of +1.3 due to the electron transfer from Co to P (Figure 6D and Supplementary Figures 26A–B). The Bader charge analysis of CoP unit cell also demonstrates the positive charge of Co with the value of +0.21e (Supplementary Figure 26C), in accordance with the experimental XANES-deduced trend. It should be noted that the Bader charge is different from the valence state in nature. The Bader charge reflects electrons carried by atoms while the valence state is a formal, theoretical property denoting an atom’s assigned combining number or charge within a compound. Therefore, Bader charge and valence state are fundamentally distinct: the former is a continuous measure of atomic charge, while the latter is a discrete, formal oxidation state. The structural evolution has been further scrutinized using the extended X-ray absorption fine structure (EXAFS) analysis. In the *k* ^2^-weighted Fourier-transformed (FT-EXAFS) spectra (Figure 6E), CoP exhibits a dominant peak at approximately 1.71 Å (without phase shift correction), which is unambiguously assigned to the Co–P scattering path. Notably, the absence of a significant peak at approximately 2.17 Å (corresponding to the Co–Co metallic bond in Co foil) confirms the absence of metallic Co clusters, indicating the high purity of the synthesized CoP phase. The *k*-space EXAFS oscillations (Supplementary Figure 27A) further illustrate the distinct coordination features of the synthesized CoP compared to the reference samples (Co foil, CoO, CoPc, CoOOH, and Co_3_O_4_). To further distinguish the scattering signals in both *k* and *R* spaces, the wavelet transform (WT) analysis was employed (Figure 6F and Supplementary Figure 27B–F). The WT contour plot of the synthesized CoP identifies a pronounced intensity maximum at the *k* of ∼5.4 Å^–1^, consistent with the characteristic signature of Co–P coordination (Figure 6F). The WT maximum for metallic Co foil, however, occurs at a markedly higher *k* (∼7.8 Å^–1^), reflecting the heavy Co–Co scattering (Supplementary Figure 27B). In comparison, Co_3_O_4_ displays WT features typical of Co–O scattering at lower *R* and *k* (Supplementary Figure 27F).

**Figure 6:**
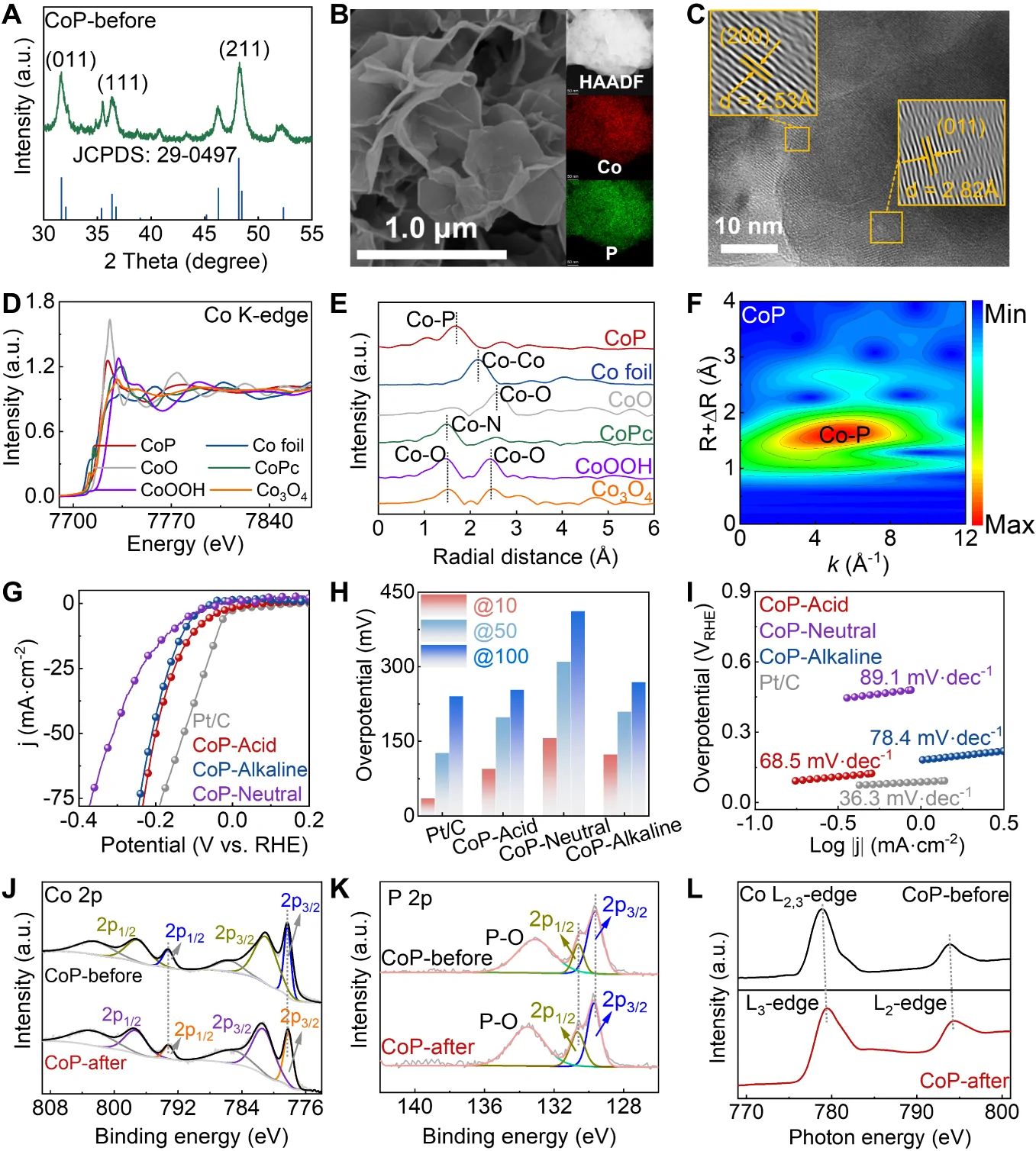
Structural characterization, pH-universal electrocatalytic HER performance, and operational stability of the synthesized CoP catalyst. The (A) XRD, (B) SEM, and (C) HRTEM characterizations of CoP-before samples. D, Normalized Co K-edge XANES spectra of CoP and reference samples (Co foil, CoO, CoPc, CoOOH, and Co_3_O_4_). E, Fourier-transformed (FT) *k* ^2^-weighted χ­(*k*) spectra in R space. F, Wavelet transform (WT) contour plots for the *k* ^2^-weighted EXAFS signals of CoP. G, Polarization curves (LSV) of CoP in acidic (0.5 M H_2_SO_4_), alkaline (1.0 M KOH), and neutral (1.0 M PBS) electrolytes, with the Pt/C electrode as a reference. H, Comparison of overpotentials at the current densities of 10, 50, and 100 mA·cm^–2^ for as-synthesized CoP in different electrolytes with the Pt/C electrode as a reference. I, Tafel plots derived from the polarization curves for CoP in different electrolytes. J–K, XPS spectra of (J) Co 2p and (K) P 2p for the CoP catalyst before and after the stability test of 24 h HER in the acidic electrolyte. L, Co L_2,3_-edge soft X-ray absorption spectroscopy (*s*XAS) of CoP before and after the stability test of 24 h HER.

Then, the electrocatalytic HER performance of as-prepared CoP nanosheet catalysts was evaluated in the acidic (0.5 M H_2_SO_4_), neutral (1.0 M PBS), and alkaline (1.0 M KOH) electrolytes using a typical three-electrode system (Figure 6G and Supplementary Figure 28). The overpotential at 10 mA·cm^–2^ (η_10_) is one of vital metrics for the evaluation of HER performance. According to *iR*-corrected linear sweep voltammetry (LSV) curves, the as-synthesized CoP exhibits the best electrocatalytic activity with a lower η_10_ of 95 mV under the acidic environment, whereas the η_10_ values are 157 and 124 mV under neutral and alkaline conditions, respectively. Figure 6H further exhibits the overpotentials at higher current densities of 50 and 100 mA·cm^–2^, comprehensively demonstrating the high activity of CoP electrocatalysts toward HER in different electrolytes. Meanwhile, its η_10_ values also agree well with the large-scale data mining of η_10_ on previous CoP-relevant reports (Figure 1B, and Supplementary Figures 2–3). To further confirm HER activities of CoP, the Tafel slopes of CoP under different aqueous electrolytes were analyzed by plotting the overpotential against log­(j) according to the Tafel equation (η = *b* × log­(j) + *a*) (Figure 6I). The Pt/C electrode, as a comparison, displays a Tafel slope of 36.3 mV·dec^–1^ at the electrolyte of 0.5 M H_2_SO_4_, which is similar to the previous reported values.^9,38,45–47^ CoP exhibits a markedly lower Tafel slope (68.5 mV·dec^–1^) in acidic electrolyte compared to neutral (89.1 mV·dec^–1^) and alkaline (78.4 mV·dec^–1^) electrolytes, indicating accelerated hydrogen evolution kinetics under acidic conditions. Exceptional operational stability sustained over a wide potential range at 10 and 100 mA·cm^–2^ also demonstrates the catalyst’s robustness across acidic, neutral, and alkaline electrolytes (Supplementary Figure 29). Furthermore, the acid-tested CoP electrocatalyst, exhibiting optimal HER activity at 10 mA·cm^–2^, underwent rigorous post-HER analyses of its crystallographic, morphological, surface lattice, and electronic properties (Supplementary Figures 30–31 and Figure 6J–L). First, retained crystallinity in post-HER CoP (named as “CoP-after”) is confirmed by persistent XRD reflections, indicating structural robustness (Supplementary Figure 30A). Concurrently, SEM imaging reveals preservation of the flake-like morphology (Supplementary Figures 30B and 31A), and elemental mapping confirms the near absence of surface oxidation. The well-resolved surface lattice observed via HRTEM also demonstrates remarkable structural stability (Supplementary Figures 30C and 31B). The thermogravimetric analyses (TGA) with the N_2_ atmosphere also further demonstrate the bulk stability after HER (Supplementary Figure 32). Then, the post-HER XPS analysis reveals that there is no marginal positive shift in the Co 2p_1/2_ and Co 2p_3/2_ binding energies of CoP electrocatalysts, evidencing minimal chemical-state perturbation of Co sites under operational conditions (Figure 6J). The P 2p (2p_1/2_ and 2p_3/2_) peaks exhibit a relatively small positive shift, indicative of a slightly elevated oxidation state of P at the catalyst surface (Figure 6K). This phenomenon may be attributed to the formation of P–O­(H) bonds via surface reaction during the HER at η_10_,^48^ which is also suggested by theoretical surface state analyses (Figures 2 and 5). This will be further discussed in the following *in situ* analyses. Notably, the Co 2p and P 2p XPS spectra exhibit no appreciable changes after the stability test, indicating that the CoP catalyst remains chemically stable and does not undergo oxidation during HER. The observed P–O signal is attributed to surface oxidation arising from incidental exposure of the catalyst to air, rather than electrochemically induced oxidation. Finally, complementary Co L_2,3_-edge soft X-ray absorption spectroscopy (*s*XAS) of the CoP-after sample reveals a marginal photon energy shift relative to that of the pristine CoP (CoP-before), further confirming negligible changes in the chemical state of Co (Figure 6L). Therefore, these results demonstrate slightly elevated surface valence states with preserved bulk electronic structure during HER at η_10_.

*In-situ* Raman and attenuated total reflectance-surface enhanced infrared absorption spectroscopy (ATR-SEIRAS) were employed to probe surface coverage species on CoP during HER. Figure 7A illustrates the potential-dependent intensity variations of the Raman bands associated with the P–O­(H) group in a 0.5 M H_2_SO_4_ solution. Due to interfacial adsorption, the characteristic vibrational peaks of the HSO_4_^–^ and SO_4_^2–^ species emerge between ∼850 and 1100 cm^–1^,^49,50^ inherently overlapping with the Raman signals of the P–O bonds.^51,52^ Despite this spectral interference, a gradual increase in the Raman intensity of the ν­(HSO_4_^–^) mode is observed as the applied potential decreases. This trend is because at low overpotentials, the P–OH groups can consume interfacially adsorbed HSO_4_^–^, leading to a diminished local concentration of these anions at the electrocatalytic interface. Conversely, at high overpotentials, the surface covered H* species, originated from the structural conversion of P–OH moieties to P–H species, can suppress HSO_4_^–^ dissociation, consequently increasing its Raman signal at the electrocatalytic interface. Furthermore, the absence of discernible Raman characteristic peaks at ∼600 cm^–1^, which correspond to Co–O vibrational modes,^51,52^ indicates that no surface oxidation occurs during the HER, in accordance with experimental characterizations of CoP-after samples. Complementary *in situ* ATR-SEIRAS spectra (Figure 7B–C) were also recorded to track the P–O­(H) and P–H vibrational modes.^53,54^ The weak P–O feature observed in Figure 7B again corroborates that minimal phosphorus oxidation occurs. Crucially, Figure 7C exhibits progressive emergence of a P–H stretching mode with increasing overpotential, indicative of H* adsorption and the formation of subsequent P–H bonds. These combined *operando* analyses validate theoretical predictions, demonstrating potential-dependent surface configurations of CoP surfaces during the HER. Furthermore, *in situ* Raman spectroscopy and ATR-SEIRAS were conducted in both neutral (1.0 M PBS) and alkaline (1.0 M KOH) electrolytes (Supplementary Figures 33–34). Although P–(O)H Raman signals are not resolved, likely due to electrolyte interference (Supplementary Figures 33A and 34A), the Co–O region provides critical insight into the catalyst’s interface against the electrolyte. Co–O Raman peaks are entirely absent in the alkaline electrolyte (Supplementary Figure 34A). While trace Co–O signals are detected across various overpotentials in the neutral electrolyte (Supplementary Figure 33A), these are attributed to incidental air exposure during sample handling rather than electrochemically induced oxidation. Together, these findings indicate that the CoP surface resists oxidation during the HER. This conclusion is further corroborated by *in situ* ATR-SEIRAS measurements, where the negligible intensity of the P–O peaks confirms that surface oxidation remains minimal under operating conditions (Supplementary Figures 33B and 34B). Importantly, as the overpotential increases, the *in situ* ATR-SEIRAS spectra have detected the P–H band (Supplementary Figures 33C and 34C). This confirms that the surface is covered with H* at high overpotentials, mirroring the behavior observed in the acidic electrolyte (0.5 M H_2_SO_4_). To unambiguously verify the assignment of the P–H vibrational modes, isotopic labeling experiments were performed using 1.0 M KOH in D_2_O (Supplementary Figure 35). Upon isotopic substitution, the characteristic P–H stretching band between 2300 and 2400 cm^–1^ diminishes, with the concomitant emergence of a new P–D stretching peak at ∼1650 cm^–1^. This distinct isotopic shift definitively confirms the generation of P–H intermediates on the CoP surface at high overpotentials. Note that *in situ* ATR-SEIRAS investigations in acidic and neutral media were precluded by the unavailability of deuterated H_2_SO_4_ and PBS electrolytes.

**Figure 7:**
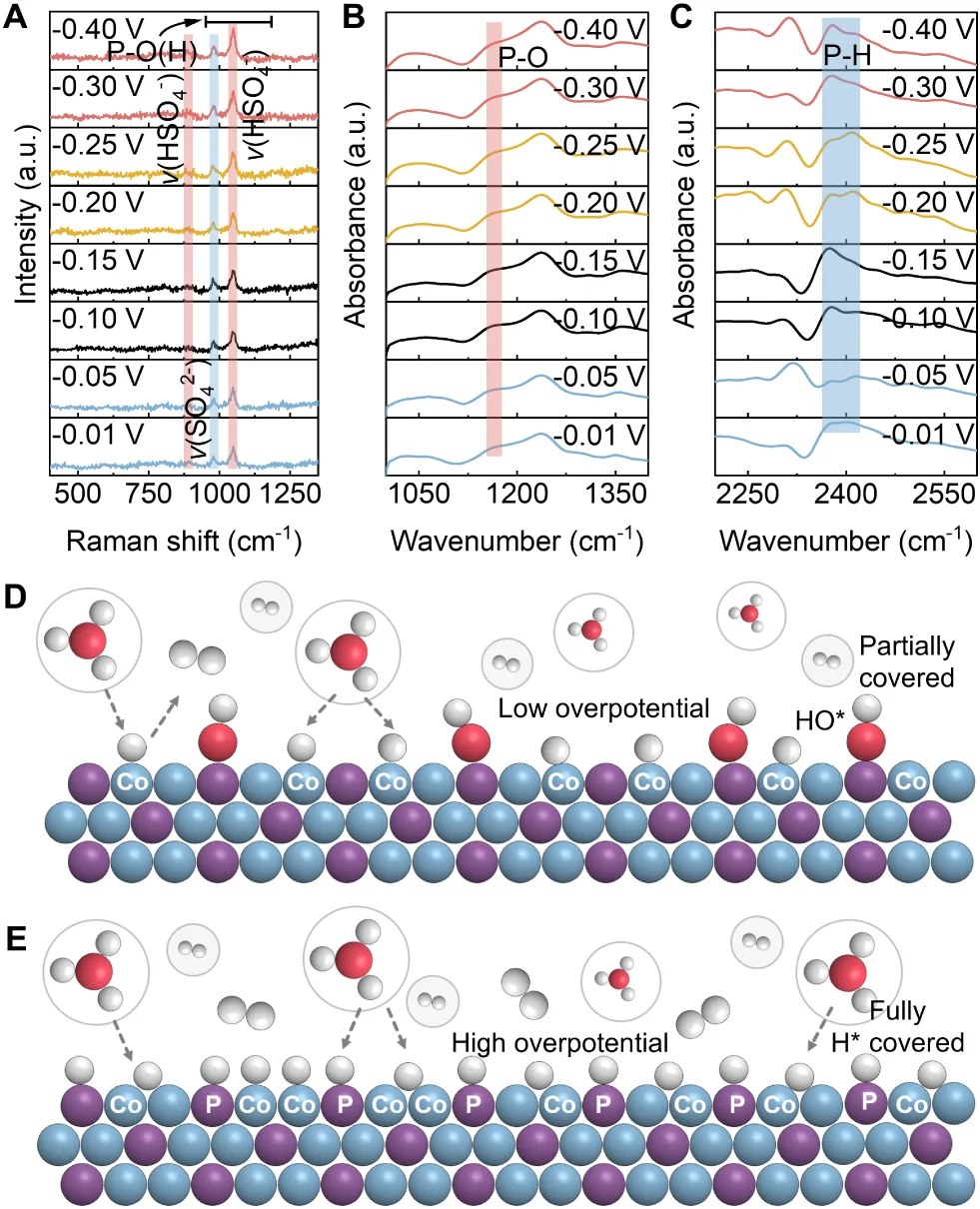
*In-situ* Raman and ATR-SEIRAS characterizations and work mechanism of CoP nanosheets toward HER. *In-situ* (A) Raman and (B–C) ATR-SEIRAS characterizations for the surface P–O­(H) and P–H groups at different overpotentials under the acidic (0.5 M H_2_SO_4_) environment. D–E, Schematic diagrams of work mechanisms over CoP under (D) low and (E) high overpotentials, indicating different active sites accountable for HER.

To demonstrate that surface-adsorbed species protect the catalyst against surface oxidation and subsequent elemental leaching during HER, we evaluated the structural and chemical stability of the as-synthesized CoP. We compared CoP samples subjected to a 24-h static immersion in acidic (0.5 M H_2_SO_4_), neutral (1.0 M PBS), and alkaline (1.0 M KOH) electrolytes (“Electrolyte-1”) against those operated under HER conditions at 10 mA·cm^–2^ for 24 h (“Electrolyte-2”) (Supplementary Figures 36–38). Notably, the surface morphology of CoP is well-preserved under active HER conditions (Supplementary Figure 37), whereas the statically immersed samples exhibit partial morphological degradation (Supplementary Figure 36). Furthermore, inductively coupled plasma mass spectrometry (ICP-MS) reveals that the concentrations of dissolved Co and P in “Electrolyte-2” are negligible, remaining significantly lower than those detected in “Electrolyte-1” (Supplementary Figure 38 and Supplementary Table 5). These results strongly suggest that the surface-adsorbed species effectively shield the CoP from electrolyte-induced oxidation and leaching. To evaluate the potential for industrial application, we also performed extended chronopotentiometry testing of CoP at an industrially relevant current density (>1 A·cm^–2^) for 24 h. As shown in Supplementary Figure 39, the catalyst maintains highly stable electrochemical performance across electrolytes with different pH values throughout the testing period. To unequivocally decouple intrinsic catalyst degradation from morphological surface area loss, we evaluated the double-layer capacitance (Cdl) and the corresponding electrochemically active surface area (ECSA) before and after rigorous stability tests (>1 A·cm^–2^) for 24 h. The results (Supplementary Figure 40) demonstrate that both Cdl and ECSA remain virtually unchanged, underscoring the robust structural integrity of the CoP catalyst under harsh operating conditions. Furthermore, ECSA-normalized polarization curves recorded before and after the extended high-current-density (>1 A·cm^–2^) operation nearly overlap (Supplementary Figure 41). This confirms that the intrinsic catalytic activity of the individual active sites is fully preserved. Collectively, these findings validate the proposed protection mechanism of surface adsorbed species and highlight the industrial viability of CoP-based electrocatalysts. Figure 7D–E displays the schematic diagrams of potential-dependent surface configurations for CoP during HER, where at low overpotentials (0–100 mV), HO*-covered surfaces dominate with the formation of P–OH groups and Co atoms act as main active sites for HER, while high overpotentials (>100 mV) generate H*-dominated surfaces with the formation of P–H and Co–H bonds. The resting surface under *operando* conditions not only has high activity for HER but also prevents surface oxidization and then structural collapse for high stability.

## Conclusions

In summary, this work resolves the origin of the high HER activity and structural stability of CoP electrocatalysts. By integrating large-scale data mining (2,282 experimental observations based on ∼886 CoP-based catalysts), AI-driven catalysis platform (i.e., the *DigCat*) with state-of-the-art simulations, we elucidate the long-debated relationship between the changing surface structure of CoP and its catalytic performance. We reveal that at low overpotentials (0 to 100 mV), the exposed CoP surfaces are stabilized by HO* adsorbates and the subsequent formation of P–OH groups. This configuration critically modulates the electronic properties of the Co active sites, boosting water activation and thereby enhancing HER performance. Conversely, at high overpotentials (>100 mV), the surface transitions to a highly H*-covered state, which delivers a highly favorable ΔG_*H_ under the influence of the interfacial electric field. Notably, these dynamic, potential-driven surface adaptations protect the CoP structure from destructive oxidation during catalysis, while electrochemically driven phosphorus vacancy formation is shown to require prohibitively high overpotentials. The successful capture of these potential-dependent surface species *via in situ* Raman and ATR-SEIRAS characterizations provides robust, direct confirmation of our theoretical framework. The established knowledge on HER over CoP electrocatalysts is transferable for other electrocatalytic systems. Broadly, this work not only resolves the changing active structure and stability mechanisms of the prominent CoP electrocatalyst, but also showcases the remarkable capabilities of combining AI-driven data platforms and precise simulations in identifying fundamental mechanistic challenges, as well as in expediting the design of efficient electrocatalysts.

## Experimental Section

### Computational Details

All spin-polarized calculations were conducted out with the Vienna *Ab Initio* Simulation Package (VASP). The Projector Augmented Wave (PAW) method was used to describe electron–ion interactions.^55,56^ The exchange-correlation effects of electrons were treated with the revised Perdew–Burke–Ernzerhof (RPBE) functional,^57^ a generalized gradient approximation (GGA). The kinetic energy cutoff for the plane-wave basis set was set to 520 eV. The Monkhorst–Pack scheme with the k-point separation of 0.04 Å^–1^ was used to sample the Brillouin zone.^58^ The dispersion interaction was described by the DFT-D3 scheme, proposed by Grimme.^59,60^ All geometry optimizations were carried out until the residual forces on the system converged below 0.03 eV/Å, with the electronic self-consistent (SC) loop convergence threshold set to 1 × 10^–5^ eV. More details are illustrated in Supplementary Texts 1–7.

### Chemicals and Materials

Cobalt­(II) nitrate hexahydrate (Co­(NO_3_)_2_·6H_2_O, ≥99.0%), sodium nitrate (NaNO_3_, ≥99.0%), and sodium hypophosphite monohydrate (NaH_2_PO_2_·H_2_O, ≥98.0%) were purchased from Aladdin Industrial Corporation (Shanghai, China). Commercial Pt/C catalyst (20.0 wt % Pt) and Nafion perfluorinated resin solution (5.0 wt %) were also obtained from Aladdin. For the electrochemical measurements, sulfuric acid (H_2_SO_4_, 95.0–98.0%), potassium hydroxide (KOH, ≥85.0%), and phosphate-buffered saline solution (PBS, 1.0 M, pH = 7.2–7.4) were used as electrolytes, all acquired from Aladdin. Other reagents used for cleaning and preparation, including hydrochloric acid (HCl, 36.0–38.0%), acetone (≥99.5%), and ethanol (≥99.7%), were of analytical grade. Deionized (DI) water (18.2 MΩ·cm) was used throughout all experiments. All chemicals were used as received without further purification. The synthesis and characterization of CoP nanosheets were shown in Supplementary Texts 8–9.

### Electrochemical Measurements

The working electrode was prepared utilizing a drop-casting method. Typically, 5 mg of the catalyst powder was dispersed in a mixture of 750 μL of deionized water, 250 μL of ethanol, and 20 μL of Nafion solution (5 wt %) by ultrasonication for at least 30 min to form a homogeneous ink. Subsequently, a specific amount of the ink (e.g., 5 μL) was loaded onto a polished glassy carbon electrode (GCE, diameter: 3 mm) and dried naturally at room temperature to obtain a uniform catalyst film. Electrochemical measurements were performed on a CHI 660E electrochemical workstation (CH Instruments, Inc., Shanghai) using a standard three-electrode configuration with a graphite rod as the counter electrode. The HER catalytic activities were evaluated in acidic (0.5 M H_2_SO_4_), neutral (1.0 M PBS), and alkaline (1.0 M KOH) electrolytes, respectively. Specifically, an Ag/AgCl electrode (saturated KCl) served as the reference electrode for the measurements in both H_2_SO_4_ and PBS solutions, whereas an Hg/HgO electrode (1.0 M KOH) was adopted in the 1.0 M KOH solution. Linear sweep voltammetry (LSV) curves were recorded at a scan rate of 5 mV s^–1^. All potentials were calibrated to the reversible hydrogen electrode (RHE) scale according to the Nernst equation (E_RHE_ = E_ref_ + 0.059 × pH + E^0^_ref_), and all polarization curves were corrected for the ohmic potential drop (*iR*) losses within the electrolyte to reflect the intrinsic catalytic activity. Tafel slopes were derived from steady-state currents measured during controlled-potential electrolysis. The electrochemically active surface area (ECSA) was determined from the scan-rate dependence of cyclic voltammograms (CVs) recorded between 10 and 50 mV·s^–1^. Long-term durability was evaluated via chronopotentiometry over a 24 h period. Electrochemical impedance spectroscopy (EIS) measurements were carried out in a three-electrode configuration. Impedance spectra were collected at the HER operating potential with a 5 mV AC perturbation over the frequency range of 100 kHz to 0.1 Hz. Nyquist plots were fitted using an equivalent circuit composed of Rs (R_1_) in series with a parallel R_ct_ (R_2_)–CPE element. All impedance data were analyzed using ZView software under identical fitting conditions. The extracted R_ct_ values were subsequently used to compare the charge-transfer kinetics of “CoP-Acid”, “CoP-Neutral”, “CoP-Alkaline”, and Pt/C catalysts.

## Supplementary Material



## Data Availability

All data supporting the results of this study are available within the paper and its Supporting Information. In addition, key computational and experimental data have been uploaded to the Digital Catalysis Platform (*DigCat*: www.digcat.org).

## Notes

Additional computational details, including surface energy calculations; surface Pourbaix diagrams; *Ab initio* molecular dynamics; surface electronic structure; the effect of electronic fields; pH-dependent activity at the RHE scale; the activity volcano model at the RHE scale; synthesis and characterization of CoP; and *in situ* characterizations of the evolution of CoP surface coverage during the HER (DOCX)
