# Supplementary material for: Operando Surface Dynamics Govern the pH-Universal Activity and Stability of Cobalt Phosphide for Hydrogen Evolution
Source: J Am Chem Soc. 2026 Oct 29;148(44):48463–77. doi: 10.1021/jacs.6c17411 (PMC13761278; doi:10.1021/jacs.6c17411)
Supplement: Supplementary file 1 [file ja6c17411_si_001.docx]

**Supplementary Information**

***Operando* Surface Dynamics Govern the pH-Universal Activity and Stability of Cobalt Phosphide for Hydrogen Evolution**

Zhongyuan Guo^1,2,3,#^, Yihong Yu^4,#^, Songbo Ye^3^, Du Chen^1,2^, Chaohuang Chen^1,2^, Xue Jia^3^, Di Zhang^3^, Li Wei^5^, Min Zhou^6,*^, Song Li^4,*^, Jiang Xu^1,2,7,*^ and Hao Li^3,*^

^1.^ State Key Laboratory of Soil Pollution Control and Safety, Zhejiang University, Hangzhou 310058, China

^2.^ College of Environmental and Resource Sciences, Key Laboratory of Environmental Remediation and Ecological Health, Ministry of Education, Zhejiang University, Hangzhou 310058, China

^3.^ Advanced Institute for Materials Research (WPI-AIMR), Tohoku University, Sendai 980-8577, Japan

^4.^ Key Lab for Anisotropy and Texture of Materials, School of Materials Science and Engineering, Northeastern University, Shenyang 110819, China

^5.^ School of Chemical and Biomolecule Engineering, The University of Sydney, Sydney, New South Wales 2006, Australia

^6.^ Key Laboratory of Electroanalytical Chemistry, Changchun Institute of Applied Chemistry, Chinese Academy of Sciences, Changchun 130022, China

^7.^ Innovation Center of Yangtze River Delta, Zhejiang University, Jiashan 314100, China

*** Corresponding authors**

E-mail: mzhou1982@ciac.ac.cn (M.Z.), [lis@atm.neu.edu.cn](mailto:lis@atm.neu.edu.cn) (S.L.), xujiang6@zju.edu.cn (J.X.), and li.hao.b8@tohoku.ac.jp (H.L.)

### Table of Contents

**Supplementary Text 1−8**	**3**

**Supplementary Figure 1−39**	**11**

**Supplementary Table 1−5**	**50**

**References**	**106**

### Supplementary Text

### Supplementary Text 1 Surface energy determination

To quantitatively and concisely obtain the surface energy (γ) for CoP, here the seven-layer (1×1) unit cell of the slab model was chosen to minimize the effect of crystal layers on γ values. Each slab surface was allowed to relax with the middle three-layer atoms fixed, that was, only the top and bottom two-layer atoms were relaxed during optimization. The surface energy is derived by

γ = 1/2A · (*E*_slab_ – N · *E*_bulk_)

where *E*_slab_ is the total energy of the optimized slab surface, *E*_bulk_ is the bulk energy per Co(P) atom, N is the number of atoms in the slab surface (here the number in the cleaved slab surface is Co_28_P_28_), and A is the surface area for the slab surface. The factor of 2 is because of two optimized surfaces, one is at the top and another at the bottom of the slab.

In this work, we chose seven possibly exposed facets of CoP crystals for the analysis of surface energy, that is, (100), (010), (001), (011), (111), (102), and (201), as shown in Supplementary Fig. 4. These facets considered have been observed in previous reports. However, their exposure exhibits variation across reported studies, likely resulting from different synthesis methods. Therefore, distinguishing thermodynamically stable surfaces with low energy configurations in orthorhombic CoP crystals and quantifying their surface energy differentials is essential. To achieve thermodynamically stable CoP surfaces, cleavage was performed along low-index planes with minimal dangling bond density, thereby reducing surface energy through controlled termination (i.e., P-terminated, Co-terminated, and mixed). It should be noted that the selected CoP(100) and (111) facets exhibit chemically distinct terminations, consequently, their computed surface energies (γ) represent averaged values across both upper and lower slab configurations. Although a simple mathematical γ average of an asymmetric slab does not represent the true thermodynamic surface energy of either specific termination, these selected asymmetrical configurations can ensure the low-index planes with minimal dangling bond density. In comparison, we also analyzed the symmetrical CoP(100) and (111), named as s-CoP(100) and s-CoP(111), respectively, which demonstrated the same energy trend although their specific values were different. Therefore, our selected CoP facets can mirror the surface exposure of CoP, and the surface energies of CoP surfaces follow the order of CoP(011) < CoP(111) < CoP(001) < CoP (010) < CoP(102) < CoP(201) < CoP(100). The computed surface energies for distinct CoP crystallographic facets were summarized in Supplementary Table 3.

### Supplementary Text 2 Surface Pourbaix and Ab Initio Molecular Dynamics

Before analyzing the surface Pourbaix, the CoP unit was fully optimized (Supplementary Table 2). To acquire the surface Pourbaix diagrams, five possible species were considered, including H*, O*, HO*, H_2_O*, and P vacancies (P_V_*). These species adsorption can be described as follows:

n·(H^+^ + e^–^) + * → n·H*

n·H_2_O + * → n·HO* + n·(H^+^ + e^–^)

n·H_2_O + * → n·O* + 2n·(H^+^ + e^–^)

3n·(H^+^ + e^–^) + n·*(P) → n·PH_3_ + n·P_V_*

The free energy (*G*) under different coverages were calculated based on the method described by Ref. ^1, 2^. Furthermore, the ab initio molecular dynamics (AIMD) simulations at 500 K for 100 ps with a time step of 1.0 fs were employed to confirm the stability of surface-covered species and the surface configurations. More computational details on the surface Pourbaix diagrams and surface reconstructions can refer to our previous reports.^3-5^

### Supplementary Text 3 Surface P Vacancy Formation Energy and Migration Barrier

The formation energy (∆*E*_f_) of P_V_* is defined as follows: ∆*E*_f_ = *E*(P_V_*) + *E*(PH_3_) – *E*(*) – 3 × *E*(H^+^/e^-^), where *E*(P_V_*), *E*(PH_3_), *E*(*), and *E*(H^+^/e^-^) represent the electronic energies of the P-defective surface, PH_3_ molecule, the pristine surface, and the proton-electron pair. The average ∆*E*_f_ is defined as [*E*(n × P_V_*) + n × *E*(PH_3_) – *E*(*) – 3n × *E*(H^+^/e^-^)]/n, where *E*(n × P_V_*) represents the electronic energy of the slab surface covered by n × Pv*. To make a comparison, the black P as a reference state was also employed for the analysis of ∆*E*_f_. The effect of H* adsorption on the P migration barrier is analyzed by the climbing image nudged elastic band method (Cl-NEB).

### Supplementary Text 4 Surface Electronic Structure of CoP

The effect of electrochemistry-induced surface coverage on the electronic structures of Co was analyzed by the density of sates (DOS) and the Bader charge. When analyzing the DOS, a denser Gamma-centered sampling of the Brillouin zone was used, including (3×5×1) for CoP(011), (6×5×1) for CoP(111), (7×5×1) for CoP(001), and (6×7×1) for CoP(010). These dense samplings can give the specified separation of 0.03 Å^-1^ between neighbouring grid points. However, the k-point separation of 0.04 Å^-1^ was used to sample the Brillouin zone for the geometry relaxation.

### Supplementary Text 5 Free Energy Calculation of Hydrogen Evolution Reaction

The adsorption of H* is the key for the hydrogen evolution reaction (HER). Usually, the adsorption free energy (∆*G*_H*_) of H* can determine HER activity. In acidic environments, H* can be from (H^+^+e^-^), while in neutral and alkaline environments, the H* is formed by the dissociation of H_2_O. Take the HER in the acidic environment [H^+^ + *e*^-^ + * → H* (acidic)], the ∆*G*_H*_ of H* was calculated as follows: ∆*G*_H*_ = *G*(H*) – *G*(*) – 1/2 × *G*(H_2_). Due to the change of H* coverage on the CoP under different overpotentials, the average of ∆*G*_H*_ could be calculated as follows: [*G*(nH*) – *G*(*) – n/2 × *G*(H_2_)]/n, where *G*((n)H*), *G*(*), and *G*(H_2_) represent the free energies of the covered surface, the clean surface and the H_2_ molecule.

The Gibbs free energy (G) of one species was defined as: *G* = *E* + *E*_zpe_ + $\int C_{p}$d*T* – *T* × *S*, where *E* and *E*_zpe_ mean the electronic energy and zero-point energy, respectively. The latter two terms are the corrections from the heat capacity ($\int C_{p}$d*T*) and entropy (*S*). The temperature (*T*) can be set to 298.15 K.

### Supplementary Text 6 Effect of Electronic Fields and pH-dependent Activity at the RHE Scale

A saw-tooth potential was employed to apply electric fields, spanning a range from -0.8 to 0.8 eV/Å. For each applied field, adsorbates were relaxed until a force convergence threshold of 0.05 eV/Å was achieved. To account for potential and pH dependence, we employed a parallel-plate capacitor model to correlate the electric fields ($\vec{E}$) with the standard hydrogen electrode (SHE) and reversible hydrogen electrode (RHE) potentials, which is described as follows: $\vec{E}$ = $\frac{\sigma}{\varepsilon\varepsilon_{0}}$ = $\frac{C_{H}(U_{SHE}-U_{PZC})}{\varepsilon\varepsilon_{0}}$, where σ denotes charge density, 𝜀₀ represents vacuum permittivity (8.85 × 10^-12^ F﹒m^-1^), 𝜀 signifies the dielectric constant (dimensionless), *C*_H_ denotes Helmholtz capacitance (μF﹒cm^-2^), *U*_SHE_ refers to the potential versus SHE, and *U*_PZC_ denotes the potential at the point of zero charge (PZC) versus SHE. More details can be found in our previous report.^6^

To evaluate the response of an adsorbate to the electric field, a second-order polynomial was fitted to the calculated data for each adsorbate across the range of electric fields. The equation below was then employed to extract the intrinsic dipole moment (μ) and polarizability (α): *G*_ads_ = $G_{ads}^{PZC}$ + μ$\vec{E}$ - $\frac{\alpha}{2}\vec{E}$^2^, where $G_{ads}^{PZC}$ is defined as the binding energy of the adsorbate at the potential of zero charge (PZC), corresponding to the energy calculated in the absence of an applied electric field. Additionally, *U*_SHE_ has a correlation with the *U*_RHE_: *U*_SHE_ = *U*_RHE_ - 𝑘_𝑏_﹒𝑇﹒𝑙𝑛(10) × 𝑝𝐻 = *U*_RHE_ − 0.059﹒𝑝𝐻.

### Supplementary Text 7 Activity Volcano Model at the RHE Scale (HER Microkinetic Modeling)

The hydrogen evolution reaction generally consists of three fundamental steps, as follows:

$$H^{+}+e^{-}\leftrightarrow H* \left( Volmer step \right)$$

$$H^{+}+e^{-}+ H* \leftrightarrow H_{2} (Heyrovsky step)$$

$$H* + H* \leftrightarrow H_{2} (Tafel step)$$

Here the Volmer-Heyrovsky mechanism is used for microkinetic modeling.^7-9^ The rate constant was calculated by

$$\begin{aligned} k=\frac{k_{B}T}{h}\exp\left( -\frac{G_{a}}{k_{B}T} \right)\exp\left( -\frac{e\beta\eta}{k_{B}T} \right) \end{aligned}$$

where $G_{a}$ is the activation free energy, $k_{B}$ is the Boltzmann constant, $h$ is Planck’s constant, and $T$ is the reaction temperature (273K). $\beta$ is the symmetry factor and is equal to 0.5. $\eta$ is the overpotential.

The microkinetic modeling of the volcano plot was based on the steady-state approximation and simulated using Python code implemented in our *DigCat* platform.^10^ Rates for intermediate steps were calculated using the following equation:

$$\begin{aligned} rate=k_{f} \prod\theta_{\mathrm{reac}}-k_{r} \prod\theta_{\mathrm{prod}} \end{aligned}$$

where $\theta_{\mathrm{reac}}$ and $\theta_{\mathrm{prod}}$ are the coverage of reactants and products, respectively.

The intermediate three reactions considered in the modeling are shown in the following equations:

$$H^{+}+e^{-}\leftrightarrow H* \left( \mathrm{Volmer} \right)$$

$$H^{+}+e^{-}+H*\leftrightarrow H_{2} (Heyrovsky)$$

$$\mathrm{HO}^{-}\leftrightarrow HO*+e^{-} (HO*adsorption)$$

Three surface states are considered:

$$\theta_{*}+\theta_{H*}{+\theta}_{HO*}=1$$

The current density was then obtained by converting the reaction rates using the equation below:

$$TOF=k_{H}\theta_{H}$$

$$\begin{aligned} j=\rho*2e*TOF \end{aligned}$$

where $k_{H}$ represents the rate constant of the Heyrovsky step, $\theta_{H}$ represents the steady-state coverage of H*, $\rho$ is the surface density of active sites, $2e$ is the number of electrons for H_2_ production, and $\mathrm{TOF}$ is the turnover frequency of electrons. Note that the adsorption free energy is assumed to be a constant value during equation solving and does not change with coverage. The energy barrier of Heyrovsky step is derived from the scaling relationship between $\Delta G_{H*}$ and $G_{a}$. As the Volmer barriers are typically low,^11^ we mainly focus on the competitive adsorption of H* and HO* derived from HO^─^ and H^+^ in the electrolyte and its effects on the surface coverage and the Heyrovsky rate. The Heyrovsky barrier is described using a model benchmarked against CoPc experiments ($G_{a}=0.89\Delta G_{H*}+1.17$).^12^ H adsorption is a necessary process for HER, while the competitive adsorption of HO physically blocks active sites and hinders the HER. Our 2D HER microkinetic model explicitly accounts for the competitive electrochemical adsorption of HO* and H* (derived from HO⁻ and H⁺ in the electrolyte, respectively). According to the previous work,^12^ HO* indeed can inhibit HER activity, indicating that hydrogen binding energy is insufficient for describing HER activity. Therefore, based on the CHE model and the microkinetic model, we examined the effects of HO adsorption and desorption on the HER.

We took a mean-field approach to microkinetic modeling. In the novel 2D model, the change in HO coverage and its effect on the active site concentration can be considered during HER. Overall, the coverages of H* and HO* on CoP surfaces can be predicted at a constant potential method. Then, the HER activity can be accurately predicted using these two descriptors (∆*G*_H*_ and ∆*G*_HO*_), which has been validated by our previous experimental results.^12^

### Supplementary Text 8 Synthesis of CoP Nanosheets

A piece of carbon paper (2 × 2 cm^2^) was first immersed in dilute hydrochloric acid to remove surface oxides and impurities. Subsequently, it was ultrasonicated in acetone for 15 min and thoroughly rinsed with ethanol and deionized (DI) water several times. The Co(OH)_2_ nanosheets were electrodeposited onto the pretreated carbon paper using an electrolyte containing 0.03 M Co(NO_3_)_2_·6H_2_O and 0.05 M NaNO_3_. The electrodeposition process was carried out at a constant potential of -1.0 V (vs. RHE) for 360 s.

Subsequently, the deposited Co(OH)_2_ was carefully rinsed off from the carbon paper using DI water to collect the precursor powder. The collected product was then dried in a vacuum oven at 60 °C. For the phosphorization process, the as-obtained Co(OH)_2_ powder and 1 g of NaH_2_PO_2_·H_2_O were placed in two separate porcelain boats. The boat containing NaH_2_PO_2_·H_2_O was placed at the upstream side of the tube furnace, while the Co(OH)_2_ powder was placed at the downstream side, with a distance of approximately 10 cm between them. Prior to heating, the tube furnace was purged with Argon (Ar) for 15 min to eliminate air. The system was then heated to 300 °C at a heating rate of 1.5 °C min^-1^ and maintained for 120 min under an Ar atmosphere. Finally, the system was naturally cooled to room temperature under Ar protection to obtain the CoP nanosheet catalyst.

### Supplementary Text 9 Material Characterization

### The crystal structures of the as-prepared samples were analyzed by X-ray diffraction (XRD) using a Rigaku SmartLab diffractometer with Cu Kα radiation. The surface morphology and microstructure were characterized by field-emission scanning electron microscopy (SEM, Hitachi SU8600) and transmission electron microscopy (TEM, Thermo Fisher Scientific Talos). High-angle annular dark-field scanning transmission electron microscopy (HAADF-STEM) images and energy-dispersive X-ray spectroscopy (EDS) elemental mapping were acquired on the same Talos microscope to reveal the elemental distribution. The surface chemical compositions and valence states were investigated by X-ray photoelectron spectroscopy (XPS) using a Thermo Fisher Scientific K-Alpha spectrometer with an Al Kα X-ray source. To deeply probe the local coordination environment and electronic structure, synchrotron radiation experiments were conducted. The Co K-edge X-ray absorption fine structure (XAFS) measurements were performed at the BL14W1 beamline of the Shanghai Synchrotron Radiation Facility (SSRF, Shanghai, China). The storage ring was operated at an energy of 3.5 GeV with a maximum electron current of 260 mA. A double-crystal Si (111) monochromator was employed to monochromatize the X-ray beam. The Co K-edge spectra were collected in transmission mode at room temperature. A Co metal foil was measured simultaneously as a reference for energy calibration. The Co L-edge X-ray absorption near-edge structure (XANES) spectra were acquired at the BL12B-a beamline of the National Synchrotron Radiation Laboratory (NSRL, Hefei, China). The spectra were recorded in the total electron yield (TEY) mode by measuring the sample drain current. The energy scale was calibrated using the standard Co peaks. All the spectra were normalized to the maximum intensity of the main peak for comparison.

*In−situ* Raman spectroscopy was performed using a confocal Raman microscope (Renishaw inVia-Qontor) with a 50X (NA = 0.55) objective lens and a 532 nm laser. The laser intensity was set to be 10% and the signal acquisition time is 10 s for each Raman spectrum with the accumulation times of 1. The working electrode was obtained by spray-coating ink (composed of 1 mg of freshly CoP catalysts, 80 μL of isopropanol, and 20 µL of Nafion solution) onto the gas-diffusion layer. Electrochemical signals were controlled by a CHI760E electrochemical workstation using a Pt mesh as the counter electrode. To investigate the HER mechanism across a wide pH range, three electrolytes including acidic (0.5 M H_2_SO_4_), neutral (1.0 M PBS), and alkaline (1.0 M KOH) solutions were employed. An Ag/AgCl electrode was used as the reference electrode in acidic and neutral media while an Hg/HgO electrode was used in alkaline media. Potential-dependent Raman spectra were recorded under steady-state chronoamperometric conditions at various potentials ranging from -0.01 V to -0.40 V vs. RHE to monitor the structural evolution of the CoP surface and the vibrational signals related to P–O(H) species.

*In-situ* attenuated total reflectance-surface enhanced infrared absorption spectroscopy (ATR-SEIRAS) was performed on a Thermo Scientific Nicolet 6700 FTIR spectrometer equipped with a liquid nitrogen-cooled mercury cadmium telluride (MCT) detector. A germanium prism, with its reflecting plane chemically deposited with a thin Au film as the conductive substrate, was utilized as the working electrode within a custom electrochemical cell. To investigate the HER mechanisms and surface intermediates under different pH environments, three electrolytes including acidic (0.5 M H_2_SO_4_), neutral (1.0 M PBS), and alkaline (1.0 M KOH) solutions were employed. An Ag/AgCl electrode was used as the reference electrode in acidic and neutral media, while an Hg/HgO electrode was used in alkaline media, with a platinum wire serving as the counter electrode in all cases. All electrochemical sequences were controlled by a CHI760E electrochemical workstation. The spectral acquisition was conducted with a resolution of 4 cm^-1^ and each spectrum was obtained by averaging multiple scans for a total acquisition time of 30 s. Potential-dependent spectra were collected under steady-state conditions at various potentials from -0.01 V to -0.40 V vs. RHE to monitor the evolution of key intermediates, specifically the P–O stretching and P–H bending vibrations on the CoP surface. Additionally, the (1.0 M KOH + D_2_O) electrolyte was employed to further confirm the P–H vibration using isotopic labeling effect. All spectra are presented as absorbance after subtracting the background spectrum collected at the open circuit potential.

Thermogravimetric analysis (TGA) was performed using a HITACHI STA200 thermal analyzer. Prior to the measurements, the sample was purged with nitrogen gas for 2 hours to eliminate any surface-adsorbed impurities. Approximately 5 mg of the sample was placed into an alumina crucible and heated to 800 °C at a constant heating rate of 10 °C/min. A continuous nitrogen flow was maintained throughout the entire testing process.

The concentrations of dissolved Co and P elements in the electrolyte after the electrochemical tests were determined using an Inductively Coupled Plasma Mass Spectrometer (ICP-MS, Agilent 7700). During the measurement, the instrument was operated with an RF power of 1.55 kW and an RF matching of 1.80 V. The gas flow rates were configured as follows: auxiliary gas at 1.50 L·min^-1^, carrier gas at 0.71 L·min-1, and makeup gas at 0.48 L·min-1. The sample uptake time and stabilization time were set to 45 s and 30 s, respectively, with an integration time of 0.90 s per mass. All liquid samples were appropriately diluted (10-fold dilution for specific samples) prior to the measurements to ensure the analyte concentrations fell within the calibration range.

**Supplementary Figure**

### Supplementary Fig. 1. Bulk Pourbaix of CoP with the pH and applied potential (vs. SHE) as the input. This bulk Pourbaix diagram of CoP suggests that it is high unstable under the conditions of HER.

### Supplementary Fig. 2. Summary of the reported electrocatalytic HER performance of CoP-based catalysts in 0.5 M H_2_SO_4_, 1.0 M PBS, and 1.0 M KOH. (A) Overpotentials (η) required to reach a current density of 10 mA·cm^-2^ and (B) corresponding Tafel slopes. (C) *iR*-corrected overpotentials (η) at 10 mA·cm^-2^ and (D) *iR*-corrected Tafel slopes. Noted that it is acquired by large-scale data mining and analyzed with the aid of the *DigCat* platform (https://www.digcat.org/).

### Supplementary Fig. 3. Statistics of (A) overpotentials (η) at the standard current density of 10 mA·cm^-2^ and (B) Tafel slopes for CoP-based HER materials.

### Supplementary Fig. 4. (1×1) CoP surface supercells separated by a 20 Å vacuum layer, constructed from the optimized orthorhombic CoP unit cell for seven-layer slab calculations of surface energies. Side and top views are shown in the upper and lower panels, respectively. The “sym” and “asym” refer to the symmetrical and asymmetrical CoP slab models, respectively.

### Supplementary Fig. 5. The surface energy for differently exposed facets of CoP. Surface energies of CoP follow the order: CoP(011) < CoP(111) < CoP(001) < CoP (010) < CoP(102) < CoP(201) < CoP(100). The “sym” and “asym” refer to the symmetrical and asymmetrical CoP slab models, respectively.

### Supplementary Fig. 6. Surface Pourbaix diagrams for (A) H*, (B) O*, (C) HO*, (D) P_V_*, and (E) H_2_O* species on CoP(011).

### Supplementary Fig. 7. Surface Pourbaix diagrams for (A) H*, (B) O*, (C) HO*, (D) P_V_*, and (E) H_2_O* species on CoP(111).

### Supplementary Fig. 8. Surface Pourbaix diagrams for (A) H*, (B) O*, (C) HO*, (D) P_V_*, and (E) H_2_O* species on CoP(001).

### Supplementary Fig. 9. Surface Pourbaix diagrams for (A) H*, (B) O*, (C) HO*, (D) P_V_*, and (E) H_2_O* species on CoP(010).

### Supplementary Fig. 10. AIMD simulations for (A) CoP(011), (B) CoP(111), (C) CoP(001), and (D) CoP(010) surfaces with the coverage of HO* under 500 K with the time step of 1.0 fs for a period of 100 ps.

### Supplementary Fig. 11. Optimized surface configurations after AIMD simulations for (A) CoP(011), (B) CoP(111), (C) CoP(001), and (D) CoP(010) surfaces with the coverage of HO* under 500 K with the time step of 1.0 fs for a period of 100 ps.

### Supplementary Fig. 12. AIMD simulations for (A) CoP(011), (B) CoP(111), (C) CoP(001), and (D–E) CoP(010) surfaces with the coverage of H* under 500 K with the time step of 1.0 fs for a period of 100 ps.

### Supplementary Fig. 13. Optimized surface configurations after AIMD simulations for (A) CoP(011), (B) CoP(111), (C) CoP(001), and (D–E) CoP(010) surfaces with the coverage of H* under 500 K with the time step of 1.0 fs for a period of 100 ps.

### Supplementary Fig. 14. AIMD simulations for H*-covered (A–C) and HO*-covered (D–F) CoP(011) surfaces with explicit H_2_O molecules under 500 K with the time step of 1.0 fs for a period of 100 ps. (A, D) Side and primary views of the optimized structures for the initial images. (B, E) Energy and temperature evolutions. (C, F) Side and primary views of the optimized structures for the final images.

### Supplementary Fig. 15. The effects of electrochemistry-induced HO* coverage on the *d*-DOS of surface Co and the average Bader charge for (A, E) CoP(011), (B, F) CoP(111), (C, G) CoP(001), and (D, H) CoP(010) surfaces.

**Supplementary Fig. 16.** Dissociation energy barriers of H_2_O for (**A**) CoP(011)-1/2ML-HO*, (**B**) CoP(111)-1/6ML-HO*, (**C**) CoP(001)-1/3ML-HO*, and (**D**) CoP(010)-1/2ML-HO* surfaces. The insets illustrate the corresponding top-view atomic configurations for the initial state (IS), transition state (TS), and final state (FS).

### Supplementary Fig. 17. Electrostatic potential of various CoP surfaces, including (A) CoP(011), (B) CoP(111), (C) CoP(001), and (D) CoP(010).

### Supplementary Fig. 18. A, Electric effect on the adsorption of H* on CoP(010)-1/2ML-H* with the fitted values for μ (dipole moment, e·Å) and α (polarizability, e^2^·V^-1^). B, ∆*G*_H*_ on CoP (010)-1/2ML-H* under the overpotential of 150 mV.

### Supplementary Fig. 19. A–C, Surface Pourbaix diagrams of H* and P_V_* for (A–B) CoP(101) and (C–D) CoP(211).

### Supplementary Fig. 20. Average formation energy of surface P_V_* under different concentrations. (A) CoP(011), (B) CoP(111), (C) CoP(001), and (D) CoP(010). The left panel is the corresponding CoP surfaces with P_V_*.

### Supplementary Fig. 21. The formation energy of P─1H*, P─2H*, and P─3H* groups over (A) CoP(011), (B) CoP(111), (C) CoP(001), and (D) CoP(010), respectively.

### Supplementary Fig. 22. The optimized configurations (top view) for P─1H*, P─2H*, and P─3H* groups over (A) CoP(011), (B) CoP(111), (C) CoP(001), and (D) CoP(010), respectively.

### Supplementary Fig. 23. The P migration barriers with H* adsorption on P sites of (A) CoP(011), (B) CoP(111), (C) CoP(001), and (D) CoP(010). The corresponding transition states, designated as TSn (n = 0, 1, 2, 3), are illustrated from a side perspective.

### Supplementary Fig. 24. Adsorption of H* and HO* on (A) Pt(110) and (B) Pt(111).

### Supplementary Fig. 25. (A–B) SEM and (C–D) HRTEM characterizations for as-synthesized CoP nanosheets.

**Supplementary Fig. 26. A**, Normalized Co K-edge XANES spectra of CoP and reference samples (Co foil, CoO, and CoOOH). **B**, The deduced valence state of the synthesized CoP with the reference samples. **C,** The Bader charge analysis of CoP based on DFT.

### Supplementary Fig. 27. A, The *k*^2^-weighted EXAFS oscillations in *k* space. B–F, Wavelet transform (WT) contour plots for the *k*^2^-weighted EXAFS signals of Co foil, CoPc, CoO, CoOOH, and Co_3_O_4_.

### Supplementary Fig. 28. Nyquist plots for CoP toward HER under the acid (0.5 M H_2_SO_4_), neutral (1.0 M PBS), and alkaline (1.0 M KOH) environments, respectively.

### Supplementary Fig. 29. The stability of the current densities (10 and 100 mA·cm^-2^) at different overpotentials under the (A) acid (0.5 M H_2_SO_4_), (B) neutral (1.0 M PBS), and (C) alkaline (1.0 M KOH) environments, respectively.

### Supplementary Fig. 30. Post-HER structural and morphological analysis of CoP electrocatalysts (i.e., CoP-after samples). (A) XRD, (B) SEM, and (C) HRTEM characterization of crystal structure, morphology, and surface lattice. More post-HER characterization of CoP-after samples is shown in Supplementary Figs. 29 and 35.

### Supplementary Fig. 31. Post-HER structural analysis of CoP electrocatalysts (i.e., CoP-after samples). (A) Morphological analysis (SEM) and (B) surface lattice characterization (HRTEM).

### Supplementary Fig. 32. TGA analyses of CoP electrocatalysts (i.e., CoP-before vs. CoP-after samples).

### Supplementary Fig. 33. *In-situ* (A) Raman and (B–C) ATR-SEIRAS characterizations for the surface P–O(H) and P–H groups at different overpotentials under the neutral (1.0 M PBS) environment

### Supplementary Fig. 34. *In-situ* (A) Raman and (B–C) ATR-SEIRAS characterizations for the surface P–O(H) and P–H groups at different overpotentials under the alkaline (1.0 M KOH) environment.

### Supplementary Fig. 35. (A–B) *In-situ* ATR-SEIRAS characterizations for the surface P–D groups at different overpotentials under the alkaline (1.0 M KOH+D_2_O) environment.

### Supplementary Fig. 36. SEM images of CoP samples after immersion for 24 h in (A–C) acidic (0.5 M H_2_SO_4_), (D–F) neutral (1.0 M PBS), and (G–I) alkaline (1.0 M KOH) electrolytes for 24 h.

### Supplementary Fig. 37. SEM images of CoP samples after HER at the current density of 10 mA·cm^-2^ for 24 h in (A–C) acidic (0.5 M H_2_SO_4_), (D–F) neutral (1.0 M PBS), and (G–I) alkaline (1.0 M KOH) electrolytes for 24 h.

### Supplementary Fig. 38. Comparison of the (A) dissolved Co and (B) P concentrations determined by ICP–MS after a 24 h static immersion of CoP (named as “Electrolyte-1”) and after 24 h of HER electrocatalysis at 10 mA·cm^-2^ (named as “Electrolyte-2”).

### Supplementary Fig. 39. The stability of the high current densities (>1 A·cm^-2^) at different overpotentials under the (A) acid (0.5 M H_2_SO_4_), (B) neutral (1.0 M PBS), and (C) alkaline (1.0 M KOH) environment, respectively.

### Supplementary Fig. 40. CV curves of the (A–C) as-synthesized CoP sample and (E–G) CoP sample after testing at the high current density (>1 A·cm^-2^), measured at different scan rates under the (A, E) acid (0.5 M H_2_SO_4_), (B, F) neutral (1.0 M PBS), and (C, G) alkaline (1.0 M KOH) environment. (D, H) The plots of current density vs. scan rate of the as-synthesized CoP sample and CoP sample after testing at the high current density (>1 A·cm^-2^), respectively.

### Supplementary Fig. 41. Polarization curves (LSV) of the as-synthesized CoP sample and CoP sample after testing at the high current density (>1 A·cm^-2^) in (A) acidic (0.5 M H_2_SO_4_), (B) neutral (1.0 M PBS), and (C) alkaline (1.0 M KOH) electrolytes, respectively. Noticed that the current density is normalized by the ECSA.

### Supplementary Table

### Supplementary Table 1. Large-scale data mining for CoP-based materials towards electrocatalytic HER with the CoP as the main active phase under different electrolytes (All the data are also available in the *DigCat* database: <https://www.digcat.org/>).

| **Numbers** | **Materials** | **Electrolytes** | **Overpotential (@ 10 mA·cm^−2^)** | **Tafel slope** | **References** |
| --- | --- | --- | --- | --- | --- |
| 1 | N-Mo_x_C/CoP-0.5*^iR^* | 1.0 M KOH | 106 mV | 59 mV·dec^−1^ | Yu et al., *Nanotechnology*, **2024**, 35, 065704. |
| 2 | Co(OH)_2_/CoP | 1.0 M KOH | 20 mV | 109.8 mV·dec^−1^ | Xu et al., *Chem. Eng. J.*, **2024**, 490, 151697. |
| 3 | CoP/Ni_2_P*^iR^* | 1.0 M KOH | 67.4 mV | 73.9 mV·dec^−1^ | Wang et al., *Chem. Eng. Sci.*, **2025**, 301, 120740. |
| 4 | CoP/N-GO | 1.0 M KOH | 100 mV | 88 mV·dec^−1^ | Yu et al., *Materials Lett.*, **2025**, 382, 137924. |
| 5 | CoP-NC/PET*^iR^* | 1.0 M KOH | 63 mV | 48.09 mV·dec^−1^ | Sun et al., *Int. J. Hydrogen Energy*, **2024**, 63, 28. |
| 6 | CoP/FeP*^iR^* | 1.0 M KOH | 190 mV | 73 mV·dec^−1^ | Zhang et al., *J. Colloid Interf. Sci.*, **2025**, 679, 20. |
| 7 | Co/CoP@NC*^iR^* | 1.0 M KOH | 142 mV | 67 mV·dec^−1^ | Liao et al., *Int. J. Hydrogen Energy*, **2024**, 81, 624. |
| 8 | CoTe-CoP/NF*^iR^* | 0.5 M H_2_SO_4_ | 51 mV | 52.8 mV·dec^−1^ | Zhu et al., *Sci. China Mater.*, **2024**, 67, 1891. |
|  |  | 1.0 M KOH | 53 mV | 57.8 mV·dec^−1^ |  |
|  |  | 1.0 M PBS | 75 mV | 75.7 mV·dec^−1^ |  |
| 9 | Mo-CoP*^iR^* | 0.5 M H_2_SO_4_ | 78.7 mV | 55.7 mV·dec^−1^ | Zhao et al., *ACS Appl. Nano Mater.*, **2024**, 7, 17868. |
|  |  | 1.0 M KOH | 88.4 mV | 87.2 mV·dec^−1^ |  |
|  |  | 1.0 M PBS | 112.6 mV | 120.3 mV·dec^−1^ |  |
| 10 | CoP/FeP*^iR^* | 0.5 M H_2_SO_4_ | 98 mV | 53 mV·dec^−1^ | Adam et al., *ACS Appl. Mater. Interfaces*, **2024**, 16, 55218. |
| 11 | CoP*^iR^* |  | 265 mV | 95 mV·dec^−1^ |  |
| 12 | I_SA_-CoP_n_/CoP*^iR^* | 0.5 M H_2_SO_4_ | 44 mV | 41.2 mV·dec^−1^ | Wang et al., *Small*, **2024**, 20, 2403170. |
| 13 | Br-CoP*^iR^* | 1.0 M KOH | 76 mV | 57.9 mV·dec^−1^ | Yao et al., *Energy Environ. Mater.*, **2025**, 0, e70013. |
| 14 | Fe-CoP NFs | 1.0 M KOH | 122 mV | 66.2 mV·dec^−1^ | Yuan et al., *Energy Environ. Mater.*, **2024**, 7, e12747. |
| 15 | Cr-CoP/Fe_2_P*^iR^* | 1.0 M KOH | 114 mV | 105.7 mV·dec^−1^ | Yan et al., *J. Electrochem. Soc.*, **2024**, 171, 056507. |
| 16 | CoP/MoS_2_*^iR^* | 1.0 M KOH | 88 mV | 87.5 mV·dec^−1^ | Zhang et al., *RSC Adv.*, **2024**, 14, 19294. |
| 17 | Fe-CoP nanoFC | 1.0 M KOH | 203 mV | 120.23 mV·dec^−1^ | Yuan et al., *Int. J. Hydrogen Energy*, **2024**, 90, 1401. |
| 18 | Co(002)/CoP*^iR^* | 1.0 M KOH | 85 mV | 110 mV·dec^−1^ | Wang et al., *Int. J. Hydrogen Energy*, **2024**, 67, 1106. |
| 19 | VO_x_-CoP/NF*^iR^* | 1.0 M KOH | 65 mV | 46.8 mV·dec^−1^ | Li et al., *Nano Energy*, **2024**, 126, 109613. |
| 20 | Pd-CoP@NC*^iR^* | 0.5 M H_2_SO_4_ | 37.5 mV | 56 mV·dec^−1^ | Wang et al., *Int. J. Hydrogen Energy*, **2024**, 82, 759. |
| 21 | Mo-CoP@C*^iR^* | 1.0 M KOH | 122 mV | 68.99 mV·dec^−1^ | Luo et al., *Chem. Eng. J.*, **2025**, 510, 161519. |
| 22 | Ru-CoP/Ni_2_P*^iR^* | 1.0 M KOH | 64 mV | 51.9 mV·dec^−1^ | Zhang et al., *Adv. Sci.*, **2024**, 11, 2401398. |
|  |  | 0.01 M PBS | 125 mV | 58.4 mV·dec^−1^ |  |
|  |  | 0.5 M H_2_SO_4_ | 53 mV | 35.9 mV·dec^−1^ |  |
| 23 | CoP/MoO_2_*^iR^* | 1.0 M KOH | 11 mV | 83.6 mV·dec^−1^ | Liu et al., *Inorg. Chem.*, **2024**, 63, 2803. |
| 24 | RuP_2_/CoP@NPC | 1.0 M KOH | 12 mV | 28.2 mV·dec^−1^ | Tian et al., *Int. J. Hydrogen Energy*, **2025**, 124, 1. |
|  |  | 0.5 M H_2_SO_4_ | 24 mV | 32.2 mV·dec^−1^ |  |
|  |  | 1.0 M PBS | 61 mV | 46.3 mV·dec^−1^ |  |
| 25 | CoO/CoP*^iR^* | 1.0 M KOH | 148 mV | 84 mV·dec^−1^ | Lin et al., *RSC Adv.*, **2024**, 14, 14886. |
| 26 | CoMoO_4_/CoP-10/CC*^iR^* | 1.0 M KOH | 127 mV | 69.9 mV·dec^−1^ | Yu et al., *Energy Fuels*, **2024**, 38, 11172. |
| 27 | YSCoO/CoP*^iR^* | 1.0 M KOH | 126 mV | 65 mV·dec^−1^ | Kim et al., *J. Mater. Chem. A*, **2025**, 13, 13763. |
| 28 | CoP/Ti_3_C_2_T_x_*^iR^* | 1.0 M KOH | 103 mV | 89 mV·dec^−1^ | Liu et al., *J. Colloid Interface Sci.*, **2024**, 661, 709. |
| 29 | Ru/N-CoP-Co_2_P*^iR^* | 0.5 M H_2_SO_4_ | 63 mV | 46 mV·dec^−1^ | Fu et al., *Inorg. Chem.*, **2024**, 63, 15477. |
|  |  | 1.0 M KOH | 100 mV | 100 mV·dec^−1^ |  |
| 30 | CeO_2_-CoP/TM*^iR^* | 1.0 M KOH | 41 mV | 48.0 mV·dec^−1^ | Zhao et al., *J. Energy Chem.*, **2024**, 95, 490. |
| 31 | CoP@NC*^iR^* | 1.0 M KOH | − | 65 mV·dec^−1^ | Song et al., *J. Alloys Compd.*, **2025**, 1018, 179248. |
| 32 | Fe-Co_2_P/CoP*^iR^* | 1.0 M KOH | 174 mV | 72 mV·dec^−1^ | Yu et al., *Appl. Surf. Sci.*, **2024**, 670, 160665. |
| 33 | FeP-CoP*^iR^* | 1.0 M KOH | 105 mV | 106.73 mV·dec^−1^ | Xu et al., *New J. Chem.*, **2025**, 49, 3387. |
| 34 | CoP/FeP*^iR^* | 1.0 M KOH | 118 mV | 78 mV·dec^−1^ | Zeng et al, *Chem. Eng. J.*, **2024**, 491, 152084. |
| 35 | CoP@WP_2_/NF*^iR^* | 0.5 M H_2_SO_4_ | 13 mV | 62 mV·dec^−1^ | Wei et al., *Small*, **2025**, 2412689. |
|  |  | 1.0 M KOH | 68 mV | 104 mV·dec^−1^ |  |
| 36 | CoP-CNS*^iR^* | 1.0 M KOH | 137 mV | 70 mV·dec^−1^ | Wang et al., *ACS Appl. Nano Mater.*, **2024**, 7, 12821. |
| 37 | H-Co_x_P*^iR^* | 0.5 M H_2_SO_4_ | 110 mV | 51 mV·dec^−1^ | Tian et al., *Sustain. Energy Fuels*, **2017**, 1, 62. |
| 38 | CoP_x_/N-rGO | 1.0 M KOH | 104 mV | 65 mV·dec^−1^ | Zheng et al., *Adv. Mater. Interfaces*, **2018**, 5, 1800515. |
| 39 | CoP_x_*^iR^* | 0.5 M H_2_SO_4_ | 128 mV | 78 mV·dec^−1^ | Ma et al., *Nano Res.*, **2019**, 12, 375. |
|  |  | 1.0 M KOH | 155 mV | 99 mV·dec^−1^ |  |
| 40 | CoP/CC*^NiR^* | 0.5 M H_2_SO_4_ | 67 mV | 51 mV·dec^−1^ | Tian et al., *J. Am. Chem. Soc.*, **2014**, 136, 7587. |
| 41 | CoP/ CNT*^NiR^* | 0.5 M H_2_SO_4_ | 122 mV | 54 mV·dec^−1^ | Liu et al., *Angew. Chem. Int. Ed.*, **2014**, 53, 6710. |
| 42 | CoP NFs*^NiR^* | 0.5 M H_2_SO_4_ | 120 mV | 54.8 mV·dec^−1^ | Ji et al., *ACS Catal.*, **2020**, 10, 1, 412. |
|  |  | 1.0 M KOH | 136 mV | 56.2 mV·dec^−1^ |  |
| 43 | CoP-InNC@CNT*^NiR^* | 0.5 M H_2_SO_4_ | 153 mV | 62.0 mV·dec^−1^ | Chai et al., *Adv. Sci.*, **2020**, 7, 1903195. |
|  |  | 1.0 M KOH | 159 mV | 56.0 mV·dec^−1^ |  |
| 44 | CoP/rGO@Ti*^iR^* | 1.0 M KOH | 103 mV | 66.1 mV·dec^−1^ | Xu et al., *Nat. Commun.*, **2023**, 14, 7708. |
| 45 | CoP catalyst ink*^iR^* | 1.0 M KOH | 203 mV | 67.1 mV·dec^−1^ |  |
| 46 | CoP/rGO*^NiR^* | 0.5 M H_2_SO_4_ | 105 mV | 50.0 mV·dec^−1^ | Jiao et al., *Chem. Sci.*, **2016**, 7, 1690. |
|  |  | 1.0 M KOH | 150 mV | 38.0 mV·dec^−1^ |  |
| 47 | CoP UPNSs*^iR^* | 0.5 M H_2_SO_4_ | 56 mV | 44.0 mV·dec^−1^ | Zhang et al., *Chem. Sci.*, **2017**, 8, 2769 |
| 48 | CoP nanoparticles*^iR^* | 0.5 M H_2_SO_4_ | 203 mV | 81.0 mV·dec^−1^ |  |
| 49 | PtSA-NT-NF*^iR^* | 1.0 M PBS | 24 mV | 30.0 mV·dec^−1^ | Zhang et al., *Angew. Chem. Int. Ed.*, **2017**, 56, 13694. |
| 50 | Cu_0.3_Co_2.7_P/NC*^NiR^* | 1.0 M KOH | 220 mV | 122 mV·dec^−1^ | Song et al., *Adv. Energy Mater.*, **2017**, 7, 1601555. |
| 51 | CoP/NCNHP*^iR^* | 0.5 M H_2_SO_4_ | 140 mV | 53 mV·dec^−1^ | Pan et al., *J. Am. Chem. Soc.*, **2018**, 140, 2610. |
|  |  | 1.0 M KOH | 115 mV | 66 mV·dec^−1^ |  |
| 52 | PANI/CoP HNWs-CFs*^NiR^* | 0.5 M H_2_SO_4_ | 50 mV | 34.5 mV·dec^−1^ | Feng et al., *J. Am. Chem. Soc.*, **2018**, 140, 5118. |
| 53 | CeO_x_/CoP@C*^iR^* | 1.0 M KOH | 127 mV | 56.7 mV·dec^−1^ | Lan et al., *J. Mater. Sci.*, **2018**, 53, 12123. |
| 54 | Co/CoP*^iR^* | 1.0 M KOH | 253 mV | 73.8 mV·dec^−1^ | Xue et al., *Adv. Energy Mater.*, **2017**, 7, 1602355. |
|  |  | 1.0 M PBS | 138 mV | − |  |
|  |  | 0.5 M H_2_SO_4_ | 178 mV | − |  |
| 55 | CoP NTs*^iR^* | 0.5 M H_2_SO_4_ | 108 mV | 59.0 mV·dec^−1^ | Zhang et al., *Mater. Lett.*, **2017**, 202, 146. |
| 56 | CoP@NPCP | 1.0 M KOH | 150 mV | 20 mV·dec^−1^ | Li et al., *Carbon*, **2019**, 150, 446. |
| 57 | CoS_2_/CoP/CC | 1.0 M KOH | 90 mV | 91.2 mV·dec^−1^ | Pan et al., *Electrocatal.*, **2019**, 10, 253. |
| 58 | CoP/CoMoO_4_-CC-2*^iR^* | 1.0 M KOH | 86 mV | 71 mV·dec^−1^ | Ma et al., *J. Colloid Interface Sci.*, **2024**, 669, 43. |
| 59 | Co_2_P/CoP@NF*^iR^* | 1.0 M KOH | 61 mV | 55.1 mV·dec^−1^ | Huang et al., *J. Catal.*, **2020**, 390, 23. |
| 60 | CoP/NC*^iR^* | 0.5 M H_2_SO_4_ | 139 mV | 59 mV·dec^−1^ | Zhou et al., *Int. J. Electrochem. Sci.*, **2022**, 17, 220666. |
|  |  | 1.0 M KOH | 248 mV | 82 mV·dec^−1^ |  |
| 61 | CoP PNWs*^NiR^* | 1.0 M KOH | 147 mV | 69 mV·dec^−1^ | Zhang et al., *Int. J. Hydrogen Energy*, **2017**, 42, 29080. |
| 62 | CoP-0.5*^iR^* | 1.0 M KOH | 91.7 mV | 183.5 mV·dec^−1^ | Duan et al., *Catal. Commun.*, **2022**, 162, 106379. |
| 63 | CoP-0.75*^iR^* |  | 87.4 mV | 197.3 mV·dec^−1^ |  |
| 64 | CoP-1.0*^iR^* |  | 73.4 mV | 166.54 mV·dec^−1^ |  |
| 65 | CoP-1.5*^iR^* |  | 57.7 mV | 58.3 mV·dec^−1^ |  |
| 66 | Co/CoP-HNC*^iR^* | 1.0 M KOH | 180 mV | 105.6 mV·dec^−1^ | Hao et al., *Mater. Horiz.*, **2018**, 5, 108. |
| 67 | Co/CoP-NC*^iR^* |  | 260 mV | 104.4 mV·dec^−1^ |  |
| 68 | H-Ni_0.04_-CoP@HC*^iR^* | 1.0 M KOH | 150 mV | 93 mV·dec^−1^ | Zhang et al., *J. Mater. Sci.*, **2022**, 57, 14430. |
| 69 | CoP@C*^iR^* |  | 244 mV | 170 mV·dec^−1^ |  |
| 70 | CoP/N-CNT/CC*^iR^* | 1.0 M KOH | 41 mV | 63 mV·dec^−1^ | Cai et al., *Chem. Eng. J.*, **2022**, 430, 132824. |
| 71 | CoP/CC*^iR^* |  | 100 mV | 69 mV·dec^−1^ |  |
| 72 | CoP/rGO/NF-3 | 1.0 M KOH | − | 135 mV·dec^−1^ | Yang et al., *Int. J. Hydrogen Energy*, **2021**, 46, 9690. |
| 73 | CoP/NF-300 |  | − | 173 mV·dec^−1^ |  |
| 74 | CoP/TM*^iR^* | 1.0 M KOH | 72 mV | 65 mV·dec^−1^ | Yang et al., *Nanotechnol.*, **2016**, 27, 23LT01. |
| 75 | CoP hollow polyhedron*^NiR^* | 0.5 M H_2_SO_4_ | 159 mV | 59 mV·dec^−1^ | Liu et al., *ACS Appl. Mater. Interfaces*, **2016**, 8, 2158. |
| 76 | Co_2_P/CoP*^iR^* | 1.0 M KOH | 196 mV | 53 mV·dec^−1^ | Zeng et al., *Mater. Chem. Phys.*, **2022**, 277, 125419. |
| 77 | CoP/WS_2_-2 | 0.5 M H_2_SO_4_ | 81 mV | 62 mV·dec^−1^ | Chen et al., *Appl. Surf. Sci.*, **2018**, 442, 352. |
| 78 | Co/CoP@HOMC | 1.0 M KOH | 120 mV | 78 mV·dec^−1^ | Li et al., *Adv. Energy Mater.*, **2021**, 11, 2102134. |
| 79 | CoP@HOMC |  | 150 mV | 95 mV·dec^−1^ |  |
| 80 | CoP@NC-350*^iR^* | 1.0 M KOH | 75 mV | 52 mV·dec^−1^ | Li et al., *Int. J. Hydrogen Energy*, **2021**, 46, 2095. |
| 81 | Amorphous WO_3_/crystalline CoP | 0.5 M H_2_SO_4_ | 69 mV | 57.2 mV·dec^−1^ | Liu et al., *ACS Appl. Energy Mater.*, **2022**, 5, 10794. |
|  |  | 1.0 M KOH | 49 mV | 59.7 mV·dec^−1^ |  |
| 82 | CoTe_2_/CoP | 1.0 M KOH | 80 mV | 57 mV·dec^−1^ | Yang et al., *Appl. Catal. B Environ.*, **2023**, 329, 122551. |
| 83 | CoP |  | 112 mV | 64 mV·dec^−1^ |  |
| 84 | CoP/C-3 | 1.0 M KOH | 109 mV | 76.6 mV·dec^−1^ | Zheng et al., *Appl. Surf. Sci.*, **2020**, 506, 144977. |
| 85 | CoP@NPMG*^iR^* | 1.0 M KOH | 151 mV | 75 mV·dec^−1^ | Liu et al., *Nanoscale*, **2018**, 10, 2603. |
|  |  | 0.5 M H_2_SO_4_ | 91 mV | 58 mV·dec^−1^ |  |
| 86 | Mo-CoP*^iR^* | 1.0 M KOH | 40 mV | 65 mV·dec^−1^ | Guan et al., *Nano Energy*, **2018**, 48, 73. |
| 87 | CoP*^iR^* |  | 160 mV | 75 mV·dec^−1^ |  |
| 88 | Amorphous CoP*^iR^* | 1.0 M KOH | 143 mV | 63 mV·dec^−1^ | Beltrán-Suito et al., *J. Mater. Chem. A*, **2019**, 7, 15749. |
| 89 | Crystalline CoP*^iR^* |  | 261 mV | 80 mV·dec^−1^ |  |
| 90 | CoP nanoparticles*^iR^* | 0.5 M H_2_SO_4_ | − | 50 mV·dec^−1^ | Popczun et al., *Angew. Chem. Int. Ed.*, **2014**, 53, 5427. |
| 91 | CoP@MoS_2_ | 0.5 M H_2_SO_4_ | 201 mV | 70.4 mV·dec^−1^ | Chen et al., *ChemistrySelect*, **2020**, 5, 3130. |
| 92 | Ru-CoP/NCs*^iR^* | 1.0 M KOH | 22 mV | 50 mV·dec^−1^ | Hao et al., *ACS Appl. Mater. Interfaces*, **2021**, 13, 56035. |
|  |  | 0.5 M H_2_SO_4_ | 98 mV | 56 mV·dec^−1^ |  |
|  |  | 1.0 M PBS | 106 mV | 84 mV·dec^−1^ |  |
| 93 | CoP/NC*^iR^* | 1.0 M KOH | 197 mV | 71 mV·dec^−1^ |  |
|  |  | 0.5 M H_2_SO_4_ | 174 mV | 122 mV·dec^−1^ |  |
|  |  | 1.0 M PBS | 257 mV | 125 mV·dec^−1^ |  |
| 94 | Urchin-like CoP*^iR^* | 0.5 M H_2_SO_4_ | 130 mV | 52 mV·dec^−1^ | Miao et al., *Int. J. Energy Res.*, **2021**, 45, 4735. |
| 95 | NiCoP-CoP/NF*^NiR^* | 1.0 M KOH | 73 mV | 91.3 mV·dec^−1^ | Liu et al., *ACS Appl. Mater. Interfaces*, **2019**, 11, 15528. |
| 96 | CoP/NF*^NiR^* |  | 108 mV | 102.2 mV·dec^−1^ |  |
| 97 | CoP/GF*^iR^* | 1.0 M KOH | − | 80.1 mV·dec^−1^ | Ren et al., *Int. J. Hydrogen Energy*, **2022**, 47, 3580. |
| 98 | CoP@CoMoO_4_ HNTs*^iR^* | 1.0 M KOH | 34 mV | 147 mV·dec^−1^ | Gao et al., *Int. J. Hydrogen Energy*, **2024**, 49, 260. |
| 99 | CC@N-CoP*^iR^* | 0.5 M H_2_SO_4_ | 42 mV | 41.2 mV·dec^−1^ | Zhou et al., *Adv. Mater.*, **2018**, 30, 1800140. |
| 100 | CC@CoP*^iR^* |  | 85 mV | 50.5 mV·dec^−1^ |  |
| 101 | Nb-CoP-0.05 NWs | 1.0 M KOH | 99 mV | 59.4 mV·dec^−1^ | Zou et al., *Int. J. Hydrogen Energy*, **2022**, 47, 13251. |
| 102 | V-CoP/CC*^iR^* | 0.5 M H_2_SO_4_ | 88 mV | 63 mV·dec^−1^ | Hua et al., *Front. Chem.*, **2020**, 8, 608133. |
|  |  | 1.0 M KOH | 98 mV | 64 mV·dec^−1^ |  |
| 103 | CoP/CC*^iR^* | 0.5 M H_2_SO_4_ | 111 mV | 62 mV·dec^−1^ |  |
|  |  | 1.0 M KOH | 124 mV | 76 mV·dec^−1^ |  |
| 104 | CoP-NCDs/NF*^NiR^* | 1.0 M KOH | 103 mV | 92 mV·dec^−1^ | Liu et al., *Carbon*, **2021**, 182, 327. |
| 105 | CoP/NF*^NiR^* |  | 151 mV | 125 mV·dec^−1^ |  |
| 106 | CoP NTs*^iR^* | 0.5 M H_2_SO_4_ | 129 mV | 60 mV·dec^−1^ | Du et al., *J. Mater. Chem. A*, **2014**, 2, 14812. |
| 107 | CoP NPs*^iR^* |  | 297 mV | 82 mV·dec^−1^ |  |
| 108 | Porous CoP/CePO_4_*^NiR^* | 1.0 M KOH | 162 mV | 48.7 mV·dec^−1^ | Wang et al., *ChemElectroChem*, **2021**, 8, 3762. |
| 109 | CoP*^NiR^* |  | 244 mV | 54.2 mV·dec^−1^ |  |
| 110 | Ce_0.5_-CoP*^iR^* | 1.0 M KOH | 162 mV | 83 mV·dec^−1^ | Li et al., *ACS Sustainable Chem. Eng.*, **2020**, 8, 10009. |
| 111 | Ce_1_-CoP^iR^ |  | 144 mV | 70 mV·dec^−1^ |  |
| 112 | Ce_2_-CoP*^iR^* |  | 155 mV | 82 mV·dec^−1^ |  |
| 113 | CoP*^iR^* |  | 166 mV | 85 mV·dec^−1^ |  |
| 114 | CoP-NC*^NiR^* | 0.5 M H_2_SO_4_ | 145 mV | 55 mV·dec^−1^ | Lai et al., *Electrochim. Acta*, **2021**, 375, 137966. |
|  |  | 1.0 M KOH | 167 mV | 57 mV·dec^−1^ |  |
|  |  | 1.0 M PBS | 252 mV | 110 mV·dec^−1^ |  |
| 115 | N–C@CoP/Ni_2_P*^iR^* | 0.5 M H_2_SO_4_ | 153 mV | 53.01 mV·dec^−1^ | Feng et al., *Int. J. Hydrogen Energy*, **2021**, 46, 8431. |
|  |  | 1.0 M KOH | 176 mV | 85.65 mV·dec^−1^ |  |
| 116 | CoP-N-C composite | 0.5 M H_2_SO_4_ | 91 mV | 42 mV·dec^−1^ | Zhang et al., *ChemCatChem*, **2015**, 7, 1920. |
| 117 | W-CoP*^iR^* | 0.5 M H_2_SO_4_ | 48 mV | 56 mV·dec^−1^ | Wu et al., *ACS Sustainable Chem. Eng.*, **2020**, 8, 14825. |
|  |  | 1.0 M KOH | 40 mV | 47 mV·dec^−1^ |  |
|  |  | 1.0 M PBS | 36 mV | 81 mV·dec^−1^ |  |
| 118 | Pt/d-CoP/NPC*^iR^* | 0.5 M H_2_SO_4_ | 6 mV | 12 mV·dec^−1^ | Wu et al., *Chin. J. Catal.*, **2023**, 46, 36. |
|  |  | 1.0 M KOH | 33 mV | 24 mV·dec^−1^ |  |
|  |  | 1.0 M PBS | 70 mV | 36 mV·dec^−1^ |  |
| 119 | Flower-like CoP/C nanosheets | 0.5 M H_2_SO_4_ | 151 mV | 128 mV·dec^−1^ | Wang et al., *Electrochim. Acta*, **2018**, 259, 822. |
| 120 | CoP nanosheets |  | 286 mV | 186 mV·dec^−1^ |  |
| 121 | Defect-rich porous CoP nanosheets*^iR^* | 0.5 M H_2_SO_4_ | 79.5 mV | 70.9 mV·dec^−1^ | Guo et al., *Int. J. Hydrogen Energy*, **2017**, 42, 26995. |
|  |  | 1.0 M KOH | 86.6 mV | − |  |
| 122 | CoP/Co_2_P*^iR^* | 0.5 M H_2_SO_4_ | 87 mV | 58 mV·dec^−1^ | Liu et al., *J. Power Sources*, **2021**, 486, 229351. |
|  |  | 1.0 M KOH | 133 mV | 60 mV·dec^−1^ |  |
| 123 | CoP/VGNHs hybrid | 0.5 M H_2_SO_4_ | 51 mV | 36 mV·dec^−1^ | Truong et al., *ACS Sustainable Chem. Eng.*, **2019**, 7, 4625. |
|  |  | 1.0 M KOH | 93 mV | 55 mV·dec^−1^ |  |
| 124 | CoP@Mo-Co-O*^iR^* | 1.0 M KOH | 62 mV | 60 mV·dec^−1^ | Zhou et al., *Chin. J. Catal.*, **2020**, 41, 691. |
| 125 | CoP*^iR^* |  | 117 mV | 76 mV·dec^−1^ |  |
| 126 | CoP@C nanocables | 0.5 M H_2_SO_4_ | 170 mV | 61 mV·dec^−1^ | Wang et al., *J. Power Sources*, **2015**, 286, 464. |
| 127 | Pi-CoP*^iR^* | 1.0 M KOH | 44 mV | 49 mV·dec^−1^ | Zhu et al., *Chem. Commun.*, **2020**, 56, 7159. |
| 128 | CoP/o-CC*^iR^* | 0.5 M H_2_SO_4_ | 118 mV | 79.77 mV·dec^−1^ | Wang et al., *Int. J. Hydrogen Energy*, **2022**, 47, 9209. |
|  |  | 1.0 M KOH | 95.45 mV | 86.4 mV·dec^−1^ |  |
| 129 | CoP/NiCoP NTs*^iR^* | 0.5 M H_2_SO_4_ | 125 mV | 71 mV·dec^−1^ | Lin et al., *Adv. Energy Mater.*, **2019**, 9, 1901213. |
|  |  | 1.0 M KOH | 133 mV | 88 mV·dec^−1^ |  |
| 130 | CoP-FeP*^iR^* | 0.5 M H_2_SO_4_ | 130.9 mV | 45.1 mV·dec^−1^ | Du et al., *Int. J. Hydrogen Energy*, **2019**, 44, 19978. |
|  |  | 1.0 M KOH | 163 mV | 50.1 mV·dec^−1^ |  |
|  |  | 1.0 M PBS | 134.2 mV | 51.2 mV·dec^−1^ |  |
| 131 | CoP@3D-NPC*^iR^* | 0.5 M H_2_SO_4_ | 127 mV | 58 mV·dec^−1^ | Yang et al., *Appl. Surf. Sci.*, **2019**, 476, 749. |
|  |  | 1.0 M KOH | 203 mV | 65 mV·dec^−1^ |  |
|  |  | 1.0 M PBS | 333 mV | 71 mV·dec^−1^ |  |
| 132 | CoP@NPC*^NiR^* | 0.5 M H_2_SO_4_ | 98 mV | 62.3 mV·dec^−1^ | Chen et al., *Int. J. Hydrogen Energy*, **2019**, 44, 24342. |
|  |  | 1.0 M KOH | 86 mV | 61.7 mV·dec^−1^ |  |
| 133 | CoP-CNTs*^NiR^* | 0.5 M H_2_SO_4_ | 139 mV | 52 mV·dec^−1^ | Wu et al., *Small*, **2017**, 13, 1602873. |
| 134 | MoS_2_\|\|CoP/NPC*^iR^* | 1.0 M KOH | 150 mV | 75.7 mV·dec^−1^ | Cai et al., *Int. J. Hydrogen Energy*, **2021**, 46, 34252. |
| 135 | CoP/CC | 0.5 M H_2_SO_4_ | 68 mV | 56 mV·dec^−1^ | Song et al., *Sci. China Mater.*, **2017**, 60, 1179. |
| 136 | CoP@Ni*^iR^* | 1.0 M KOH | 71 mV | 66 mV·dec^−1^ | Chen et al., *J. Colloid Interface Sci.*, **2023**, 637, 354. |
| 137 | CoP/Co_2_P-H*^NiR^* | 1.0 M KOH | 97 mV | 106.54 mV·dec^−1^ | Cao et al., *Small*, **2024**, 20, 2306113. |
| 138 | CoP*^NiR^* |  | 143 mV | 143.82 mV·dec^−1^ |  |
| 139 | CoP/Co_2_P-O*^NiR^* |  | 216 mV | 168.51 mV·dec^−1^ |  |
| 140 | Ni_2_P NS@CoP NW-CC*^iR^* | 0.5 M H_2_SO_4_ | 55 mV | 48 mV·dec^−1^ | Tang et al., *ACS Appl. Mater. Interfaces*, **2017**, 9, 41347. |
| 141 | CoP/MoO_2_*^iR^* | 0.5 M H_2_SO_4_ | 69 mV | 47.1 mV·dec^−1^ | Li et al., *Int. J. Hydrogen Energy*, **2021**, 46, 18353. |
|  |  | 1.0 M KOH | 78 mV | 48.1 mV·dec^−1^ |  |
|  |  | 1.0 M PBS | 165 mV | 102.9 mV·dec^−1^ |  |
| 142 | CoP@NF*^iR^* | 1.0 M KOH | 100 mV | 57 mV·dec^−1^ | Qiu et al., *ACS Sustainable Chem. Eng.*, **2019**, 7, 2360. |
| 143 | CoP-NCN | 0.5 M H_2_SO_4_ | 120 mV | 66 mV·dec^−1^ | Jia et al., *J. Colloid Interface Sci.*, **2021**, 601, 338. |
| 144 | Ru@CoP/CC*^iR^* | 0.5 M H_2_SO_4_ | 48 mV | 84 mV·dec^−1^ | Sun et al., *Int. J. Hydrogen Energy*, **2024**, 59, 1205. |
|  |  | 1.0 M KOH | 66 mV | 104 mV·dec^−1^ |  |
| 145 | Co/CoP-NF | 1.0 M NaOH | 35 mV | 71 mV·dec^−1^ | Bai et al., *ACS Appl. Mater. Interfaces*, **2016**, 8, 29400. |
| 146 | CoO/CoP heterostructure*^iR^* | 1.0 M KOH | − | 84 mV·dec^−1^ | Li et al., *Chin. Chem. Lett.*, **2021**, 32, 3355. |
| 147 | Ni_2_P–CoP HNSAs/CC | 0.5 M H_2_SO_4_ | 85 mV | 40 mV·dec^−1^ | Wang et al., *J. Mater. Chem. A*, **2016**, 4, 16992. |
|  |  | 1.0 M KOH | − | 120 mV·dec^−1^ |  |
| 148 | CoP@CoB*^iR^* | 1.0 M KOH | 135 mV | 80 mV·dec^−1^ | Lu et al., *J. Alloys Compd.*, **2022**, 920, 165883. |
| 149 | Porous CoP nanostructure*^iR^* | 0.5 M H_2_SO_4_ | 126 mV | 64 mV·dec^−1^ | Gao et al., *Int. J. Hydrogen Energy*, **2018**, 43, 13904. |
| 150 | Porous CoP nanosheets*^NiR^* | 0.5 M H_2_SO_4_ | 60 mV | 54 mV·dec^−1^ | Li et al., *Int. J. Hydrogen Energy*, **2024**, 51, 1279. |
|  |  | 1.0 M KOH | 90 mV | 62.9 mV·dec^−1^ |  |
|  |  | 1.0 M PBS | 128 mV | 69.9 mV·dec^−1^ |  |
| 151 | Mn-Co-P/Ti*^NiR^* | 0.5 M H_2_SO_4_ | 49 mV | 55 mV·dec^−1^ | Liu et al., *ACS Catal.*, **2017**, 7, 98. |
|  |  | 1.0 M KOH | 76 mV | 52 mV·dec^−1^ |  |
|  |  | 1.0 M PBS | 86 mV | 82 mV·dec^−1^ |  |
| 152 | CoP@N-VGNH*^iR^* | 0.5 M H_2_SO_4_ | 30 mV | 27 mV·dec^−1^ | Roy et al., *ACS Appl. Energy Mater.*, **2021**, 4, 5697. |
|  |  | 1.0 M KOH | 87 mV | 45 mV·dec^−1^ |  |
| 153 | NiCoP/CoP–Ti_4_O_7_ | 0.5 M H_2_SO_4_ | 128 mV | 65.5 mV·dec^−1^ | Ma et al., *ACS Sustainable Chem. Eng.*, **2018**, 6, 14275. |
| 154 | F-CoP NAs/CF | 1.0 M KOH | 35 mV | 32 mV·dec^−1^ | Sun et al., *Nano Res.*, **2023**, 16, 228. |
| 155 | F-CoP/CF-90 |  | 42 mV | 52 mV·dec^−1^ |  |
| 156 | CoP NAs/CF |  | 93 mV | 110 mV·dec^−1^ |  |
| 157 | Ga-CoP NSs/CFP*^iR^* | 1.0 M KOH | 44 mV | 62 mV·dec^−1^ | Zhang et al., *Chem. Eng. J.*, **2022**, 429, 132012. |
|  |  | 0.5 M H_2_SO_4_ | 82 mV | 49 mV·dec^−1^ |  |
| 158 | Se-CoP*^iR^* | 1.0 M KOH | 41 mV | 46 mV·dec^−1^ | Men et al., *Appl. Catal. B Environ.*, **2021**, 284, 119718. |
| 159 | CoP*^iR^* |  | 91 mV | 75 mV·dec^−1^ |  |
| 160 | CoP@CoO_x_*^iR^* | 1.0 M KOH | 35 mV | 44.9 mV·dec^−1^ | Fang et al., *ACS Appl. Energy Mater.*, **2020**, 3, 309. |
| 161 | Pristine CoP*^iR^* |  | 82 mV | 111.5 mV·dec^−1^ |  |
| 162 | V-CoP@a-CeO_2_*^iR^* | 1.0 M KOH | 68 mV | 48.1 mV·dec^−1^ | Yang et al., *Adv. Funct. Mater.*, **2020**, 30, 1909618. |
| 163 | V-CoP*^iR^* |  | 90 mV | 77.8 mV·dec^−1^ |  |
| 164 | CoP*^iR^* |  | 122 mV | 83.3 mV·dec^−1^ |  |
| 165 | N-C@CoP*^iR^* | 0.5 M H_2_SO_4_ | 35 mV | 50.8 mV·dec^−1^ | Cai et al., *Chem. Eng. J.*, **2022**, 446, 137132. |
| 166 | CoP/CSs-0.05*^iR^* | 0.5 M H_2_SO_4_ | 94 mV | 63 mV·dec^−1^ | Gao et al., *Sustain. Energy Fuels*, **2019**, 3, 823. |
| 167 | CoP/Co_3_S_4_@NSOC*^iR^* | 0.5 M H_2_SO_4_ | 132 mV | 72 mV·dec^−1^ | Jiang et al., *Int. J. Hydrogen Energy*, **2022**, 47, 28894. |
| 168 | Mg-doped CoP*^iR^* | 1.0 M KOH | 85 mV | 34.7 mV·dec^−1^ | Wang et al., *Int. J. Hydrogen Energy*, **2023**, 48, 35443. |
| 169 | CoP/MnO_x_ heterostructure*^iR^* | 1.0 M KOH | 135 mV | 59.4 mV·dec^−1^ | Yu et al., *Int. J. Hydrogen Energy*, **2023**, 48, 2593. |
| 170 | CoP*^iR^* |  | 184 mV | 68.6 mV·dec^−1^ |  |
| 171 | CoP(MoP)-CoMoO_3_@CN*^iR^* | 1.0 M KOH | 198 mV | 95 mV·dec^−1^ | Yu et al., *ACS Appl. Mater. Interfaces*, **2019**, 11, 6890. |
| 172 | CoP@N-doped Carbon | 0.5 M H_2_SO_4_ | 202 mV | 75.9 mV·dec^−1^ | Yang et al., *Chem. Asian J.*, **2023**, 18, e202300534. |
| 173 | TiO_2_-CoP/NF*^iR^* | 1.0 M KOH | 61 mV | 73 mV·dec^−1^ | Deng et al., *J. Energy Chem.*, **2022**, 74, 111. |
| 174 | CoP/NF*^iR^* |  | 107 mV | 99 mV·dec^−1^ |  |
| 175 | V-CoP/CC*^iR^* | 1.0 M KOH | 98 mV | 76 mV·dec^−1^ | Jia et al., *Nanoscale Adv.*, **2023**, 5, 4133. |
| 176 | CoP/CC*^iR^* |  | 149 mV | 105 mV·dec^−1^ |  |
| 177 | CoP | 0.5 M H_2_SO_4_ | 383 mV | 90 mV·dec^−1^ | Pan et al., *J. Mater. Chem. A*, **2016**, 4, 4745. |
| 178 | CoP/CNTs |  | 165 mV | 68 mV·dec^−1^ |  |
| 179 | CoP/NCNTs |  | − | 49 mV·dec^−1^ |  |
| 180 | Branched CoP nanostructures*^iR^* | 0.5 M H_2_SO_4_ | − | 48 mV·dec^−1^ | Popczun et al., *J. Mater. Chem. A*, **2015**, 3, 5420. |
| 181 | CoP/MoP@NPC | 0.5 M H_2_SO_4_ | 183 mV | 53.3 mV·dec^−1^ | Liu et al., *J. Colloid Interface Sci.*, **2023**, 631, 147. |
|  |  | 1.0 M KOH | 211 mV | 64.6 mV·dec^−1^ |  |
| 182 | CoP NDs*^iR^* | 0.5 M H_2_SO_4_ | 126 mV | 54.5 mV·dec^−1^ | Ji et al., *Sustain. Energy Fuels*, **2020**, 4, 1616. |
|  |  | 1.0 M KOH | 134 mV | 56.9 mV·dec^−1^ |  |
| 183 | CoP-O*^iR^* | 0.5 M H_2_SO_4_ | 91 mV | 54 mV·dec^−1^ | Kim et al., *Int. J. Energy Res.*, **2022**, 46, 13035. |
|  |  | 1.0 M KOH | 63 mV | 102 mV·dec^−1^ |  |
| 184 | CoP-CeO_2_/Ti*^iR^* | 1.0 M KOH | 43 mV | 45 mV·dec^−1^ | Zhang et al., *J. Mater. Chem. A*, **2018**, 6, 1985. |
| 185 | CoP/Ti*^iR^* |  | 70 mV | 75 mV·dec^−1^ |  |
| 186 | CoP/CN/Ni | 0.5 M H_2_SO_4_ | 66 mV | 39.5 mV·dec^−1^ | Chen et al., *Chem. Eng. J.*, **2021**, 415, 129031. |
|  |  | 1.0 M KOH | 106 mV | 53.4 mV·dec^−1^ |  |
| 187 | 3DOM Ru-CoP@NC*^iR^* | 0.5 M H_2_SO_4_ | 45 mV | 50 mV·dec^−1^ | Zhang et al., *Rare Met.*, **2024**, 43, 1095. |
|  |  | 1.0 M KOH | 15 mV | 38 mV·dec^−1^ |  |
| 188 | NiCoP-CoP/Ni/NF*^iR^* | 1.0 M KOH | 49 mV | 59.4 mV·dec^−1^ | Gao et al., *Int. J. Hydrogen Energy*, **2021**, 46, 23205. |
| 189 | NiCoP-CoP/NF*^iR^* |  | 76 mV | 64.0 mV·dec^−1^ |  |
| 190 | CoP/Ni_2_P@Co(OH)_2_*^iR^* | 0.5 M H_2_SO_4_ | 68 mV | 49 mV·dec^−1^ | Xing et al., *Mater. Today Energy*, **2022**, 30, 101142. |
|  |  | 1.0 M KOH | 39 mV | 55 mV·dec^−1^ |  |
| 191 | CoP/Ni_2_P*^iR^* | 0.5 M H_2_SO_4_ | 118 mV | 98 mV·dec^−1^ |  |
|  |  | 1.0 M KOH | 134 mV | 101 mV·dec^−1^ |  |
| 192 | F-CoP/Ni_2_P*^iR^* | 1.0 M KOH | 75 mV | 54.8 mV·dec^−1^ | Chen et al., *Int. J. Hydrogen Energy*, **2024**, 51, 1421. |
| 193 | CoP/RGO | 0.5 M H_2_SO_4_ | − | 104.8 mV·dec^−1^ | Ma et al., *J. Mater. Chem. A*, **2015**, 3, 5337. |
| 194 | CoP |  | − | 149.6 mV·dec^−1^ |  |
| 195 | Ni(OH)_2_@CoP/NF | 1.0 M KOH | 66 mV | 80.1 mV·dec^−1^ | Lyu et al., *Fuel*, **2025**, 386, 134285. |
| 196 | CoP/NF |  | 92 mV | 89.7 mV·dec^−1^ |  |
| 197 | Cr-CoP-NR/CC*^iR^* | 0.5 M H_2_SO_4_ | 38 mV | 44.8 mV·dec^−1^ | Zhang et al., *Small*, **2021**, 17, 2100832. |
| 198 | CoP/CC*^iR^* |  | 64 mV | 81.3 mV·dec^−1^ |  |
| 199 | CoP-HNCs | 0.5 M H_2_SO_4_ | 79 mV | 45.6 mV·dec^−1^ | Ganesan et al., *ChemElectroChem*, **2018**, 5, 1644. |
|  |  | 1.0 M KOH | 96 mV | 49.5 mV·dec^−1^ |  |
| 200 | Ti_2_NT_x_@MOF-CoP*^iR^* | 0.5 M H_2_SO_4_ | 129 mV | 96.7 mV·dec^−1^ | Zong et al., *Electrochim. Acta*, **2021**, 393, 139068. |
|  |  | 1.0 M PBS | 131 mV | 125.6 mV·dec^−1^ |  |
|  |  | 1.0 M KOH | 112 mV | 79.1 mV·dec^−1^ |  |
| 201 | Hierarchical CoP@Co-Fe LDH*^iR^* | 1.0 M KOH | 172 mV | 79 mV·dec^−1^ | Choi et al., *Int. J. Energy Res.*, **2022**, 46, 24633. |
| 202 | CoP@PNC@NF | 1.0 M KOH | 137.6 mV | 59.89 mV·dec^−1^ | Li et al., *Int. J. Hydrogen Energy*, **2023**, 48, 27215. |
| 203 | CoP@NF |  | 212.6 mV | 65.75 mV·dec^−1^ |  |
| 204 | CoP/CeO_2_-2.5*^NiR^* | 1.0 M KOH | 152 mV | 83.5 mV·dec^−1^ | Wang et al., *J. Alloys Compd.*, **2022**, 913, 165334. |
| 205 | CoP/CeO_2_-5*^NiR^* |  | 176 mV | 89.3 mV·dec^−1^ |  |
| 206 | CoP*^NiR^* |  | 187 mV | 96.8 mV·dec^−1^ |  |
| 207 | N-CoP/CeO_2_*^iR^* | 1.0 M KOH | 74 mV | 47 mV·dec^−1^ | Zhang et al., *Chem. Eng. J.*, **2023**, 460, 141119. |
| 208 | CoP/CeO_2_*^iR^* |  | 153 mV | 87 mV·dec^−1^ |  |
| 209 | N-CoP*^iR^* |  | 131 mV | 69 mV·dec^−1^ |  |
| 210 | MoS_2_-decorated hollow CoP | 0.5 M H_2_SO_4_ | 53 mV | 37 mV·dec^−1^ | Ge et al., *ACS Sustainable Chem. Eng.*, **2019**, 7, 10105. |
|  |  | 1.0 M KOH | 77 mV | 58 mV·dec^−1^ |  |
| 211 | CoP NWs*^NiR^* | 0.5 M H_2_SO_4_ | 110 mV | 54 mV·dec^−1^ | Jiang et al., *J. Mater. Chem. A*, **2014**, 2, 14634. |
| 212 | CoP NSs*^NiR^* |  | 164 mV | 61 mV·dec^−1^ |  |
| 213 | CoP NPs*^NiR^* |  | 221 mV | 87 mV·dec^−1^ |  |
| 214 | CoP@NCNWs/CF*^iR^* | 1.0 M KOH | 85 mV | 43.46 mV·dec^−1^ | Ning et al., *Inorg. Chem. Front.*, **2023**, 10, 2145. |
| 215 | CoP/CF*^iR^* |  | 112 mV | 175.2 mV·dec^−1^ |  |
| 216 | CoP-ML/NF*^iR^* | 1.0 M NaOH | 35.7 mV | 48.2 mV·dec^−1^ | Cao et al., *ChemSusChem*, **2021**, 14, 1094. |
| 217 | Au/CoP@NC-3*^iR^* | 0.5 M H_2_SO_4_ | 118.1 mV | 57.75 mV·dec^−1^ | Wang et al., *ACS Appl. Mater. Interfaces*, **2020**, 12, 16548. |
|  |  | 1.0 M KOH | 140.9 mV | 72.48 mV·dec^−1^ |  |
| 218 | CoP/C | 0.5 M H_2_SO_4_ | 154 mV | 68.6 mV·dec^−1^ | Wen et al., *Int. J. Hydrogen Energy*, **2021**, 46, 33420. |
| 219 | FeCoP/C |  | 97.5 mV | 43.8 mV·dec^−1^ |  |
| 220 | Fe-CoP HTPAs*^iR^* | 1.0 M KOH | 98 mV | 69 mV·dec^−1^ | Hu et al., *Small*, **2018**, 14, 1704233. |
| 221 | CoP TPAs*^iR^* |  | 120 mV | 90 mV·dec^−1^ |  |
| 222 | CoSe_2_-CoP | 0.5 M H_2_SO_4_ | 38 mV | 43 mV·dec^−1^ | Hussain et al., *Mater. Today Chem.*, **2024**, 35, 101844. |
|  |  | 1.0 M KOH | 51 mV | 52 mV·dec^−1^ |  |
| 223 | Petal-like CoO/CoP*^iR^* | 1.0 M KOH | 215 mV | 89.3 mV·dec^−1^ | Ma et al., *ChemistrySelect*, **2024**, 9, e202303891. |
| 224 | CoP@NC*^iR^* | 0.5 M H_2_SO_4_ | 78 mV | 49 mV·dec^−1^ | Yang et al., *ACS Catal.*, **2017**, 7, 3824. |
|  |  | 1.0 M KOH | 129 mV | 58 mV·dec^−1^ |  |
| 225 | CoP@C*^iR^* | 0.5 M H_2_SO_4_ | 130 mV | 64 mV·dec^−1^ |  |
|  |  | 1.0 M KOH | 170 mV | 83 mV·dec^−1^ |  |
| 226 | CoP*^iR^* | 0.5 M H_2_SO_4_ | 128 mV | 56 mV·dec^−1^ |  |
|  |  | 1.0 M KOH | 163 mV | 77 mV·dec^−1^ |  |
| 227 | Nanocrystalline CoP nanosheet assembly*^iR^* | 0.5 M H_2_SO_4_ | 49 mV | 30.1 mV·dec^−1^ | Yang et al., *Nano Energy*, **2015**, 15, 634. |
|  |  | 1.0 M KOH | − | 42.6 mV·dec^−1^ |  |
| 228 | CoFeP NS@Fe-CoP NW | 1.0 M KOH | 78 mV | 73 mV·dec^−1^ | Bi et al., *Appl. Phys. Lett.*, **2021**, 118, 093901. |
| 229 | CoP |  | 113 mV | 79 mV·dec^−1^ |  |
| 230 | Porous CoP/MoO_2_*^iR^* | 1.0 M KOH | 29 mV | 50 mV·dec^−1^ | Wang et al., *J. Mater. Chem. A*, **2020**, 8, 16018. |
| 231 | C-CoP-1/12*^NiR^* | 1.0 M KOH | 173 mV | 63.1 mV·dec^−1^ | Li et al., *Nanoscale*, **2019**, 11, 17084. |
| 232 | Cu_3_P-CoP/CC*^iR^* | 0.5 M H_2_SO_4_ | 59 mV | 58 mV·dec^−1^ | Du et al., *Chem. Commun.*, **2017**, 53, 12012. |
| 233 | CoP/CC*^iR^* |  | 83 mV | 89 mV·dec^−1^ |  |
| 234 | CoP nanostructures*^iR^* | 0.5 M H_2_SO_4_ | 68 mV | 68 mV·dec^−1^ | Yan et al., *J. Mater. Chem. A*, **2020**, 8, 7626. |
|  |  | 1.0 M PBS | 72 mV | 73 mV·dec^−1^ |  |
|  |  | 1.0 M KOH | 55 mV | 56 mV·dec^−1^ |  |
| 235 | NiSe@CoP NWs/NF*^NiR^* | 0.5 M H_2_SO_4_ | 73 mV | 41 mV·dec^−1^ | Xu et al., *Catal. Sci. Technol.*, **2018**, 8, 128. |
|  |  | 1.0 M KOH | 91 mV | 55 mV·dec^−1^ |  |
| 236 | CoP@DrGO*^iR^* | 1.0 M KOH | 36.6 mV | 43.1 mV·dec^−1^ | Chen et al., *Appl. Catal. B Environ.*, **2020**, 265, 118576. |
| 237 | FeP@CoP/CC*^NiR^* | 0.5 M H_2_SO_4_ | 40 mV | 34 mV·dec^−1^ | Zhang et al., *Nano Energy*, **2023**, 114, 108601. |
|  |  | 1.0 M KOH | 33 mV | 36 mV·dec^−1^ |  |
|  |  | 1.0 M PBS | 66 mV | 83 mV·dec^−1^ |  |
| 238 | Ru/CoP*^iR^* | 1.0 M KOH | − | 69 mV·dec^−1^ | Ji et al., *Adv. Mater.*, **2025**, 2503182. |
| 239 | NF/Ni_2_P/CoP-NS*^iR^* | 1.0 M KOH | 64 mV | 58.2 mV·dec^−1^ | Zhou et al., *Electrochim. Acta*, **2022**, 426, 140768. |
| 240 | NF/Ni_2_P/CoP-NN*^iR^* |  | 90 mV | 68.9 mV·dec^−1^ |  |
| 241 | Rh(OH)_3_/CoP*^iR^* | 1.0 M KOH | 13 mV | 24 mV·dec^−1^ | Xing et al., *Adv. Energy Mater.*, **2023**, 13, 2302376. |
|  |  | 0.5 M H_2_SO_4_ | 12 mV | 25 mV·dec^−1^ |  |
|  |  | 1.0 M PBS | 25 mV | 48 mV·dec^−1^ |  |
| 242 | Co_1-x_P/C | 1.0 M KOH | 65 mV | 38.6 mV·dec^−1^ | Wang et al., *J. Colloid Interface Sci.*, **2025**, 680, 214. |
| 243 | CoP HNS/CF*^iR^* | 0.5 M H_2_SO_4_ | 48 mV | 57 mV·dec^−1^ | Yoon et al., *Nano Res.*, **2020**, 13, 2469. |
|  |  | 1.0 M PBS | 120 mV | 83 mV·dec^−1^ |  |
|  |  | 1.0 M KOH | 55 mV | 50 mV·dec^−1^ |  |
| 244 | CoP-CoO/CC*^iR^* | 1.0 M KOH | 104 mV | 32.5 mV·dec^−1^ | Yao et al., *J. Colloid Interface Sci.*, **2022**, 607, 1343. |
| 245 | Mo-CoP*^iR^* | 1.0 M KOH | − | 69.1 mV·dec^−1^ | Li et al., *Ionics*, **2021**, 27, 3109. |
| 246 | Maize-like CoP nanorod arrays*^iR^* | 1.0 M KOH | 130 mV | 59 mV·dec^−1^ | Zheng et al., *Int. J. Hydrogen Energy*, **2021**, 46, 2026. |
| 247 | CoP/C | 0.5 M H_2_SO_4_ | 108 mV | 63 mV·dec^−1^ | Luo et al., *J. Energy Chem.*, **2017**, 26, 1147. |
|  |  | 1.0 M KOH | 163 mV | 101 mV·dec^−1^ |  |
| 248 | CoP/NPC/TF*^iR^* | 0.5 M H_2_SO_4_ | 91 mV | 54 mV·dec^−1^ | Huang et al., *Adv. Energy Mater.*, **2019**, 9, 1803970. |
|  |  | 1.0 M KOH | 80 mV | 50 mV·dec^−1^ |  |
| 249 | Pt-CoP*^iR^* | 0.5 M H_2_SO_4_ | 79 mV | − | Lei et al., *Adv. Sci.*, **2025**, 2504462. |
|  |  | 1.0 M PBS | 26.3 mV | − |  |
|  |  | 1.0 M KOH | 18 mV | − |  |
| 250 | Fe-CoP@Ni_2_P | 1.0 M KOH | 77.7 mV | 55.5 mV·dec^−1^ | Duan et al., *Nano Res.*, **2022**, 15, 8865. |
| 251 | Fe-CoP |  | 101 mV | 85.9 mV·dec^−1^ |  |
| 252 | CoP |  | 132 mV | 118.6 mV·dec^−1^ |  |
| 253 | Fe-CoB_i_/CoP/NF*^iR^* | 1.0 M KOH | − | 75.3 mV·dec^−1^ | Cao et al., *J. Colloid Interface Sci.*, **2023**, 652, 1703. |
| 254 | ip-CoP/CF*^iR^* | 0.5 M H_2_SO_4_ | 68 mV | 47 mV·dec^−1^ | Zhang et al., *Appl. Surf. Sci.*, **2019**, 481, 1524. |
|  |  | 1.0 M KOH | 76 mV | 93 mV·dec^−1^ |  |
| 255 | Fe-CoP/NF*^iR^* | 1.0 M KOH | 78 mV | 92 mV·dec^−1^ | Cao et al., *Adv. Sci.*, **2018**, 5, 1800949. |
| 256 | CoP/C-120*^iR^* | 0.5 M H_2_SO_4_ | 286 mV | 120 mV·dec^−1^ | Yuan et al., *ChemistrySelect*, **2021**, 6, 7068. |
| 257 | Mn-W-CoP/NF*^iR^* | 1.0 M KOH | 95.8 mV | 73.4 mV·dec^−1^ | Zhao et al., *Int. J. Hydrogen Energy*, **2024**, 51, 276. |
| 258 | CoP@a-CoO_x_*^iR^* | 1.0 M KOH | 132 mV | 89 mV·dec^−1^ | Yu et al., *Adv. Sci.*, **2018**, 5, 1800514. |
| 259 | CoP-Cu_3_P*^iR^* | 0.5 M H_2_SO_4_ | 48 mV | 45 mV·dec^−1^ | Du et al., *Mater. Today Commun.*, **2020**, 25, 101257. |
|  |  | 1.0 M KOH | 83 mV | 54 mV·dec^−1^ |  |
|  |  | 0.5 M PBS | 96 mV | 67 mV·dec^−1^ |  |
| 260 | Ru-CoP NAs | 1.0 M KOH | 52 mV | 39.7 mV·dec^−1^ | Liu et al., *J. Colloid Interface Sci.*, **2022**, 625, 457. |
| 261 | CoP/CoMoP_2_ | 1.0 M KOH | 93.6 mV | 89.9 mV·dec^−1^ | Zhang et al., *Nano Res.*, **2024**, 17, 3693. |
| 262 | Mo-CoP/Yb_2_O_3_*^iR^* | 1.0 M KOH | 33 mV | 44.04 mV·dec^−1^ | Fan et al., *ACS Appl. Energy Mater.*, **2024**, 7, 24, 12103. |
| 263 | La-CoP/Co(OH)_2_/NF | 1.0 M KOH | 114 mV | 99 mV·dec^−1^ | Yang et al., *New J. Chem.*, **2023**,47, 18818. |
| 264 | CoP/Ni_2_P@HPNCP*^iR^* | 1.0 M KOH | 106 mV | 61.89 mV·dec^−1^ | Zhang et al., *Nanoscale*, **2020**, 12, 23851. |
|  |  | 0.5 M H_2_SO_4_ | 130 mV | 64.91 mV·dec^−1^ |  |
|  |  | 1.0 M PBS | 141 mV | 85.92 mV·dec^−1^ |  |
| 265 | CoP-Cu_3_P/CC*^iR^* | 1.0 M KOH | 75 mV | − | Xie et al., *Energy Adv.*, **2023**, 2, 161. |
| 266 | NiP-CoP/NCS*^iR^* | 1.0 M KOH | 274.6 mV | 27.56 mV·dec^−1^ | Ipadeola et al., *Electrochim. Acta*, **2024**, 508, 145236. |
| 267 | Co/CoP@PNC-10*^iR^* | 1.0 M KOH | 137 mV | 89.8 mV·dec^−1^ | Gao et al., *Int. J. Hydrogen Energy*, **2024**, 51, 1333. |
| 268 | Co-MOF@CoP*^iR^* | 1.0 M KOH | 92.5 mV | 43.7 mV·dec^−1^ | Cui et al., *Int. J. Hydrogen Energy*, **2024**, 49, 1446. |
| 269 | V_5_-CoP-NC/NF*^iR^* | 1.0 M KOH | 119 mV | 57 mV·dec^−1^ | Liu et al., *Electrochim. Acta*, **2023**, 471, 143347. |
| 270 | CoP-2ph-CMP-800*^iR^* | 1.0 M KOH | 360 mV | − | Jia et al., *Chem. Commun.*, **2016**, 52, 13483. |
| 271 | CoP/WSe_2_ | 0.5 M H_2_SO_4_ | 163 mV | 76.5 mV·dec^−1^ | Qian et al., *Ind. Eng. Chem. Res.*, **2018**, 57, 483. |
| 272 | Cu-CoP NAs/CP*^iR^* | 1.0 M PBS | 81 mV | 83.5 mV·dec^−1^ | Yan et al., *Appl. Catal. B Environ.*, **2020**, 265, 118555. |
| 273 | N-CoO@CoP*^iR^* | 1.0 M KOH | − | 37 mV·dec^−1^ | Lu et al., *Electrochim. Acta*, **2020**, 330, 135210. |
| 274 | CoP NWs 40s/CC*^iR^* | 1.0 M KOH | − | 42.8 mV·dec^−1^ | Xu et al., *Adv. Mater.*, **2018**, 30, 1703322. |
| 275 | CoP NWs/CC*^iR^* |  | − | 126.8 mV·dec^−1^ |  |
| 276 | CeO_2_-CoP-C/CC*^iR^* | 0.5 M H_2_SO_4_ | 71 mV | 53 mV·dec^−1^ | Xiong et al., *Int. J. Hydrogen Energy*, **2018**, 43, 20372. |
| 277 | CoP-C/CC*^iR^* |  | 132 mV | 82 mV·dec^−1^ |  |
| 278 | CoP NS/CNTs*^NiR^* | 1.0 M KOH | 68 mV | 57 mV·dec^−1^ | Zhang et al., *Adv. Mater. Interfaces*, **2020**, 7, 1901302. |
| 279 | CoP/CoMoP^iR^ | 1.0 M KOH | 34 mV | 33 mV·dec^−1^ | Huang et al., *Nano Energy*, **2020**, 68, 104332. |
|  |  | 0.5 M H_2_SO_4_ | 72 mV | 50 mV·dec^−1^ |  |
| 280 | CoFeP/NC@CoP/Ni_2_P*^iR^* | 1.0 M KOH | 177 mV | 69 mV·dec^−1^ | Fu et al., *New J. Chem.*, **2023**, 47, 1226. |
| 281 | CoP/Ni_2_P*^iR^* |  | 221 mV | 98 mV·dec^−1^ |  |
| 282 | H-CoP@RGO | 0.5 M H_2_SO_4_ | 190 mV | 83 mV·dec^−1^ | Li et al., *Appl. Surf. Sci.*, **2020**, 506, 144975. |
| 283 | CoP@ZnFeP*^iR^* | 1.0 M KOH | 148 mV | 76 mV·dec^−1^ | Hu et al., *Chin. J. Catal.*, **2019**, 40, 1085. |
| 284 | CoFeP-30-30/NF*^iR^* | 1.0 M KOH | 29.8 mV | 68.4 mV·dec^−1^ | Yu et al., *Int. J. Hydrogen Energy*, **2023**, 48, 16715. |
| 285 | CoP/NF*^iR^* |  | 37.2 mV | 77.1 mV·dec^−1^ |  |
| 286 | RuP_4_@CoP/Ti*^iR^* | 0.5 M H_2_SO_4_ | 66.2 mV | 52.6 mV·dec^−1^ | Wang et al., *Appl. Surf. Sci.*, **2020**, 534, 147626. |
|  |  | 1.0 M KOH | 36 mV | 54.8 mV·dec^−1^ |  |
| 287 | CoP/CoS_2_/CC*^iR^* | 1.0 M KOH | 111.2 mV | 53.6 mV·dec^−1^ | Niu et al., *J. Colloid Interface Sci.*, **2023**, 652, 989. |
|  |  | 0.5 M H_2_SO_4_ | 58.1 mV | 42.5 mV·dec^−1^ |  |
|  |  | 1.0 M PBS | 128.9 mV | 73.1 mV·dec^−1^ |  |
| 288 | CoP/NCNs-3*^iR^* | 0.5 M H_2_SO_4_ | 98 mV | 54 mV·dec^−1^ | Li et al., *J. Colloid Interface Sci.*, **2021**, 600, 872. |
|  |  | 1.0 M KOH | 118 mV | 59 mV·dec^−1^ |  |
| 289 | CoP/NCNs-2*^iR^* | 0.5 M H_2_SO_4_ | 127 mV | 56 mV·dec^−1^ |  |
|  |  | 1.0 M KOH | 125 mV | 69 mV·dec^−1^ |  |
| 290 | CoP/NCNs-5*^iR^* | 0.5 M H_2_SO_4_ | 145 mV | 89 mV·dec^−1^ |  |
|  |  | 1.0 M KOH | 131 mV | 78 mV·dec^−1^ |  |
| 291 | CoP/C NPs*^iR^* | 0.5 M H_2_SO_4_ | 249 mV | 116 mV·dec^−1^ |  |
|  |  | 1.0 M KOH | 138 mV | 95 mV·dec^−1^ |  |
| 292 | CoP@a-CoB HNRA*^iR^* | 1.0 M KOH | 56.3 mV | 62.0 mV·dec^−1^ | Shi et al., *J. Mater. Chem. A*, **2021**, 9, 19719. |
|  |  | 0.5 M H_2_SO_4_ | 81.2 mV | 55.4 mV·dec^−1^ |  |
| 293 | NCCoP-ESi*^NiR^* | 0.5 M H_2_SO_4_ | 139 mV | 57 mV·dec^−1^ | Zhang et al., *Energy*, **2018**, 165, 537. |
|  |  | 1.0 M KOH | 203 mV | 45.4 mV·dec^−1^ |  |
| 294 | CoP-CNT composite microspheres*^iR^* | 0.5 M H_2_SO_4_ | 119 mV | 64 mV·dec^−1^ | Kim et al., *J. Alloys Compd.*, **2018**, 763, 652. |
| 295 | NC@Ru_SA_-CoP*^NiR^* | 1.0 M KOH | 15 mV | 48.1 mV·dec^−1^ | Wang et al., *Small*, **2023**, 19, 2301403. |
|  |  | 0.5 M H_2_SO_4_ | 13 mV | 35.6 mV·dec^−1^ |  |
| 296 | NiO_x_ NTAs/CoP NSs*^iR^* | 1.0 M KOH | 51 mV | 56 mV·dec^−1^ | Zai et al., *J. Colloid Interface Sci.*, **2023**, 643, 350. |
| 297 | CoP NSs*^iR^* |  | 123 mV | 79 mV·dec^−1^ |  |
| 298 | Tubular CoP*^iR^* | 0.5 M H_2_SO_4_ | 91 mV | 47.40 mV·dec^−1^ | Guo et al., *Int. J. Hydrogen Energy*, **2022**, 47, 181. |
|  |  | 1.0 M KOH | 101 mV | 55.58 mV·dec^−1^ |  |
|  |  | 1.0 M PBS | 114 mV | 77.35 mV·dec^−1^ |  |
| 299 | Rodlike CoP*^iR^* | 0.5 M H_2_SO_4_ | 127 mV | 53.97 mV·dec^−1^ |  |
|  |  | 1.0 M KOH | 173 mV | 67.48 mV·dec^−1^ |  |
|  |  | 1.0 M PBS | 240 mV | 131.69 mV·dec^−1^ |  |
| 300 | S, Fe-CoP@IF*^iR^* | 1.0 M KOH | − | 84.4 mV·dec^−1^ | Li et al., *Chem. Eng. J.*, **2023**, 470, 144081. |
| 301 | FeCoP@IF*^iR^* |  | − | 98.9 mV·dec^−1^ |  |
| 302 | H-CoP@NC*^iR^* | 1.0 M KOH | 173 mV | 59.9 mV·dec^−1^ | Liu et al., *J. Alloys Compd.*, **2023**, 956, 170315. |
| 303 | H-CoP*^iR^* |  | 266 mV | 75.7 mV·dec^−1^ |  |
| 304 | CoP*^iR^* |  | 304 mV | 82.8 mV·dec^−1^ |  |
| 305 | Mo-CoP/NC/TF*^iR^* | 0.5 M H_2_SO_4_ | 59 mV | 51.2 mV·dec^−1^ | Li et al., *Chem. Eng. J.*, **2021**, 405, 126981. |
|  |  | 1.0 M PBS | 130 mV | 84.1 mV·dec^−1^ |  |
|  |  | 1.0 M KOH | 78 mV | 48.1 mV·dec^−1^ |  |
| 306 | Mo-CoP/TF*^iR^* | 0.5 M H_2_SO_4_ | 83 mV | 66.3 mV·dec^−1^ |  |
|  |  | 1.0 M PBS | 168 mV | 90.5 mV·dec^−1^ |  |
|  |  | 1.0 M KOH | 100 mV | 51.2 mV·dec^−1^ |  |
| 307 | CoP/NC/TF*^iR^* | 0.5 M H_2_SO_4_ | 110 mV | 91.0 mV·dec^−1^ |  |
|  |  | 1.0 M PBS | 203 mV | 119.1 mV·dec^−1^ |  |
|  |  | 1.0 M KOH | 120 mV | 92.0 mV·dec^−1^ |  |
| 308 | Ni-CoP/CC | 1.0 M KOH | 53 mV | 57 mV·dec^−1^ | Du et al., *Electrochem. Commun.*, **2021**, 127, 107051. |
| 309 | Fe-CoP HNSs | 1.0 M KOH | 79 mV | 60.4 mV·dec^−1^ | Xu et al., *Int. J. Hydrogen Energy*, **2021**, 46, 26391. |
| 310 | CoP HNSs |  | 150.2 mV | 110.9 mV·dec^−1^ |  |
| 311 | O-CoP-2*^iR^* | 1.0 M KOH | 98 mV | 59.9 mV·dec^−1^ | Zhou et al., *Adv. Funct. Mater.*, **2020**, 30, 1905252. |
| 312 | O-CoP-1*^iR^* |  | 140 mV | 90.8 mV·dec^−1^ |  |
| 313 | O-CoP-3*^iR^* |  | 114 mV | 84.9 mV·dec^−1^ |  |
| 314 | CoP/CoS_x_ heterostructure | 0.5 M H_2_SO_4_ | 148 mV | 63 mV·dec^−1^ | Yu et al., *ChemElectroChem*, **2019**, 6, 3708. |
| 315 | CoP@CoMoO_4_/CC*^NiR^* | 1.0 M KOH | 81 mV | 65 mV·dec^−1^ | Liu et al., *J. Mater. Sci. Technol.*, **2020**, 46, 177. |
| 316 | CoP/CC*^NiR^* |  | 94.1 mV | 123 mV·dec^−1^ |  |
| 317 | CoP-NPC | 1.0 M KOH | 318 mV | 97.5 mV·dec^−1^ | Ahamad et al., *Mater. Lett.*, **2020**, 278, 128351. |
| 318 | Ni-CoP-5% | 1.0 M KOH | 88 mV | 41 mV·dec^−1^ | Zhao et al., *Nano Lett.*, **2021**, 21, 823. |
| 319 | Ni-CoP-10% |  | 98 mV | 43 mV·dec^−1^ |  |
| 320 | Ni-CoP-1% |  | 102 mV | 62 mV·dec^−1^ |  |
| 321 | Bare CoP |  | 146 mV | 58 mV·dec^−1^ |  |
| 322 | CoP NWs/CoP NPs@NC/CC*^iR^* | 1.0 M KOH | 103 mV | 87 mV·dec^−1^ | Fu et al., *Int. J. Energy Res.*, **2022**, 46, 11359. |
| 323 | CoP NWs/CC*^iR^* |  | 131 mV | 92 mV·dec^−1^ |  |
| 324 | CoP NPs@NC/CC*^iR^* |  | 190 mV | 130 mV·dec^−1^ |  |
| 325 | CoP/Co_2_P*^iR^* | 1.0 M KOH | 68 mV | 51.8 mV·dec^−1^ | Hua et al., *J. Energy Chem.*, **2019**, 37, 1. |
| 326 | CoP/Co_2_P-1*^iR^* |  | 98 mV | 70.2 mV·dec^−1^ |  |
| 327 | CoP*^iR^* |  | 75 mV | 61.1 mV·dec^−1^ |  |
| 328 | CoP/Ti-2.0*^iR^* | 0.5 M H_2_SO_4_ | 106 mV | 62.8 mV·dec^−1^ | Yan et al., *ACS Sustainable Chem. Eng.*, **2023**, 11, 2499. |
|  |  | 1.0 M KOH | 116 mV | 63.1 mV·dec^−1^ |  |
|  |  | 1.0 PBS | 131 mV | 79.5 mV·dec^−1^ |  |
| 329 | Pt_at_-CoP MNSs/CFC*^iR^* | 1.0 M KOH | 13 mV | 30.28 mV·dec^−1^ | Ye et al., *J. Mater. Chem. A*, **2020**, 8, 11246. |
| 330 | CoP NSs/CFC*^iR^* |  | 86.3 mV | 61.6 mV·dec^−1^ |  |
| 331 | Fe-CoP@CC-1 | 1.0 M KOH | 49 mV | 149 mV·dec^−1^ | Yang et al., *Int. J. Hydrogen Energy*, **2021**, 46, 28053. |
|  |  | 0.5 M H_2_SO_4_ | 80 mV | 82 mV·dec^−1^ |  |
| 332 | CoP/CN@MoS_2_*^iR^* | 0.5 M H_2_SO_4_ | 144 mV | 69 mV·dec^−1^ | Li et al., *ACS Appl. Mater. Interfaces*, **2019**, 11, 36649. |
|  |  | 1.0 M KOH | 149 mV | 88 mV·dec^−1^ |  |
| 333 | CoP/CN*^iR^* | 0.5 M H_2_SO_4_ | 163 mV | 70.5 mV·dec^−1^ |  |
|  |  | 1.0 M KOH | 182 mV | 101 mV·dec^−1^ |  |
| 334 | 1% C_60_(en)n-CoP-Co_2_P | 0.5 M H_2_SO_4_ | 129 mV | 59.71 mV·dec^−1^ | Yu et al., *Mater. Lett.*, **2023**, 352, 135184. |
|  |  | 1.0 M KOH | 117 mV | 72.49 mV·dec^−1^ |  |
| 335 | CoP-Co_2_P NPs | 0.5 M H_2_SO_4_ | 283 mV | 105.31 mV·dec^−1^ |  |
|  |  | 1.0 M KOH | 278 mV | 106.11 mV·dec^−1^ |  |
| 336 | CoP/CoN@NCNRs/CC*^NiR^* | 1.0 M KOH | 165 mV | 118 mV·dec^−1^ | Bai et al., *J. Mater. Chem. A*, **2024**, 12, 3997. |
| 337 | CoP/CC*^NiR^* |  | 335 mV | 184 mV·dec^−1^ |  |
| 338 | Co_2_P/CoP-350*^iR^* | 1.0 M KOH | 121 mV | 84.9 mV·dec^−1^ | Liu et al., *Nano Res.*, **2023**, 16, 12830. |
| 339 | Co_2_P/CoP-300*^iR^* |  | 168 mV | 97.5 mV·dec^−1^ |  |
| 340 | Co_2_P/CoP-400*^iR^* |  | 161 mV | 96.8 mV·dec^−1^ |  |
| 341 | CoP*^iR^* |  | 256 mV | 118.4 mV·dec^−1^ |  |
| 342 | CoP/MoS_2_-CNTs hybrid*^iR^* | 0.5 M H_2_SO_4_ | 12 mV | 42 mV·dec^−1^ | Pan et al., *Catal. Sci. Technol.*, **2016**, 6, 1611. |
| 343 | CoP/MoS_2_*^iR^* |  | − | 87 mV·dec^−1^ |  |
| 344 | CoP*^iR^* |  | − | 96 mV·dec^−1^ |  |
| 345 | CoP/NG | 0.5 M H_2_SO_4_ | 158 mV | 63.8 mV·dec^−1^ | Lu et al., *Electrochim. Acta*, **2019**, 307, 543. |
|  |  | 1.0 M KOH | 190 mV | 59.6 mV·dec^−1^ |  |
| 346 | CoP | 0.5 M H_2_SO_4_ | 262 mV | 77.5 mV·dec^−1^ |  |
|  |  | 1.0 M KOH | 229 mV | 67.56 mV·dec^−1^ |  |
| 347 | Okra-like hollow Cu_0.15_-CoP/Co_3_O_4_@CC | 1.0 M KOH | 13 mV | 95.8 mV·dec^−1^ | Du et al., *Int. J. Hydrogen Energy*, **2022**, 47, 7168. |
| 348 | CoP-Co_x_O_y_/CC*^iR^* | 1.0 M KOH | 43 mV | 51.4 mV·dec^−1^ | Alsabban et al., *ACS Nano*, **2022**, 16, 3906. |
| 349 | CoP/NCF-200 | 0.5 M H_2_SO_4_ | 86 mV | 55 mV·dec^−1^ | Tong et al., *ACS Sustainable Chem. Eng.*, **2019**, 7, 17432. |
|  |  | 1.0 M KOH | 141 mV | 84 mV·dec^−1^ |  |
| 350 | CoP/NC | 0.5 M H_2_SO_4_ | 174 mV | 69 mV·dec^−1^ |  |
|  |  | 1.0 M KOH | 245 mV | 164 mV·dec^−1^ |  |
| 351 | CoP/NCF-100 | 0.5 M H_2_SO_4_ | 143 mV | 62 mV·dec^−1^ |  |
|  |  | 1.0 M KOH | 155 mV | 111 mV·dec^−1^ |  |
| 352 | CoP/NCF-300 | 0.5 M H_2_SO_4_ | 152 mV | 66 mV·dec^−1^ |  |
|  |  | 1.0 M KOH | 180 mV | 142 mV·dec^−1^ |  |
| 353 | CoP@glC*^iR^* | 0.5 M H_2_SO_4_ | 160 mV | 87.5 mV·dec^−1^ | Wang et al., *J. Catal.*, **2024**, 430, 115310. |
| 354 | CoP@N-glC-1*^iR^* |  | 178 mV | 80.9 mV·dec^−1^ |  |
| 355 | CoP@N-glC-2*^iR^* |  | 191 mV | 76.7 mV·dec^−1^ |  |
| 356 | CoP@N-glC-3*^iR^* |  | 202 mV | 74.4 mV·dec^−1^ |  |
| 357 | CoP@N-glC-4*^iR^* |  | 216 mV | 82.9 mV·dec^−1^ |  |
| 358 | CoP@N-glC-5*^iR^* |  | 227 mV | 84.4 mV·dec^−1^ |  |
| 359 | rGO-CoP/Co_2_P microflower*^iR^* | 0.5 M H_2_SO_4_ | 156 mV | 53.8 mV·dec^−1^ | Kim et al., *J. Am. Ceram. Soc.*, **2018**, 101, 3749. |
| 360 | CoP/Co_2_P*^iR^* |  | 168 mV | 67.0 mV·dec^−1^ |  |
| 361 | CoP-NC/CBC | 0.5 M H_2_SO_4_ | 182 mV | 68.67 mV·dec^−1^ | Wang et al., *Int. J. Biol. Macromol.*, **2023**, 242, 125173. |
|  |  | 1.0 M KOH | 151 mV | 67.38 mV·dec^−1^ |  |
| 362 | CoP-NC | 0.5 M H_2_SO_4_ | 342 mV | 105.27 mV·dec^−1^ |  |
|  |  | 1.0 M KOH | 212 mV | 102.67 mV·dec^−1^ |  |
| 363 | CoP-CoS composite | 0.5 M H_2_SO_4_ | 156 mV | 74 mV·dec^−1^ | Sidek et al., *ChemCatChem*, **2019**, 11, 6099. |
|  |  | 1.0 M KOH | 227 mV | 91 mV·dec^−1^ |  |
| 364 | CoP@CNF*^iR^* | 1.0 M KOH | 127 mV | 73.4 mV·dec^−1^ | Ren et al., *Int. J. Hydrogen Energy*, **2024**, 51, 490. |
| 365 | Ni_2_P-CoP/MoO_2_ | 1.0 M KOH | 33 mV | 81 mV·dec^−1^ | Tan et al., *J. Power Sources*, **2024**, 611, 234757. |
|  |  | 1.0 M PBS | 41 mV | 60 mV·dec^−1^ |  |
|  |  | 0.5 M H_2_SO_4_ | 23 mV | 41 mV·dec^−1^ |  |
| 366 | W-CoP NAs/CC*^iR^* | 0.5 M H_2_SO_4_ | 89 mV | 58 mV·dec^−1^ | Wang et al., *Small*, **2019**, 15, 1902613. |
|  |  | 1.0 M KOH | 94 mV | 63 mV·dec^−1^ |  |
|  |  | 1.0 M PBS | 102 mV | 87 mV·dec^−1^ |  |
| 367 | CoP NAs/CC*^iR^* | 0.5 M H_2_SO_4_ | 123 mV | 97 mV·dec^−1^ |  |
|  |  | 1.0 M KOH | 129 mV | 74 mV·dec^−1^ |  |
|  |  | 1.0 M PBS | 132 mV | 98 mV·dec^−1^ |  |
| 368 | CoP@Fe-CoP/NC/NF*^iR^* | 0.5 M H_2_SO_4_ | 38 mV | 30 mV·dec^−1^ | Mei et al., *Inorg. Chem.*, **2022**, 61, 1159. |
|  |  | 1.0 M KOH | 97 mV | 49 mV·dec^−1^ |  |
| 369 | CoP/NC/NF*^iR^* | 0.5 M H_2_SO_4_ | 60 mV | 63 mV·dec^−1^ |  |
|  |  | 1.0 M KOH | − | 84 mV·dec^−1^ |  |
| 370 | CoP/CoP_2_ | 1.0 M KOH | − | 84.2 mV·dec^−1^ | Li et al., *ChemElectroChem*, **2018**, 5, 701. |
|  |  | 0.5 M H_2_SO_4_ | − | 90 mV·dec^−1^ |  |
| 371 | CoP hollow prisms*^iR^* | 0.5 M H_2_SO_4_ | − | 57 mV·dec^−1^ | Liu et al., *Electrochim. Acta*, **2016**, 220, 98. |
|  |  | 1.0 M KOH | − | 67 mV·dec^−1^ |  |
| 372 | CoP-CNT/NG | 0.5 M H_2_SO_4_ | 155 mV | 69.1 mV·dec^−1^ | Guan et al., *Int. J. Hydrogen Energy*, **2019**, 44, 30053. |
| 373 | CoP–RGO |  | 192 mV | 82.2 mV·dec^−1^ |  |
| 374 | CoP |  | 235 mV | 89.4 mV·dec^−1^ |  |
| 375 | CoP-CNT/NG | 1.0 M KOH | 176 mV | − |  |
| 376 | Mn_10_-doped CoP/NF*^iR^* | 1.0 M KOH | 60 mV | 56.70 mV·dec^−1^ | Meng et al., *Inorg. Chem.*, **2022**, 61, 9832. |
| 377 | Mn_5_-doped CoP/NF*^iR^* |  | 81 mV | 58.79 mV·dec^−1^ |  |
| 378 | Mn_20_-doped CoP/NF*^iR^* |  | 73 mV | 59.99 mV·dec^−1^ |  |
| 379 | Mn_30_-doped CoP/NF*^iR^* |  | 78 mV | 63.52 mV·dec^−1^ |  |
| 380 | CoP/NF*^iR^* |  | 92 mV | 60.42 mV·dec^−1^ |  |
| 381 | CoP-60 | 0.5 M H_2_SO_4_ | 121 mV | 56 mV·dec^−1^ | Guo et al., *Int. J. Hydrogen Energy*, **2018**, 43, 2034. |
| 382 | CoP-30 |  | 144 mV | 59 mV·dec^−1^ |  |
| 383 | CoP-15 |  | 162 mV | 61 mV·dec^−1^ |  |
| 384 | CoP-0 |  | 183 mV | 63 mV·dec^−1^ |  |
| 385 | CoP-RuP_2_/rGO*^iR^* | 0.5 M H_2_SO_4_ | 139.1 mV | 54.4 mV·dec^−1^ | Liu et al., *Cryst. Growth Des.*, **2022**, 22, 5607. |
|  |  | 1.0 M KOH | 173.9 mV | 81 mV·dec^−1^ |  |
|  |  | 1.0 M PBS | 308.9 mV | 110.8 mV·dec^−1^ |  |
| 386 | CoP/Co/C-6*^iR^* | 1.0 M KOH | 132 mV | 57 mV·dec^−1^ | Yang et al., *J. Colloid Interface Sci.*, **2022**, 623, 808. |
| 387 | CoP/NiCoP/NC*^NiR^* | 1.0 M KOH | 75 mV | 64 mV·dec^−1^ | Boppella et al., *Adv. Funct. Mater.*, **2019**, 29, 1807976. |
|  |  | 0.5 M H_2_SO_4_ | 60 mV | 58 mV·dec^−1^ |  |
|  |  | 1.0 M PBS | 123 mV | 78 mV·dec^−1^ |  |
| 388 | POC–CoP NSs*^iR^* | 1.0 M KOH | 63 mV | 45 mV·dec^−1^ | Gao et al., *J. Power Sources*, **2025**, 643, 237069. |
| 389 | 2D CoP/Co-MOF*^iR^* | 0.5 M H_2_SO_4_ | 52 mV | 49 mV·dec^−1^ | Li et al., *J. Electroanal. Chem.*, **2021**, 895, 115514. |
|  |  | 1.0 M PBS | 106 mV | 89 mV·dec^−1^ |  |
|  |  | 1.0 M KOH | 26 mV | 53 mV·dec^−1^ |  |
| 390 | CoSe_2_\|CoP at 30 min*^iR^* | 0.5 M H_2_SO_4_ | 140 mV | 42 mV·dec^−1^ | Bose et al., *ChemistrySelect*, **2017**, 2, 10661. |
| 391 | Co_2_P/CoP NAs*^NiR^* | 1.0 M KOH | 40 mV | 57 mV·dec^−1^ | Zhang et al., *Nano-Micro Lett.*, **2020**, 12, 154. |
| 392 | CoP-NCNT@MoS_2_*^NiR^* | 1.0 M KOH | 123 mV | 55 mV·dec^−1^ | Wang et al., *J. Alloys Compd.*, **2020**, 813, 152211. |
| 393 | Fe-CoP/NF*^iR^* | 1.0 M KOH | 74 mV | 67 mV·dec^−1^ | Li et al., *Appl. Catal. B Environ. Energy*, **2024**, 346, 123749. |
| 394 | Cr-CoP/NF*^iR^* |  | 79 mV | 70 mV·dec^−1^ |  |
| 395 | CoP/NF*^iR^* |  | 89 mV | 81 mV·dec^−1^ |  |
| 396 | CoP/Ni_2_P@NC | 0.5 M H_2_SO_4_ | 91 mV | 62 mV·dec^−1^ | Lv et al., *Mater. Chem. Front.*, **2019**, 3, 2428. |
|  |  | 1.0 M KOH | 143 mV | 76 mV·dec^−1^ |  |
| 397 | CoP/Co_3_O_4_-fC-pPVP*^iR^* | 1.0 M KOH | 166 mV | 56 mV·dec^−1^ | Liu et al, *Electrochim. Acta*, **2022**, 412, 140134. |
| 398 | Fe-CoP UNSs*^iR^* | 1.0 M KOH | 67 mV | 66.22 mV·dec^−1^ | Li et al., *J. Mater. Chem. A*, **2019**, 7, 20658. |
| 399 | NiCoP-CoP@NNA*^iR^* | 1.0 M KOH | 20 mV | 28 mV·dec^−1^ | Liu et al., *J. Mater. Chem. A*, **2023**, 11, 22340. |
| 400 | CoP@Ni plate*^iR^* |  | 118 mV | 37 mV·dec^−1^ |  |
| 401 | CoP@C*^iR^* |  | 131 mV | 45 mV·dec^−1^ |  |
| 402 | CoP@NF*^iR^* |  | 61 mV | 57 mV·dec^−1^ |  |
| 403 | CoP-NB*^iR^* | 0.5 M H_2_SO_4_ | − | 51 mV·dec^−1^ | Wang et al., *ACS Nano*, **2018**, 12, 12238. |
| 404 | CoP@NC composite foam | 1.0 M KOH | 151.3 mV | 64.8 mV·dec^−1^ | Wang et al., *Appl. Surf. Sci.*, **2020**, 505, 144503. |
| 405 | CoP/CNFs*^iR^* | 1.0 M KOH | 225 mV | 100.8 mV·dec^−1^ | Xie et al., *J. Energy Chem.*, **2022**, 64, 503. |
| 406 | Mo-CoP*^iR^* | 1.0 M KOH | 49 mV | 80 mV·dec^−1^ | Liu et al., *Energy Technol.*, **2019**, 7, 1900021. |
| 407 | CoP*^iR^* |  | 101 mV | 82 mV·dec^−1^ |  |
| 408 | Co(OH)_2_/CoP*^iR^* | 1.0 M KOH | 77 mV | 52 mV·dec^−1^ | Jiang et al., *Int. J. Hydrogen Energy*, **2021**, 46, 8497. |
| 409 | CoP/C-1 | 0.5 M H_2_SO_4_ | 157 mV | 56 mV·dec^−1^ | Li et al., *J. Energy Chem.*, **2020**, 43, 121. |
| 410 | CoP/Co_2_P@NC/Ti*^iR^* | 0.5 M H_2_SO_4_ | 31 mV | 44 mV·dec^−1^ | Liu et al., *iScience*, **2020**, 23, 101264. |
|  |  | 1.0 M KOH | 49 mV | 51 mV·dec^−1^ |  |
|  |  | 1.0 M PBS | 64 mV | 98 mV·dec^−1^ |  |
| 411 | Fe/Zn-CoP core–shell arrays*^iR^* | 1.0 M KOH | 75 mV | 57.9 mV·dec^−1^ | Wang et al., *Catal. Sci. Technol.*, **2020**, 10, 1395. |
| 412 | V-CoP/Ni_2_P/NF*^iR^* | 1.0 M KOH | 20 mV | 54.2 mV·dec^−1^ | Wang et al., *Int. J. Hydrogen Energy*, **2022**, 47, 18305. |
|  |  | 1.0 M PBS | 58 mV | 88.7 mV·dec^−1^ |  |
|  |  | 0.5 M H_2_SO_4_ | 79 mV | 58.3 mV·dec^−1^ |  |
| 413 | CoP/NCS-400*^NiR^* | 1.0 M KOH | 71 mV | 109 mV·dec^−1^ | Yin et al., *Electrochim. Acta*, **2018**, 276, 362. |
| 414 | N-CoP/NH_2_-BP*^iR^* | 1.0 M KOH | 90 mV | 64 mV·dec^−1^ | Fang et al., *Dalton Trans.*, **2023**, 52, 12436. |
| 415 | CoP/BP*^iR^* |  | 142 mV | 89 mV·dec^−1^ |  |
| 416 | CoP@OMC | 1.0 M KOH | 111 mV | 43.8 mV·dec^−1^ | Mohammadi et al., *Mater. Today Commun.*, **2024**, 39, 109165. |
| 417 | Ce,F-Ni_2_P/CoP | 1.0 M KOH | 88 mV | 55.8 mV·dec^−1^ | Peng et al., *Appl. Surf. Sci.*, **2025**, 679, 161258. |
|  |  | 0.5 M H_2_SO_4_ | 66 mV | 41.2 mV·dec^−1^ |  |
|  |  | 1.0 M PBS | 108 mV | 106.4 mV·dec^−1^ |  |
| 418 | Co/CoP@NC*^iR^* | 0.5 M H_2_SO_4_ | 117 mV | 51.3 mV·dec^−1^ | Li et al., *Energy Storage Mater.*, **2018**, 12, 44. |
|  |  | 1.0 M KOH | 180 mV | 60.3 mV·dec^−1^ |  |
| 419 | CoP_(MBMG)_/CNTs | 0.5 M H_2_SO_4_ | 135 mV | 58 mV·dec^−1^ | Li et al, *Int. J. Hydrogen Energy*, **2017**, 42, 21786. |
| 420 | N-VGSs@CB/CoP*^iR^* | 1.0 M KOH | 114 mV | 86 mV·dec^−1^ | Wu et al., *Energy Fuels*, **2023**, 37, 19156. |
| 421 | Ni-Co_20_-P@NF*^iR^* | 1.0 M KOH | 67.0 mV | 42.7 mV·dec^−1^ | Guo et al., *Appl. Catal. B Environ.*, **2020**, 265, 118584. |
| 422 | Zn_0.08_Co_0.92_P/TM*^NiR^* | 0.5 M H_2_SO_4_ | 39 mV | 39 mV·dec^−1^ | Liu et al., *Adv. Energy Mater.*, **2017**, 7, 1700020. |
|  |  | 1.0 M KOH | 67 mV | − |  |
| 423 | CoP-N/Co*^iR^* | 1.0 M KOH | 60 mV | 50.9 mV·dec^−1^ | Liu et al., *J. Mater. Chem. A*, **2019**, 7, 13242. |
| 424 | Ni_2_P/CoP-40*^iR^* | 1.0 M PBS | 65.2 mV | 73.3 mV·dec^−1^ | Tan et al., *Energy Environ. Mater.*, **2023**, 6, e12398. |
|  |  | 1.0 M KOH | 87.8 mV | 63.9 mV·dec^−1^ |  |
| 425 | Ni_2_P-CoP | 0.5 M H_2_SO_4_ | 105 mV | 64 mV·dec^−1^ | Liang et al., *ACS Appl. Mater. Interfaces*, **2017**, 9, 23222. |
| 426 | CoP@NPC | 1.0 M KOH | 132 mV | 72.6 mV·dec^−1^ | Tian et al., *Int. J. Hydrogen Energy*, **2025**, 124, 1. |
| 427 | N-CoP/Ni_2_P@NF*^iR^* | 1.0 M KOH | 46 mV | 66.4 mV·dec^−1^ | Jian et al, *Inorg. Chem.*, **2021**, 60, 18544. |
| 428 | CoP@NF*^iR^* |  | 87 mV | 108.18 mV·dec^−1^ |  |
| 429 | CoP/Ni_2_P@NF*^iR^* |  | 56 mV | 85.4 mV·dec^−1^ |  |
| 430 | CoP/NSGO | 0.5 M H_2_SO_4_ | − | 52 mV·dec^−1^ | Lin et al., *Electrochim. Acta*, **2016**, 222, 246. |
| 431 | CoP/NGO |  | − | 65 mV·dec^−1^ |  |
| 432 | CoP/GO |  | − | 81 mV·dec^−1^ |  |
| 433 | CoP/P-NiO/NF*^iR^* | 1.0 M KOH | 52 mV | 73.6 mV·dec^−1^ | Nie et al., *J. Colloid Interface Sci.*, **2022**, 621, 213. |
| 434 | CoP/NF*^iR^* |  | 75 mV | 82.4 mV·dec^−1^ |  |
| 435 | CoFe PBA@CoP*^iR^* | 1.0 M KOH | 100 mV | 60.8 mV·dec^−1^ | Quan et al., *Small Methods*, **2021**, 5, 2100125. |
| 436 | CoO-CoP*^iR^* |  | 127 mV | 117.6 mV·dec^−1^ |  |
| 437 | NiFeP/CoP/CC | 1.0 M KOH | 96.38 mV | 70.67 mV·dec^−1^ | Li et al., *Nanotechnology*, **2019**, 30, 485402. |
|  |  | 0.5 M H_2_SO_4_ | 78.80 mV | 63.21 mV·dec^−1^ |  |
| 438 | CoP/NF | 1.0 M KOH | 130 mV | 79 mV·dec^−1^ | Xiao et al., *Int. J. Hydrogen Energy*, **2022**, 47, 9915. |
| 439 | NF/CoP@Ni_3_S_2_*^iR^* | 1.0 M KOH | 68.9 mV | 115.4 mV·dec^−1^ | Wen et al., *Appl. Surf. Sci.*, **2022**, 586, 152736. |
| 440 | Zn-CoP/CF*^iR^* | 1.0 M KOH | − | 82.9 mV·dec^−1^ | Li et al., *Catal. Sci. Technol.*, **2023**, 13, 6550. |
| 441 | CoP/CF*^iR^* |  | − | 123.2 mV·dec^−1^ |  |
| 442 | CoP/Ni_2_P/CC-4*^iR^* | 0.5 M H_2_SO_4_ | 67 mV | 48 mV·dec^−1^ | Yang et al., *Int. J. Hydrogen Energy*, **2022**, 47, 20448. |
|  |  | 1.0 M KOH | 71 mV | 64 mV·dec^−1^ |  |
|  |  | 1.0 M PBS | 95 mV | 69 mV·dec^−1^ |  |
| 443 | CoP/CeO_x_-20:1*^NiR^* | 1.0 M KOH | 118 mV | 77.26 mV·dec^−1^ | Song et al., *ACS Appl. Mater. Interfaces*, **2022**, 14, 33151. |
| 444 | CoP/CeO_x_-10:1*^NiR^* |  | 129 mV | 96.69 mV·dec^−1^ |  |
| 445 | B-CoP/NF*^iR^* | 1.0 M KOH | 55 mV | 96.71 mV·dec^−1^ | Jia et al., *J. Alloys Compd.*, **2024**, 984, 173974. |
| 446 | 0.30CoP-2-900 | 0.5 M H_2_SO_4_ | 193 mV | 71.4 mV·dec^−1^ | Deng et al., *J. Solid State Chem.*, **2025**, 349, 125416. |
| 447 | Zn-CoP*^iR^* | 1.0 M KOH | 36 mV | 57 mV·dec^−1^ | Zhang et al., *J. Power Sources*, **2025**, 632, 236276. |
| 448 | CoP/CC | 0.5 M H_2_SO_4_ | 57 mV | 75 mV·dec^−1^ | Niu et al., *ACS Sustainable Chem. Eng.*, **2019**, 7, 2335. |
| 449 | CoP-FeP/CC | 0.5 M H_2_SO_4_ | 30 mV | 42 mV·dec^−1^ |  |
|  |  | 1.0 M KOH | 71 mV | 67 mV·dec^−1^ |  |
| 450 | Ni_2_P/CoP/P,F–C*^iR^* | 1.0 M KOH | 65 mV | 72 mV·dec^−1^ | Wu et al., *J. Power Sources*, **2022**, 548, 232065. |
| 451 | CoP/P,F–C*^iR^* |  | 140 mV | 89 mV·dec^−1^ |  |
| 452 | CoP/P–C*^iR^* |  | 209 mV | 114 mV·dec^−1^ |  |
| 453 | CoP/NF*^iR^* | 1.0 M KOH | 41.1 mV | 65.3 mV·dec^−1^ | Liu et al., *J. Mater. Chem. A*, **2020**, 8, 19254. |
|  |  | 1.0 PBS | 55.2 mV | 68.7 mV·dec^−1^ |  |
|  |  | 0.5 M H_2_SO_4_ | 83.9 mV | 55 mV·dec^−1^ |  |
| 454 | CoP/Mo_2_C-NC*^iR^* | 0.5 M H_2_SO_4_ | 55.7 mV | 49 mV·dec^−1^ | Luo et al., *ACS Appl. Mater. Interfaces*, **2018**, 10, 42335. |
|  |  | 1.0 M KOH | 67.2 mV | 66 mV·dec^−1^ |  |
|  |  | 1.0 M PBS | 81.8 mV | 68 mV·dec^−1^ |  |
| 455 | Ce-doped CoP*^iR^* | 0.5 M H_2_SO_4_ | 54 mV | 54.0 mV·dec^−1^ | Gao et al., *Nano Energy*, **2017**, 38, 290. |
|  |  | 1.0 M KOH | 92 mV | 63.5 mV·dec^−1^ |  |
| 456 | CoP*^iR^* | 0.5 M H_2_SO_4_ | 74 mV | 59.3 mV·dec^−1^ |  |
|  |  | 1.0 M KOH | 139 mV | 70.3 mV·dec^−1^ |  |
| 457 | Er-doped CoP NMs | 0.5 M H_2_SO_4_ | 52 mV | 32 mV·dec^−1^ | Zhang et al., *J. Mater. Chem. A*, **2019**, 7, 5769. |
|  |  | 1.0 M KOH | 66 mV | 61 mV·dec^−1^ |  |
| 458 | CoP | 0.5 M H_2_SO_4_ | 63 mV | 45 mV·dec^−1^ |  |
|  |  | 1.0 M KOH | 84 mV | 85 mV·dec^−1^ |  |
| 459 | CoP | 0.5 M H_2_SO_4_ | − | 54.8 mV·dec^−1^ | Li et al., *J. Mater. Chem. A*, **2015**, 3, 13066. |
| 460 | Ce,Zr-CoP/NF*^iR^* | 1.0 M KOH | 50 mV | 52 mV·dec^−1^ | Lu et al., *J. Alloys Compd.*, **2025**, 1023, 180155. |
| 461 | CoP-SSM*^iR^* | 1.0 M KOH | 266 mV | 110 mV·dec^−1^ | Alharbi et al., *Int. J. Hydrogen Energy*, **2023**, 48, 31172. |
| 462 | CoP NBAs/Ti | 0.5 M H_2_SO_4_ | 203 mV | 40 mV·dec^−1^ | Niu et al., *Electrochem. Commun.*, **2015**, 56, 56. |
|  |  | 1.0 M KOH | − | 115 mV·dec^−1^ |  |
|  |  | 1.0 M PBS | − | 116 mV·dec^−1^ |  |
| 463 | CNF@CoP-15-CNTs*^iR^* | 0.5 M H_2_SO_4_ | 65 mV | 80.79 mV·dec^−1^ | Yu et al., *Sustain. Energy Fuels*, **2022**, 6, 5000. |
| 464 | Ru/CoP NSs | 1.0 M KOH | 38 mV | 57 mV·dec^−1^ | Dai et al., *Sustain. Energy Fuels*, **2022**, 6, 4982. |
|  |  | 1.0 M PBS | 149 mV | 113 mV·dec^−1^ |  |
| 465 | 3-D CNF@CoP/NC*^iR^* | 0.5 M H_2_SO_4_ | 64.5 mV | 48.6 mV·dec^−1^ | Wang et al., *Nanoscale*, **2021**, 13, 14705. |
|  |  | 1.0 M KOH | 105.6 mV | 53.9 mV·dec^−1^ |  |
| 466 | FeP4/CoP@NF-7.5 | 1.0 M KOH | 45 mV | 83.55 mV·dec^−1^ | Yang et al., *J. Ind. Eng. Chem.*, **2024**, 132, 201. |
| 467 | CoP-WP/rGO*^iR^* | 1.0 M KOH | 96 mV | 62 mV·dec^−1^ | Jiao et al., *Sci. China Mater.*, **2022**, 65, 1225. |
|  |  | 0.5 M H_2_SO_4_ | 130 mV | 54 mV·dec^−1^ |  |
|  |  | 1.0 M PBS | 138 mV | 80 mV·dec^−1^ |  |
| 468 | Ni_2_P/CoP/NF*^NiR^* | 1.0 M KOH | − | 43.8 mV·dec^−1^ | Niu et al., *ACS Appl. Mater. Interfaces*, **2024**, 16, 31480. |
| 469 | CoP/CC*^NiR^* | 1.0 M KOH | − | 146.6 mV·dec^−1^ |  |
| 470 | Ni@C-CoP-3*^iR^* | 1.0 M KOH | 106 mV | − | Luo et al., *J. Mater. Sci. Technol.*, **2023**, 166, 164. |
|  |  | 0.5 M H_2_SO_4_ | 116 mV | − |  |
| 471 | Ru-CoP/CC | 1.0 M KOH | 44 mV | 64 mV·dec^−1^ | Liu et al., *Angew. Chem. Int. Ed.*, **2024**, 63, e202400069. |
| 472 | N-CoP/CC*^iR^* | 1.0 M PBS | 74 mV | 69 mV·dec^−1^ | Men et al., *Appl. Catal. B Environ.*, **2019**, 253, 21. |
|  |  | 1.0 M KOH | 39 mV | 58 mV·dec^−1^ |  |
|  |  | 0.5 M H_2_SO_4_ | 25 mV | 49 mV·dec^−1^ |  |
| 473 | CoP/CC*^iR^* | 1.0 M PBS | 136 mV | 117 mV·dec^−1^ |  |
|  |  | 1.0 M KOH | 87 mV | 72 mV·dec^−1^ |  |
|  |  | 0.5 M H_2_SO_4_ | 79 mV | 61 mV·dec^−1^ |  |
| 474 | CoP/NiCoP hollow nanoflowers*^iR^* | 1.0 M KOH | 110.4 mV | 83.7 mV·dec^−1^ | Fu et al., *J. Colloid Interface Sci.*, **2024**, 653, 1272. |
| 475 | CoP*^iR^* |  | 151.3 mV | 92.1 mV·dec^−1^ |  |
| 476 | V-doped CoP*^iR^* | 1.0 M KOH | 235 mV | 86.1 mV·dec^−1^ | Qin et al., *J. Energy Chem.*, **2019**, 39, 182. |
| 477 | CoP@PCM*^iR^* | 0.5 M H_2_SO_4_ | 155 mV | 58.8 mV·dec^−1^ | Min et al., *ChemCatChem*, **2020**, 12, 3929. |
|  |  | 1.0 M KOH | 112 mV | 75.4 mV·dec^−1^ |  |
| 478 | FeCoP/NF-30*^iR^* | 1.0 M KOH | 66.60 mV | 48.34 mV·dec^−1^ | Liu et al., *J. Electroanal. Chem.*, **2025**, 987, 119116. |
| 479 | CoP/NF*^iR^* |  | 96.2 mV | 92.9 mV·dec^−1^ |  |
| 480 | CoP-FeP-MoP_4_@NC*^iR^* | 1.0 M KOH | 75 mV | 97.54 mV·dec^−1^ | Cao et al., *ACS Appl. Mater. Interfaces*, **2025**, 17, 15259. |
| 481 | Zn-Co-P*^NiR^* | 0.5 M H_2_SO_4_ | − | 170 mV·dec^−1^ | Jing et al., *ACS Appl. Nano Mater.*, **2019**, 2, 5922. |
|  |  | 1.0 M KOH | − | 116 mV·dec^−1^ |  |
| 482 | m-CoP–NiCoP/CC*^iR^* | 0.5 M H_2_SO_4_ | 75 mV | 57.2 mV·dec^−1^ | Lin et al., *J. Mater. Sci.*, **2021**, 56, 3375. |
| 483 | u-CoP@NC*^iR^* | 0.5 M H_2_SO_4_ | 131 mV | 62.7 mV·dec^−1^ | Liu et al., *Int. J. Hydrogen Energy*, **2020**, 45, 1729. |
|  |  | 1.0 M KOH | 111 mV | 70.3 mV·dec^−1^ |  |
| 484 | Di-CoP/C*^iR^* | 0.5 M H_2_SO_4_ | 136 mV | 49.3 mV·dec^−1^ | Duan et al., *J. Electroanal. Chem.*, **2021**, 892, 115300. |
| 485 | CoP/C*^iR^* | 0.5 M H_2_SO_4_ | 42.1 mV | 59 mV·dec^−1^ | Wang et al., *Nano Energy*, **2021**, 84, 105943. |
| 486 | CoP(C/Z)-0.6*^NiR^* | 1.0 M KOH | 154 mV | 85.1 mV·dec^−1^ | Meng et al., *Inorg. Chem. Commun.*, **2021**, 134, 109058. |
| 487 | Pure-phase single-crystalline CoP | 1.0 M KOH | 120 mV | 100 mV·dec^−1^ | Chen et al., *Chin. Chem. Lett.*, **2022**, 33, 3752. |
| 488 | CoP nanorod assemblies | 0.5 M H_2_SO_4_ | 181 mV | 69 mV·dec^−1^ | Li et al., *RSC Adv.*, **2015**, 5, 90265. |
| 489 | NC@Fe_0.1_-CoP/Co_2_P/NF*^iR^* | 1.0 M KOH | − | 91.0 mV·dec^−1^ | Huo et al., *J. Colloid Interface Sci.*, **2025**, 678, 669. |
| 490 | CoP/Co-MOF*^iR^* | 1.0 M PBS | 49 mV | 63 mV·dec^−1^ | Liu et al., *Angew. Chem. Int. Ed.*, **2019**, 58, 4679. |
|  |  | 1.0 M KOH | 34 mV | 56 mV·dec^−1^ |  |
|  |  | 0.5 M H_2_SO_4_ | 27 mV | 43 mV·dec^−1^ |  |
| 491 | CoP*^iR^* | 1.0 M PBS | 97 mV | 89 mV·dec^−1^ |  |
|  |  | 1.0 M KOH | 75 mV | 65 mV·dec^−1^ |  |
|  |  | 0.5 M H_2_SO_4_ | 65 mV | 57 mV·dec^−1^ |  |
| 492 | Ce_0.04_Co_0.96_P*^iR^* | 1.0 M KOH | 152 mV | 74.8 mV·dec^−1^ | Ding et al., *J. Energy Chem.*, **2025**, 101, 598. |
| 493 | Porous CoP CPH | 0.5 M H_2_SO_4_ | 133 mV | 51 mV·dec^−1^ | Xu et al., *J. Mater. Chem. A*, **2015**, 3, 21471. |
| 494 | CoP-C/NiO/NF*^NiR^* | 1.0 M KOH | 72 mV | 51 mV·dec^−1^ | Wang et al., *Dalton Trans.*, **2021**, 50, 17549. |
| 495 | CoP/Ti_3_C_2_*^iR^* | 1.0 M KOH | 102 mV | 68.7 mV·dec^−1^ | Yan et al., *J. Mater. Chem. A*, **2020**, 8, 14234. |
|  |  | 0.5 M H_2_SO_4_ | 71 mV | 57.6 mV·dec^−1^ |  |
| 496 | p-FeP/CoP/CP*^iR^* | 0.5 M H_2_SO_4_ | 33 mV | 43 mV·dec^−1^ | Gao et al., *J. Power Sources*, **2025**, 630, 236092. |
|  |  | 1.0 M KOH | 56 mV | 60 mV·dec^−1^ |  |
| 497 | CoP·5CoMoP NC*^iR^* | 1.0 M KOH | 72 mV | 60.3 mV·dec^−1^ | Zhang et al., *J. Colloid Interface Sci.*, **2022**, 615, 465. |
|  |  | 0.5 M H_2_SO_4_ | 44 mV | 51.2 mV·dec^−1^ |  |
|  |  | 1.0 M PBS | 151 mV | 74.5 mV·dec^−1^ |  |
| 498 | (CoP)_0.54_-(FeP)_0.46_-NRs/G*^iR^* | 0.5 M H_2_SO_4_ | 57 mV | 52 mV·dec^−1^ | Liu et al., *Small*, **2017**, 13, 1700092. |
|  |  | 1.0 M KOH | 97 mV | 62 mV·dec^−1^ |  |
| 499 | CoP@NCDs_0.5_/NF*^iR^* | 1.0 M KOH | 103 mV | 86.3 mV·dec^−1^ | Sun et al., *J. Cryst. Growth*, **2023**, 624, 127430. |
| 500 | W_0.03_-CoP@NCDs_0.5_/NF*^iR^* |  | 56 mV | 79.1 mV·dec^−1^ |  |
| 501 | CoP@NC-NG*^iR^* | 0.5 M H_2_SO_4_ | 135 mV | 59.3 mV·dec^−1^ | Ma et al., *Small*, **2018**, 14, 1702895. |
| 502 | CoP@NC | 1.0 M KOH | − | 88.2 mV·dec^−1^ | Zhao et al., *J. Alloys Compd.*, **2022**, 927, 167057. |
|  |  | 0.5 M H_2_SO_4_ | 160 mV | 57.2 mV·dec^−1^ |  |
| 503 | O-CoP*^iR^* | 1.0 M KOH | 125 mV | 68 mV·dec^−1^ | Zhang et al., *Chem. Eur. J.*, **2023**, 29, e202301252. |
| 504 | 3D rectangular CoP NS/CC*^iR^* | 0.5 M H_2_SO_4_ | 92 mV | 58 mV·dec^−1^ | Yan et al., *New J. Chem.*, **2017**, 41, 2436. |
| 505 | Ho-Co_3_O_4_@CoP/NS-RGO | 0.5 M H_2_SO_4_ | 54 mV | 62 mV·dec^−1^ | Liu et al., *Energy Fuels*, **2022**, 36, 8, 4532. |
| 506 | CoP/NCNWs*^NiR^* | 0.5 M H_2_SO_4_ | 95 mV | 50 mV·dec^−1^ | Yu et al., *Electrochim. Acta*, **2019**, 299, 423. |
|  |  | 1.0 M KOH | 154 mV | 59 mV·dec^−1^ |  |
| 507 | Urchin-like CoP nanocrystals*^iR^* | 0.5 M H_2_SO_4_ | − | 46 mV·dec^−1^ | Yang et al., *Nano Lett.*, **2015**, 15, 7616. |
| 508 | CoP-NT | 0.5 M H_2_SO_4_ | 152 mV | 50 mV·dec^−1^ | Miao et al., *Nanoscale*, **2017**, 9, 16313. |
| 509 | CoP/DCS*^iR^* | 1.0 M KOH | 88 mV | 56 mV·dec^−1^ | Wu et al., *Carbon Energy*, **2023**, 5, e268. |
|  |  | 0.5 M H_2_SO_4_ | 136 mV | 87 mV·dec^−1^ |  |
|  |  | 1.0 M PBS | 214 mV | 107 mV·dec^−1^ |  |
| 510 | Cu-CoP NRAs/CC | 0.5 M H_2_SO_4_ | 44 mV | 86 mV·dec^−1^ | Wen et al., *ACS Appl. Energy Mater.*, **2018**, 1, 3835. |
|  |  | 1.0 M KOH | 81 mV | 102 mV·dec^−1^ |  |
|  |  | 1.0 M PBS | 137 mV | 144 mV·dec^−1^ |  |
| 511 | O,Cu–CoP-2*^iR^* | 1.0 M KOH | 72 mV | 57.6 mV·dec^−1^ | Xu et al., *ACS Energy Lett.*, **2018**, 3, 2750. |
| 512 | f-CoP/CoP_2_/Al_2_O_3_ | 1.0 M KOH | 138 mV | 73 mV·dec^−1^ | Li et al., *Nanoscale*, **2017**, 9, 5677. |
| 513 | Mn-CoP nanosheets*^iR^* | 0.5 M H_2_SO_4_ | 148 mV | 61 mV·dec^−1^ | Li et al., *Dalton Trans.*, **2018**, 47, 14679. |
|  |  | 1.0 M KOH | 195 mV | 85 mV·dec^−1^ |  |
| 514 | Ultrathin CoP nanosheets*^iR^* | 1.0 M KOH | 111 mV | 70.9 mV·dec^−1^ | Chang et al., *Green Chem.*, **2016**, 18, 2287. |
| 515 | Ce-CoP@CC*^iR^* | 1.0 M KOH | 81 mV | 68.7 mV·dec^−1^ | Li et al, *Adv. Energy Mater.*, **2023**, 13, 2301162. |
| 516 | CoP@CC*^iR^* |  | 126 mV | 83.3 mV·dec^−1^ |  |
| 517 | YS-CoO/CoP*^iR^* | 1.0 M KOH | 126 mV | 65 mV·dec^−1^ | Kim et al., *J. Mater. Chem. A*, **2025**, 13, 13763. |
| 518 | CoP/PNC*^iR^* | 0.5 M H_2_SO_4_ | 99 mV | 46 mV·dec^−1^ | Peng et al., *Carbon*, **2019**, 144, 464. |
|  |  | 1.0 M KOH | 165 mV | 70 mV·dec^−1^ |  |
| 519 | Mn-CoP*^iR^* | 0.5 M H_2_SO_4_ | 108 mV | 53 mV·dec^−1^ | Li et al., *Catal. Sci. Technol.*, **2018**, 8, 4407. |
|  |  | 1.0 M KOH | 95 mV | − |  |
| 520 | b-CoP/Ni_2_P/NF*^iR^* | 1.0 M KOH | 105 mV | 99.13 mV·dec^−1^ | Chen et al., *Adv. Compos. Hybrid Mater.*, **2024**, 7, 71. |
| 521 | CoP@CoP@(Co/Ni)_2_P*^iR^* | 1.0 M KOH | 147 mV | 72.72 mV·dec^−1^ | Zhang et al., *Chem. Eng. J.*, **2023**, 463, 142448. |
|  |  | 0.5 M H_2_SO_4_ | 139 mV | 70.83 mV·dec^−1^ |  |
|  |  | 1.0 M PBS | 144 mV | 108.60 mV·dec^−1^ |  |
| 522 | CoP@WC/CC*^NiR^* | 0.5 M H_2_SO_4_ | 52 mV | 48.27 mV·dec^−1^ | He et al., *Surf. Interfaces*, **2024**, 53, 105043. |
|  |  | 1.0 M KOH | 70 mV | 56.08 mV·dec^−1^ |  |
| 523 | CoP/CC*^NiR^* | 0.5 M H_2_SO_4_ | 78 mV | 59.59 mV·dec^−1^ |  |
|  |  | 1.0 M KOH | 100 mV | 57.04 mV·dec^−1^ |  |
| 524 | Co_2_P@CoP/PC*^iR^* | 0.5 M H_2_SO_4_ | 129 mV | 50.8 mV·dec^−1^ | Lin et al., *ChemistrySelect*, **2020**, 5, 7338. |
|  |  | 1.0 M KOH | 212 mV | 74.1 mV·dec^−1^ |  |
| 525 | CoP@PNC*^NiR^* | 0.5 M H_2_SO_4_ | 84 mV | 57 mV·dec^−1^ | Zhou et al., *J. Energy Chem.*, **2017**, 26, 1223. |
|  |  | 1.0 M KOH | 120 mV | 66 mV·dec^−1^ |  |
| 526 | CoP/Mo_2_CT_x_ | 1.0 M KOH | 78 mV | 66 mV·dec^−1^ | Liu et al., *J. Mater. Chem. A*, **2021**, 9, 21259. |
| 527 | NC-CNT/CoP*^iR^* | 1.0 M KOH | 120 mV | 73 mV·dec^−1^ | Guan et al., *J. Mater. Chem. A*, **2018**, 6, 9009. |
|  |  | 0.5 M H_2_SO_4_ | 62 mV | 39 mV·dec^−1^ |  |
|  |  | 1.0 M PBS | 45 mV | 77 mV·dec^−1^ |  |
| 528 | FeP_4_/CoP*^NiR^* | 0.5 M H_2_SO_4_ | 37 mV | 45 mV·dec^−1^ | Mo et al., *Int. J. Hydrogen Energy*, **2023**, 48, 31101. |
|  |  | 1.0 M PBS | 61 mV | 70.8 mV·dec^−1^ |  |
|  |  | 1.0 M KOH | 52 mV | 81.0 mV·dec^−1^ |  |
| 529 | Zn_0.64_-CoP/Co_2_P@CC | 0.5 M H_2_SO_4_ | 81 mV | 69 mV·dec^−1^ | Yang et al., *J. Solid State Chem.*, **2020**, 285, 121231. |
|  |  | 1.0 M KOH | 95 mV | 87 mV·dec^−1^ |  |
| 530 | ECP-1*^iR^* | 1.0 M KOH | 173 mV | 69.5 mV·dec^−1^ | Xie et al., *J. Colloid Interface Sci.*, **2022**, 614, 84. |
| 531 | Ni-CoP@NC | 1.0 M KOH | 13 mV | 72.5 mV·dec^−1^ | Yan et al., *Inorg. Chem. Front.*, **2024**, 11, 6376. |
| 532 | CoP/Co_3_O_4_/CC | 1.0 M KOH | 58 mV | 116 mV·dec^−1^ | Lu et al., *ACS Appl. Nano Mater.*, **2025**, 8, 4899. |
| 533 | CoP/CC |  | 110 mV | 240 mV·dec^−1^ |  |
| 534 | CoP/Ni_2_P@NF*^iR^* | 1.0 M KOH | − | 77 mV·dec^−1^ | Liu et al., *Nano Res.*, **2024**, 17, 4797. |
| 535 | CoP*^iR^* |  | − | 138.6 mV·dec^−1^ |  |
| 536 | CC@NiCoP/CoP/Co_3_O_4_ | 0.5 M H_2_SO_4_ | 90 mV | 81.7 mV·dec^−1^ | Wu et al., *Int. J. Hydrogen Energy*, **2023**, 48, 12958. |
| 537 | FN-CoP NS*^iR^* | 1.0 M KOH | 66 mV | 44.9 mV·dec^−1^ | Xu et al., *J. Colloid Interface Sci.*, **2022**, 619, 298. |
| 538 | CoP@N,P-CNSs*^iR^* | 1.0 M KOH | 115 mV | 74.2 mV·dec^−1^ | Feng et al., *Chem. Eur. J.*, **2021**, 27, 9850. |
| 539 | CoP/MOL/NF-10*^NiR^* | 1.0 M KOH | 87 mV | 65 mV·dec^−1^ | Tao et al., *Appl. Surf. Sci.*, **2019**, 493, 852. |
| 540 | CoP-400*^iR^* | 0.5 M H_2_SO_4_ | 113 mV | 67 mV·dec^−1^ | Li et al., *Small*, **2018**, 14, 1802824. |
|  |  | 1.0 M KOH | 154 mV | 72 mV·dec^−1^ |  |
|  |  | 1.0 M PBS | 161 mV | 81 mV·dec^−1^ |  |
| 541 | CoP–MoO_2_/MF | 1.0 M KOH | 42 mV | 127 mV·dec^−1^ | Zhao et al., *J. Mater. Chem. A*, **2020**, 8, 6732. |
|  |  | 0.5 M H_2_SO_4_ | 65 mV | 85 mV·dec^−1^ |  |
| 542 | CoP/MF | 1.0 M KOH | 149 mV | 145 mV·dec^−1^ |  |
|  |  | 0.5 M H_2_SO_4_ | 121 mV | 89 mV·dec^−1^ |  |
| 543 | CoP/MnP/Cu_3_P@CF*^iR^* | 1.0 M KOH | 146 mV | 85 mV·dec^−1^ | Liu et al., *Mater. Chem. Front.*, **2025**, 9, 953. |
| 544 | CoP@N,P-C*^iR^* | 1.0 M KOH | 113 mV | 98 mV·dec^−1^ | Sun et al., *J. Electroanal. Chem.*, **2022**, 904, 115882. |
| 545 | V-doped CoP/NF*^iR^* | 1.0 M KOH | 84.6 mV | 79.2 mV·dec^−1^ | Xue et al., *Nano Res.*, **2021**, 14, 4173. |
| 546 | E380-CoP/CC*^iR^* | 1.0 M KOH | 57 mV | 40 mV·dec^−1^ | Dai et al., *J. Electroanal. Chem.*, **2023**, 944, 117632. |
| 547 | CoP/C/Cu*^iR^* | 0.5 M H_2_SO_4_ | 95 mV | 58.41 mV·dec^−1^ | Bera et al., *Chem. Eng. J.*, **2022**, 448, 137716. |
| 548 | Zn,S-CoP NRCs/CP*^iR^* | 1.0 M PBS | 67 mV | 71.6 mV·dec^−1^ | Yan et al., *J. Mater. Chem. A*, **2019**, 7, 22453. |
| 549 | CoP/HNCNP@2 D CoP*^iR^* | 1.0 M KOH | 173 mV | 108.7 mV·dec^−1^ | Lv et al., *ChemSusChem*, **2020**, 13, 3495. |
| 550 | 15Mn-CoP | 0.5 M H_2_SO_4_ | 65 mV | 32 mV·dec^−1^ | Ge et al., *ACS Sustainable Chem. Eng.*, **2018**, 6, 15162. |
|  |  | 1.0 M KOH | 100 mV | 53 mV·dec^−1^ |  |
| 551 | Ru-CoP*^iR^* | 1.0 M KOH | 47 mV | 42.1 mV·dec^−1^ | Wang et al., *Fuel*, **2023**, 334, 126635. |
|  |  | 0.5 M H_2_SO_4_ | 80 mV | 57.5 mV·dec^−1^ |  |
|  |  | 1.0 M PBS | 107 mV | 76.7 mV·dec^−1^ |  |
| 552 | CoP/Co_2_P@ACNF-800 | 1.0 M KOH | 155 mV | 88.6 mV·dec^−1^ | Chen et al., *Electrochim. Acta*, **2022**, 404, 139578. |
|  |  | 0.5 M H_2_SO_4_ | 133 mV | 53.5 mV·dec^−1^ |  |
| 553 | Ni-doped CoP/Co_2_P NSs*^iR^* | 0.5 M H_2_SO_4_ | 100 mV | 66 mV·dec^−1^ | Chen et al., *J. Solid State Chem.*, **2021**, 301, 122299. |
|  |  | 1.0 M KOH | 125 mV | 73 mV·dec^−1^ |  |
| 554 | Ni_2_P/CoP/FeP_4_/IF*^iR^* | 1.0 M KOH | − | 59.6 mV·dec^−1^ | Zhao et al., *Chin. J. Catal.*, **2024**, 61, 269. |
| 555 | Hole-rich CoP nanosheets | 0.5 M H_2_SO_4_ | 84 mV | 61 mV·dec^−1^ | Geng et al., *J. Mater. Chem. A*, **2021**, 9, 8561. |
|  |  | 1.0 M KOH | 94 mV | 67 mV·dec^−1^ |  |
| 556 | CoP/BN-C*^iR^* | 1.0 M KOH | 154 mV | 106 mV·dec^−1^ | Zhu et al., *Electrochim. Acta*, **2022**, 406, 139846. |
| 557 | N,Cu-CoP/CC*^iR^* | 1.0 M PBS | 64.7 mV | 117.3 mV·dec^−1^ | Xu et al., *Adv. Mater.*, **2022**, 34, 2200058. |
| 558 | Ce, N-CoP/CC | 0.5 M H_2_SO_4_ | 66 mV | 53 mV·dec^−1^ | Liu et al., *Sustain. Energy Fuels*, **2019**, 3, 3344. |
|  |  | 1.0 M PBS | 72 mV | 56 mV·dec^−1^ |  |
|  |  | 1.0 M KOH | 41 mV | 43 mV·dec^−1^ |  |
| 559 | CoP-MoP/NC*^iR^* | 0.5 M H_2_SO_4_ | 105.2 mV | 79.7 mV·dec^−1^ | Zheng et al., *Sol. Energy Mater. Sol. Cells*, **2024**, 269, 112794. |
| 560 | p-CoP/NF*^iR^* | 1.0 M KOH | 35 mV | 71 mV·dec^−1^ | Du et al., *New J. Chem.*, **2022**, 46, 13117. |
| 561 | Al-CoP/CC*^iR^* | 0.5 M H_2_SO_4_ | 23 mV | 43 mV·dec^−1^ | Zhang et al., *Nanoscale*, **2017**, 9, 4793. |
|  |  | 1.0 M KOH | 38 mV | 45 mV·dec^−1^ |  |
| 562 | CoP/CFP-H*^iR^* | 0.5 M H_2_SO_4_ | 128.1 mV | 49.7 mV·dec^−1^ | Yu et al., *ACS Appl. Mater. Interfaces*, **2018**, 10, 14777. |
| 563 | Ni_2_P/NiMoO_4_@CoP/FeP_2_ | 1.0 M KOH | 105 mV | 74.4 mV·dec^−1^ | Wu et al., *J. Alloys Compd.*, **2023**, 945, 169357. |
| 564 | CoP/NCNT-CP hybrid*^iR^* | 1.0 M KOH | 165 mV | 96 mV·dec^−1^ | Wang et al., *ACS Sustainable Chem. Eng.*, **2019**, 7, 10044. |
|  |  | 0.5 M H_2_SO_4_ | 203 mV | 56 mV·dec^−1^ |  |
|  |  | 1.0 M PBS | 305 mV | 100 mV·dec^−1^ |  |
| 565 | CoP/a-CoMoP/NF*^iR^* | 1.0 M KOH | − | 97.3 mV·dec^−1^ | Ma et al., *Int. J. Hydrogen Energy*, **2024**, 51, 479. |
| 566 | CeO_x_/CoP/NF-3 | 1.0 M KOH | 117 mV | 97 mV·dec^−1^ | Zhang et al., *ChemNanoMat*, 2020, 6, 1119. |
| 567 | V–CoP NRs/CC*^iR^* | 1.0 M KOH | 63 mV | 55.2 mV·dec^−1^ | Du et al., *Int. J. Hydrogen Energy*, **2021**, 46, 599. |
| 568 | O-CoP-3 nanorods*^iR^* | 1.0 M KOH | 116 mV | 59 mV·dec^−1^ | Ma et al., *Nanoscale*, **2020**, 12, 14733. |
| 569 | CoP*^iR^* | 0.5 M H_2_SO_4_ | − | 96.2 mV·dec^−1^ | Li et al., *Nat. Commun.*, **2021**, 12, 3502. |
| 570 | Co_0.6_Fe_0.4_P/CNT*^iR^* | 0.5 M H_2_SO_4_ | 67 mV | 57 mV·dec^−1^ | Zhang et al., *Adv. Funct. Mater.*, **2017**, 27, 1606635. |
|  |  | 1.0 M PBS | 105 mV | 80 mV·dec^−1^ |  |
| 571 | CoP/CNT*^iR^* | 1.0 M KOH | 76 mV | − |  |
| 572 | Ru-Co_3_O_4_/CoP/TM*^iR^* | 1.0 M KOH | 47 mV | 93 mV·dec^−1^ | Zhang et al., *Mol. Catal.*, **2022**, 530, 112640. |
| 573 | NC/Co/CoP/CP*^iR^* | 1.0 M KOH | 208 mV | 126 mV·dec^−1^ | Cong et al., *Chin. J. Catal.*, **2020**, 41, 242. |
| 574 | CoP/Ni_2_P/MoS_2_–CC*^iR^* | 1.0 M PBS | − | 63 mV·dec^−1^ | Cheng et al., *ACS Appl. Energy Mater.*, **2023**, 6, 9577. |
|  |  | 1.0 M KOH | − | 62 mV·dec^−1^ |  |
|  |  | 0.5 M H_2_SO_4_ | − | 82 mV·dec^−1^ |  |
| 575 | Cr-CoP/CP*^iR^* | 0.5 M H_2_SO_4_ | 47 mV | 46 mV·dec^−1^ | Li et al., *Chem. Eng. J.*, **2021**, 425, 130651. |
|  |  | 1.0 M PBS | 131 mV | 67 mV·dec^−1^ |  |
|  |  | 1.0 M KOH | 67 mV | 31 mV·dec^−1^ |  |
| 576 | F-CoP-Vp-2*^iR^* | 1.0 M PBS | 108 mV | 88.9 mV·dec^−1^ | Xu et al., *ACS Materials Lett.*, **2020**, 2, 736. |
|  |  | 1.0 M KOH | − | 81.2 mV·dec^−1^ |  |
| 577 | V-CPNA/CC*^iR^* | 1.0 M KOH | 87 mV | 65.4 mV·dec^−1^ | Wang et al., *ACS Appl. Energy Mater.*, **2020**, 3, 1027. |
|  |  | 0.5 M H_2_SO_4_ | 93 mV | 71.5 mV·dec^−1^ |  |
|  |  | 1.0 M PBS | 98 mV | 83.7 mV·dec^−1^ |  |
| 578 | CoP NPs/CNSs*^iR^* | 1.0 M KOH | 115 mV | 92.4 mV·dec^−1^ | Zou et al., *RSC Adv.*, **2019**, 9, 39951. |
| 579 | A-Fe-CoP/CPN*^iR^* | 1.0 M KOH | 110 mV | 101 mV·dec^−1^ | Xu et al., *J. Energy Chem.*, **2022**, 71, 36. |
| 580 | Fe-CoP/CPN*^iR^* |  | 117 mV | 107 mV·dec^−1^ |  |
| 581 | CoP/CNT/Ni_2_P*^iR^* | 1.0 M KOH | 61 mV | 49.26 mV·dec^−1^ | Yu et al., *Fuel*, **2025**, 382, 133761. |
| 582 | CoP*^iR^* |  | 88 mV | 107.83 mV·dec^−1^ |  |
| 583 | CoP/GA*^iR^* | 0.5 M H_2_SO_4_ | 121 mV | 50 mV·dec^−1^ | Zhang et al., *ChemSusChem*, **2016**, 9, 3049. |
| 584 | Cu@CoP/Cu*^NiR^* | 0.5 M H_2_SO_4_ | 98 mV | 48.5 mV·dec^−1^ | Wang et al., *Energy Fuels*, **2020**, 34, 10276. |
|  |  | 1.0 M KOH | 88 mV | 66.1 mV·dec^−1^ |  |
| 585 | CoP/Cu*^NiR^* | 0.5 M H_2_SO_4_ | 109 mV | 57.7 mV·dec^−1^ |  |
|  |  | 1.0 M KOH | − | 83.8 mV·dec^−1^ |  |
| 586 | CoP/MoSe_2_–3*^iR^* | 0.5 M H_2_SO_4_ | 135 mV | 67.5 mV·dec^−1^ | Ding et al., *Int. J. Hydrogen Energy*, **2018**, 43, 20346. |
| 587 | Ir@Zr–CoP | 1.0 M KOH | 74 mV | 77.1 mV·dec^−1^ | Ngo et al., *Adv. Energy Mater.*, **2023**, 13, 2301841. |
| 588 | CoP/CoO PNTs*^iR^* | 1.0 M KOH | 61 mV | 78 mV·dec^−1^ | Zhou et al., *Carbon Energy*, **2023**, 5, e273. |
| 589 | HP-CoP NA/NF*^iR^* | 1.0 M KOH | 70 mV | 83.2 mV·dec^−1^ | Wang et al., *Electrochim. Acta*, **2018**, 284, 504. |
| 590 | CoP NA/NF*^iR^* |  | 108 mV | 93.6 mV·dec^−1^ |  |
| 591 | Mn-CoP PMFs/CC*^iR^* | 0.5 M H_2_SO_4_ | 28 mV | 47.3 mV·dec^−1^ | Xu et al., *J. Colloid Interface Sci.*, **2020**, 577, 379. |
|  |  | 1.0 M KOH | 90 mV | 86.1 mV·dec^−1^ |  |
| 592 | s-CoP/Co_3_O_4_ | 1.0 M KOH | 106 mV | 51.2 mV·dec^−1^ | Li et al., *Nanoscale*, **2021**, 13, 20281. |
| 593 | CoP/CeO_2_–FeO_x_H*^NiR^* | 1.0 M KOH | 37 mV | 27.8 mV·dec^−1^ | Tan et al., *ACS Sustainable Chem. Eng.*, **2021**, 9, 11981. |
|  |  | 1.0 M PBS | 149 mV | 101 mV·dec^−1^ |  |
| 594 | F-CoP/Ni_2_P/NF | 1.0 M KOH | 99 mV | 76 mV·dec^−1^ | Yang et al., *J. Mater. Sci.*, **2023**, 58, 6407. |
|  |  | 0.5 M H_2_SO_4_ | 106 mV | 44 mV·dec^−1^ |  |
| 595 | Co_2_(P_2_O_7_)-CoP/NC*^iR^* | 0.5 M H_2_SO_4_ | 88 mV | 51 mV·dec^−1^ | Mo et al., *Nanotechnology*, **2019**, 30, 394001. |
|  |  | 1.0 M KOH | 97 mV | 61 mV·dec^−1^ |  |
| 596 | CoP/Cu_2_O@CF | 1.0 M KOH | 62 mV | 83 mV·dec^−1^ | Qi et al., *Front. Chem. Sci. Eng.*, **2023**, 17, 1430. |
| 597 | NiCoP@SSM*^iR^* | 1.0 M KOH | 138 mV | 74 mV·dec^−1^ | Gebreslase, et al., *Electrochim. Acta*, **2023**, 438, 141538. |
| 598 | CoP-A*^iR^* | 1.0 M KOH | 100 mV | 76 mV·dec^−1^ | Su et al., *Chem. Sci.*, **2019**, 10, 2019. |
| 599 | 10Ni@CoP*^iR^* | 1.0 M KOH | 188 mV | 69 mV·dec^−1^ | Abahussain et al., *Fuel*, **2024**, 368, 131605. |
| 600 | CoP-PBSCF | 1.0 M KOH | 240 mV | 93.8 mV·dec^−1^ | Zhang et al, *J. Mater. Chem. A*, **2019**, 7, 26607. |
| 601 | B-CoP/CNT*^iR^* | 0.5 M H_2_SO_4_ | 39 mV | 50 mV·dec^−1^ | Cao et al., *Angew. Chem. Int. Ed.*, **2020**, 59, 4154. |
|  |  | 1.0 M PBS | 79 mV | 80 mV·dec^−1^ |  |
|  |  | 1.0 M KOH | 56 mV | 69 mV·dec^−1^ |  |
| 602 | CoP@MoS_2_*^iR^* | 0.5 M H_2_SO_4_ | 119 mV | 49 mV·dec^−1^ | Xia et al., *Mater. Des.*, **2021**, 211, 110165. |
| 603 | CoP/Fe-Co_9_S_8_*^iR^* | 1.0 M KOH | 62 mV | − | Chen et al., *Adv. Sci.*, **2022**, 9, 2204742. |
| 604 | CoP-0.6/C*^iR^* | 1.0 M KOH | 140 mV | 109.30 mV·dec^−1^ | Li et al., *J. Alloys Compd.*, **2020**, 829, 154535. |
| 605 | PD-CoP UNSs/CC*^iR^* | 0.5 M H_2_SO_4_ | 47 mV | 46 mV·dec^−1^ | Dang et al., *ACS Appl. Mater. Interfaces*, **2019**, 11, 29879. |
|  |  | 1.0 M PBS | 90 mV | 101 mV·dec^−1^ |  |
|  |  | 1.0 M KOH | 51 mV | 53 mV·dec^−1^ |  |
| 606 | H-CoP@NC*^iR^* | 1.0 M KOH | 200 mV | 71 mV·dec^−1^ | Xie et al., *Inorg. Chem.*, **2019**, 58, 14652. |
| 607 | p-NFNR@Ni–Co–P | 1.0 M KOH | 125 mV | 85 mV·dec^−1^ | Feng et al., *ACS Appl. Mater. Interfaces*, **2021**, 13, 48949. |
| 608 | CoP/NC-2*^iR^* | 0.5 M H_2_SO_4_ | 98 mV | 52.2 mV·dec^−1^ | Zhang et al., *Surf. Interfaces*, **2024**, 47, 104224. |
|  |  | 1.0 M KOH | 139 mV | 67.3 mV·dec^−1^ |  |
| 609 | M-Mo-CoP/CF*^iR^* | 1.0 M KOH | − | 99.9 mV·dec^−1^ | Li et al., 2023, *Electrochim. Acta*, **2023**, 472, 143429. |
| 610 | CoP/RGO-PL | 0.5 M H_2_SO_4_ | 168 mV | 57 mV·dec^−1^ | Wang et al., *Electrochim. Acta*, **2018**, 284, 534. |
| 611 | Fe-CoP/Ti*^iR^* | 1.0 M KOH | 78 mV | 75 mV·dec^−1^ | Tang et al., *Adv. Mater.*, **2017**, 29, 1602441. |
| 612 | CoP/Ti*^iR^* |  | 128 mV | 78 mV·dec^−1^ |  |
| 613 | Mn-CoP/NF-P/NF | 1.0 M KOH | 69 mV | 84 mV·dec^−1^ | Sirisomboonchai et al., *ACS Appl. Energy Mater.*, **2022**, 5, 149. |
| 614 | CoP@N,S-3D-GN | 0.5 M H_2_SO_4_ | 118 mV | 50 mV·dec^−1^ | Karaman et al., *Electrochim. Acta*, **2021**, 380, 138262. |
| 615 | Ru-CoP/CC-2 | 1.0 M KOH | 45 mV | 98.11 mV·dec^−1^ | Cui et al., *ChemElectroChem*, **2022**, 9, e202101482. |
| 616 | CoP\|S*^NiR^* | 1.0 M KOH | 103 mV | 58 mV·dec^−1^ | Hong et al., *Appl. Catal. B Environ.*, **2019**, 251, 213. |
|  |  | 0.5 M H_2_SO_4_ | 107 mV | 57 mV·dec^−1^ |  |
| 617 | Co/CoP crystalline-amorphous hetero-phase*^iR^* | 1.0 M KOH | 83 mV | 55.3 mV·dec^−1^ | Wu et al., *Chem. Commun.*, **2023**, 59, 2429. |
| 618 | Mn-Co(OH)_2_/R-Mn-CoP composite | 1.0 M KOH | 110 mV | 45 mV·dec^−1^ | Li et al., *Appl. Catal. B Environ.*, **2021**, 292, 120172. |
| 619 | CoP/Co_2_P@NC-2*^iR^* | 0.5 M H_2_SO_4_ | 126 mV | 79 mV·dec^−1^ | Lv et al., *ACS Sustainable Chem. Eng.*, **2019**, 7, 8993. |
|  |  | 1.0 M KOH | 198 mV | 82 mV·dec^−1^ |  |
|  |  | 1.0 M PBS | 459 mV | − |  |
| 620 | CoP-PNC2*^NiR^* | 1.0 M KOH | 106 mV | 84 mV·dec^−1^ | Boppella et al., *Carbon*, **2020**, 156, 529. |
|  |  | 0.5 M H_2_SO_4_ | 87 mV | 58 mV·dec^−1^ |  |
| 621 | I_SA_-CoP_n_/CoP*^iR^* | 0.5 M H_2_SO_4_ | 44 mV | 41.2 mV·dec^−1^ | Wang et al., *Small*, **2024**, 20, 2403170. |
| 622 | Triphasic CoP@Cu_2_P-Cu_3_P*^iR^* | 1.0 M KOH | 188 mV | 89 mV·dec^−1^ | Saad et al., *Sustain. Energy Fuels*, **2021**, 5, 1366. |
| 623 | CoP/Ni_5_P_4_/CoP*^iR^* | 0.5 M H_2_SO_4_ | 33 mV | 43 mV·dec^−1^ | Mishra et al., *Energy Environ. Sci.*, **2018**, 11, 2246. |
|  |  | 1.0 M KOH | 71 mV | 58 mV·dec^−1^ |  |
| 624 | Mn−(Co,Ni)_2_P/CoP/(N,S)−C*^iR^* | 1.0 M KOH | 61 mV | 58 mV·dec^−1^ | Kandel et al., *Small*, **2024**, 20, 2307241. |
| 625 | F-CoP/CF*^iR^* | 1.0 M KOH | − | 28 mV·dec^−1^ | Li et al., *Mater. Horiz.*, **2023**, 10, 5277. |
| 626 | MoS_2_/S-CoP | 1.0 M KOH | 78 mV | 57.54 mV·dec^−1^ | Li et al., *Ceram. Int.*, **2024**, 50, 18519. |
|  |  | 0.5 M H_2_SO_4_ | 63 mV | 46.58 mV·dec^−1^ |  |
| 627 | CoP | 1.0 M KOH | 121 mV | 74.49 mV·dec^−1^ |  |
|  |  | 0.5 M H_2_SO_4_ | 96 mV | 64.27 mV·dec^−1^ |  |
| 628 | S-CoP | 1.0 M KOH | 114 mV | 63.31 mV·dec^−1^ |  |
|  |  | 0.5 M H_2_SO_4_ | 84 mV | 54.03 mV·dec^−1^ |  |
| 629 | CoP(0.1)@3DOM-FeP | 1.0 M KOH | 67.2 mV | 66.3 mV·dec^−1^ | Wu et al., *J. Mater. Chem. A*, **2021**, 9, 23574. |
|  |  | 1.0 M PBS | 71.1 mV | 76.0 mV·dec^−1^ |  |
|  |  | 0.5 M H_2_SO_4_ | 76.5 mV | 77.5 mV·dec^−1^ |  |
| 630 | CoH-CoPV@CFP*^iR^* | 1.0 M KOH | 77 mV | 75.2 mV·dec^−1^ | Wei et al., *Appl. Catal. B Environ. Energy*, **2024**, 345, 123661. |
| 631 | F-CDs/CoP/NF*^iR^* | 1.0 M KOH | 16 mV | 89 mV·dec^−1^ | Song et al., *Adv. Energy Mater.*, **2022**, 12, 2102573. |
| 632 | Ru–CoP/CC*^iR^* | 1.0 M KOH | 21 mV | 49 mV·dec^−1^ | Xiao et al., *Mater. Today Phys.*, **2020**, 12, 100182. |
| 633 | CoP/NPCFs*^iR^* | 0.5 M H_2_SO_4_ | 135 mV | 67 mV·dec^−1^ | Lin et al., *Electrochim. Acta*, **2017**, 232, 561. |
| 634 | N-Ni_5_P_4_@CoP/CFP*^iR^* | 0.5 M H_2_SO_4_ | 55 mV | 46.6 mV·dec^−1^ | Zhang et al., *Appl. Catal. B Environ.*, **2023**, 122207. |
|  |  | 1.0 M KOH | 56 mV | 63.0 mV·dec^−1^ |  |
|  |  | 1.0 M PBS | 59 mV | 81.2 mV·dec^−1^ |  |
| 635 | u-CoP/Ti*^iR^* | 0.5 M H_2_SO_4_ | 45 mV | 49.3 mV·dec^−1^ | Zhou et al., *J. Mater. Chem. A*, **2016**, 4, 10114. |
|  |  | 1.0 M KOH | 60 mV | 49.1 mV·dec^−1^ |  |
| 636 | Co_0.67_V_0.33_P@CC*^iR^* | 1.0 M KOH | 77 mV | 55.59 mV·dec^−1^ | Han et al., *J. Alloys Compd.*, **2020**, 846, 156350. |
| 637 | CoP@FeCoP/NC YSMPs*^iR^* | 1.0 M KOH | 141 mV | 56.34 mV·dec^−1^ | Shi et al., *Chem. Eng. J.*, **2021**, 403, 126312. |
| 638 | Co/CeO_2_/Co_2_P/CoP@NC*^NiR^* | 1.0 M KOH | 195 mV | 66.0 mV·dec^−1^ | Song et al., *Int. J. Hydrogen Energy*, **2020**, 45, 30559. |
| 639 | CoP NA/CC*^iR^* | 1.0 M PBS | 145 mV | 123 mV·dec^−1^ | Liu et al., *ChemElectroChem*, **2017**, 4, 1840. |
|  |  | 1.0 M KOH | 52 mV | 60 mV·dec^−1^ |  |
| 640 | CoP/MoP@NC/CC*^iR^* | 1.0 M KOH | 94 mV | 40 mV·dec^−1^ | Tang et al., *Appl. Catal. B Environ.*, **2019**, 245, 528. |
| 641 | CoP@NC*^iR^* |  | 157 mV | 85 mV·dec^−1^ |  |
| 642 | CoP/Ni_2_P@NHPC*^iR^* | 1.0 M KOH | 159 mV | 62.58 mV·dec^−1^ | Zhang et al., *J. Electroanal. Chem.*, **2024**, 961, 118224. |
| 643 | CoP/Co_3_O_4_@TM-3*^iR^* | 1.0 M KOH | 98 mV | 53.6 mV·dec^−1^ | Tian et al., *Chem. Eur. J.*, **2023**, 29, e202301478. |
| 644 | CoP@TM*^iR^* |  | 72 mV | 41.8 mV·dec^−1^ |  |
| 645 | MoSe_2_/CoP hybrids*^iR^* | 0.5 M H_2_SO_4_ | 105 mV | 51 mV·dec^−1^ | Zhang et al., *Int. J. Hydrogen Energy*, **2020**, 45, 19246. |
| 646 | CoP*^iR^* |  | 189 mV | 123 mV·dec^−1^ |  |
| 647 | CoP/R-NF*^iR^* | 1.0 M KOH | 35 mV | 70 mV·dec^−1^ | Zhou et al., *J. Colloid Interface Sci.*, **2025**, 683, 509. |
| 648 | CoMoO_4_-CoP/NC*^iR^* | 1.0 M KOH | 122 mV | 68.22 mV·dec^−1^ | Luo et al., *J. Colloid Interface Sci.*, **2023**, 648, 90. |
| 649 | MC-V-CoP hollow nanofiber*^iR^* | 1.0 M KOH | 65 mV | 72.5 mV·dec^−1^ | Zhu et al., *Nanoscale*, **2020**, 12, 9144. |
|  |  | 1.0 M PBS | 112 mV | 91.7 mV·dec^−1^ |  |
| 650 | V_p_−CoP−FeP*^iR^* | 1.0 M KOH | 58 mV | 70.6 mV·dec^−1^ | Qi et al., *Chem. Eur. J.*, **2023**, 29, e202301521. |
| 651 | CoP@CNFs-400*^iR^* | 1.0 M KOH | 85 mV | 69.9 mV·dec^−1^ | Zhu et al., *J. Alloys Compd.*, **2023**, 938, 168674. |
| 652 | 3LH-Co/CoP@NC/CC*^iR^* | 0.5 M H_2_SO_4_ | 86 mV | 84 mV·dec^−1^ | Wang et al., *Sci. China Chem.*, **2022**, 65, 619. |
|  |  | 1.0 M KOH | 78 mV | 145 mV·dec^−1^ |  |
|  |  | 1.0 M PBS | 145 mV | 129 mV·dec^−1^ |  |
| 653 | CoP@NPC-800*^iR^* | 0.5 M H_2_SO_4_ | 81 mV | 74.1 mV·dec^−1^ | Chen et al., *Sci. China Mater.*, **2022**, 65, 2433. |
|  |  | 1.0 M KOH | 109 mV | 78.9 mV·dec^−1^ |  |
|  |  | 1.0 M PBS | 227 mV | 190.1 mV·dec^−1^ |  |
| 654 | La–CoP*^iR^* | 1.0 M KOH | 109 mV | 65.3 mV·dec^−1^ | Kim et al., *CrystEngComm*, **2022**, 24, 7283. |
| 655 | CoP*^iR^* |  | 142 mV | 90.1 mV·dec^−1^ |  |
| 656 | V, N-CoP | 1.0 M KOH | 57 mV | 51 mV·dec^−1^ | Zhang et al., *J. Alloys Compd.*, **2019**, 791, 1070. |
|  |  | 1.0 M PBS | 146 mV | 88 mV·dec^−1^ |  |
|  |  | 0.5 M H_2_SO_4_ | 81 mV | 59 mV·dec^−1^ |  |
| 657 | CoP/CC | 1.0 M KOH | 107 mV | 87 mV·dec^−1^ |  |
| 658 | V-CoP/CC |  | 92 mV | 65 mV·dec^−1^ |  |
| 659 | OMS-Ni_1_-CoP*^iR^* | 1.0 M KOH | 119 mV | 74.6 mV·dec^−1^ | Wang et al., *Appl. Catal. B Environ.*, **2022**, 316, 121667. |
| 660 | CoP NW/Hb*^NiR^* | 1.0 M PBS | 121 mV | 106 mV·dec^−1^ | Huang et al., *Nano Res.*, **2017**, 10, 1010. |
|  |  | 0.5 M H_2_SO_4_ | 78 mV | 68 mV·dec^−1^ |  |
|  |  | 1.0 M KOH | 52 mV | 88 mV·dec^−1^ |  |
| 661 | CoP@NC-Ru*^iR^* | 1.0 M KOH | 35 mV | 72 mV·dec^−1^ | Kumar et al., *ACS Appl. Mater. Interfaces*, **2024**, 16, 3542. |
| 662 | 4.3%B-CoP/NC*^iR^* | 1.0 M KOH | 158 mV | 67 mV·dec^−1^ | Fu et al., *New J. Chem.*, **2023**, 47, 17333. |
| 663 | B-CoP*^iR^* | 1.0 M KOH | − | 61.9 mV·dec^−1^ | Gao et al., *Appl. Catal. B Environ.*, **2023**, 320, 122014. |
| 664 | CoP*^iR^* |  | − | 70.3 mV·dec^−1^ |  |
| 665 | V-CoP/CC*^iR^* | 1.0 M KOH | 71 mV | 67.6 mV·dec^−1^ | Xiao et al., *Chem. Sci.*, **2018**, 9, 1970. |
|  |  | 1.0 M PBS | 123 mV | 72.6 mV·dec^−1^ |  |
|  |  | 0.5 M H_2_SO_4_ | 47 mV | 54.9 mV·dec^−1^ |  |
| 666 | CoP/CC*^iR^* | 1.0 M KOH | 112 mV | 74.8 mV·dec^−1^ |  |
| 667 | YS-Pt-CoP polyhedra*^iR^* | 1.0 M KOH | 48 mV | 54 mV·dec^−1^ | Jiang et al., *Dalton Trans.*, **2019**, 48, 8920. |
|  |  | 1.0 M PBS | 88 mV | 74 mV·dec^−1^ |  |
| 668 | CoP/PNF*^iR^* | 1.0 M KOH | − | 62.8 mV·dec^−1^ | Liao et al., *J. Mater. Chem. A*, **2024**, 12, 9830. |
| 669 | g-CoP/NF VDNCs*^iR^* | 0.5 M H_2_SO_4_ | 71 mV | 46.7 mV·dec^−1^ | Ge et al., *Int. J. Hydrogen Energy*, **2019**, 44, 13490. |
|  |  | 1.0 M KOH | 117 mV | 63.0 mV·dec^−1^ |  |
| 670 | Mn–CoP/MXene*^iR^* | 1.0 M KOH | 95 mV | 54.1 mV·dec^−1^ | Guo et al., *Appl. Phys. Lett.*, **2023**, 123, 063903. |
| 671 | CoP | 1.0 M KOH | 65.6 mV | 61.2 mV·dec^−1^ | Zhou et al., *Appl. Surf. Sci.*, **2025**, 689, 162535. |
| 672 | CoP@NPC-900*^NiR^* | 0.5 M H_2_SO_4_ | 181 mV | 59 mV·dec^−1^ | Ma et al., *J. Mater. Sci.*, **2021**, 56, 18188. |
| 673 | CoP MBs*^NiR^* | 0.5 M H_2_SO_4_ | 105.3 mV | 53.5 mV·dec^−1^ | Cao et al., *Electrochim. Acta*, **2018**, 259, 830. |
|  |  | 1.0 M KOH | 97.3 mV | 84.3 mV·dec^−1^ |  |
| 674 | NC@FeP_4_-CoP/NF*^iR^* | 1.0 M KOH | 116 mV | 53.7 mV·dec^−1^ | Yan et al., *Adv. Mater. Interfaces*, **2021**, 8, 2100065. |
| 675 | OMCs/CoP | 0.5 M H_2_SO_4_ | 134 mV | 52 mV·dec^−1^ | Xu et al., *Electrochim. Acta*, **2018**, 261, 412. |
|  |  | 1.0 M KOH | 143 mV | 58 mV·dec^−1^ |  |
| 676 | Mo-CoP/NC*^iR^* | 1.0 M KOH | − | 35.2 mV·dec^−1^ | An et al., *ACS Catal.*, **2025**, 15, 10358. |
| 677 | Mo-CoP*^iR^* |  | − | 71.9 mV·dec^−1^ |  |
| 678 | CoP/NC*^iR^* |  | − | 72.5 mV·dec^−1^ |  |
| 679 | CoP*^iR^* |  | − | 88.3 mV·dec^−1^ |  |
| 680 | B,N,S-CoP@C@rGO | 1.0 M KOH | 112 mV | 76 mV·dec^−1^ | Xu et al., *Appl. Catal. B Environ.*, **2020**, 268, 118404. |
| 681 | B,N-CoP@C@rGO |  | 143 mV | 88 mV·dec^−1^ |  |
| 682 | CoP@NC/rGO*^iR^* | 0.5 M H_2_SO_4_ | 123.8 mV | 48.3 mV·dec^−1^ | Zhao et al., *Nano Res.*, **2019**, 12, 2872. |
| 683 | Cu-CoP | 1.0 M KOH | 170 mV | 88 mV·dec^−1^ | Zou et al., *Sci. China Mater.*, **2023**, 66, 3139. |
| 684 | CoP NFs |  | 186 mV | − |  |
| 685 | CoP@NC/GR*^iR^* | 0.5 M H_2_SO_4_ | 105 mV | 47.5 mV·dec^−1^ | Guo et al., *Inorg. Chem.*, **2021**, 60, 16761. |
|  |  | 1.0 M KOH | 125 mV | 60.5 mV·dec^−1^ |  |
| 686 | CoWCP-NPC-2:1 | 1.0 M KOH | 139 mV | 116.9 mV·dec^−1^ | Zhang et al., *Chem. Eng. J.*, **2022**, 431, 134188. |
| 687 | r-Mn–Ni/CoP*^NiR^* | 1.0 M KOH | − | 43.3 mV·dec^−1^ | Xiong et al., *Adv. Funct. Mater.*, **2024**, 34, 2402298. |
| 688 | r-CoP*^NiR^* |  | − | 52.3 mV·dec^−1^ |  |
| 689 | CoP@NSPC-900*^iR^* | 0.5 M H_2_SO_4_ | 160 mV | 87.5 mV·dec^−1^ | Wang et al., *J. Energy Inst.*, **2023**, 111, 101444. |
| 690 | S_0.5_-Cu-CoP*^iR^* | 0.5 M H_2_SO_4_ | 65 mV | 38.13 mV·dec^−1^ | Pan et al., *Fuel*, **2024**, 378, 132954. |
|  |  | 1.0 M KOH | 139 mV | 91.65 mV·dec^−1^ |  |
| 691 | CoP/SPNF*^iR^* | 1.0 M KOH | 45 mV | 65.9 mV·dec^−1^ | Cao et al., *Inorg. Chem.*, **2020**, 59, 8522. |
| 692 | Ru_1_CoP/CDs-1000*^NiR^* | 1.0 M KOH | 51 mV | 73.4 mV·dec^−1^ | Song et al., *Angew. Chem. Int. Ed.*, **2021**, 60, 7234. |
|  |  | 0.5 M H_2_SO_4_ | 49 mV | 51.6 mV·dec^−1^ |  |
| 693 | CoO/CoP/Co_2_P | 1.0 M KOH | 167 mV | 81 mV·dec^−1^ | Shen et al., *Sep. Purif. Technol.*, **2023**, 305, 122399. |
| 694 | Fe_0.14_Co_0.86_-P/CC*^iR^* | 1.0 M KOH | 130 mV | 57.2 mV·dec^−1^ | Yu et al., *J. Colloid Interface Sci.*, **2021**, 600, 811. |
| 695 | Ru_0.25_Co_0.75_P@NPC*^iR^* | 0.5 M H_2_SO_4_ | 73 mV | 46 mV·dec^−1^ | Yu et al., *Chem. Eur. J.*, **2018**, 24, 13800. |
| 696 | CoP@Ni_2_P*^iR^* | 1.0 M KOH | 16 mV | 68.37 mV·dec^−1^ | Jin et al., *Appl. Catal. B Environ.*, **2021**, 296, 120350. |
| 697 | CoP NW*^iR^* | 1.0 M KOH | − | 105 mV·dec^−1^ | Li et al., *Chem. Sci.*, **2017**, 8, 2952. |
| 698 | MoC_x_/CoP@C*^iR^* | 1.0 M KOH | 95 mV | 71 mV·dec^−1^ | Li et al., *Small*, **2025**, 21, 2500135. |
| 699 | CoP@NRGO | 0.5 M H_2_SO_4_ | 90 mV | 87 mV·dec^−1^ | Wang et al., *J. Solid State Chem.*, **2020**, 290, 121596. |
| 700 | WP/CoP-FeP_4_/NF*^iR^* | 1.0 M KOH | − | 43.4 mV·dec^−1^ | Meng et al., *ACS Appl. Nano Mater.*, **2023**, 6, 16465. |
| 701 | CoO/CoP–NC*^iR^* | 1.0 M KOH | 178 mV | 88 mV·dec^−1^ | Chen et al., *J. Mater. Chem. A*, **2023**, 11, 3136. |
| 702 | 2D CoP*^iR^* | 1.0 M KOH | 144 mV | 55.5 mV·dec^−1^ | Guo et al., *Adv. Funct. Mater.*, **2022**, 32, 2209397. |
| 703 | CoP/NiCoP/Co–Ni–N–C*^iR^* | 1.0 M KOH | 40 mV | 55 mV·dec^−1^ | Liu et al., *Int. J. Hydrogen Energy*, **2024**, 56, 725. |
| 704 | CoP/NF*^iR^* | 1.0 M KOH | 153 mV | 83 mV·dec^−1^ | Reddy et al., *Int. J. Hydrogen Energy*, **2025**, 138, 215. |
| 705 | Fe-CoP/PCNF*^iR^* | 0.5 M H_2_SO_4_ | 151 mV | 53.9 mV·dec^−1^ | Chen et al., *J. Alloys Compd.*, **2022**, 918, 165733. |
| 706 | Ni_2_P/CoP*^iR^* | 0.5 M H_2_SO_4_ | 160 mV | 57 mV·dec^−1^ | Xu et al., *Appl. Surf. Sci.*, **2020**, 509, 145383. |
| 707 | CoP–CNT composite*^iR^* | 0.1 M NaOH | − | 56 mV·dec^−1^ | Hou et al., *ACS Appl. Mater. Interfaces*, **2015**, 7, 28412. |
| 708 | O,Mo−CoP/NFs-2*^iR^* | 1.0 M KOH | 59 mV | 65.4 mV·dec^−1^ | Mu et al., *ChemElectroChem*, **2021**, 8, 103. |
| 709 | CoP–Ce_2_(CO_3_)_2_O/NF*^iR^* | 1.0 M KOH | 85.2 mV | 65.2 mV·dec^−1^ | Wang et al., *Mater. Chem. Front.*, **2023**, 7, 2628. |
| 710 | Co@CoP/CNT*^iR^* | 0.5 M H_2_SO_4_ | 67 mV | 52 mV·dec^−1^ | Li et al., *J. Alloys Compd.*, **2022**, 929, 167247. |
|  |  | 1.0 M KOH | 126 mV | 80 mV·dec^−1^ |  |
| 711 | CoP/C_PDS_*^NiR^* | 1.0 M KOH | − | 79 mV·dec^−1^ | Mohite et al., *Inorg. Chem.*, **2020**, 59, 1996. |
| 712 | Mn-CP NTs*^iR^* | 1.0 M KOH | 82 mV | 86.84 mV·dec^−1^ | Tang et al., *J. Colloid Interface Sci.*, **2022**, 628, 524. |
| 713 | Cr-CoP*^iR^* | 1.0 M KOH | 36 mV | 54 mV·dec^−1^ | Men et al., *Cell Rep. Phys. Sci.*, **2020**, 1, 100136. |
| 714 | CoP*^iR^* |  | 86 mV | 73 mV·dec^−1^ |  |
| 715 | MnCoP/CC*^NiR^* | 1.0 M KOH | 69 mV | 46.16 mV·dec^−1^ | Wang et al., *Appl. Surf. Sci.*, **2020**, 515, 146059. |
| 716 | CoP/CC*^NiR^* |  | 124 mV | 60.87 mV·dec^−1^ |  |
| 717 | CoP–OMC | 0.5 M H_2_SO_4_ | 112.18 mV | 56.67 mV·dec^−1^ | Li et al., *J. Mater. Chem. A*, **2015**, 3, 4255. |
| 718 | Mo-doped CoP*^iR^* | 1.0 M KOH | 78.4 mV | 62.5 mV·dec^−1^ | Kim et al., *Chem. Eng. J.*, **2021**, 408, 127331. |
| 719 | CoP/PCNF-0.4 | 0.5 M H_2_SO_4_ | 83 mV | 62 mV·dec^−1^ | Lu et al., *Nano Res.*, **2018**, 11, 1274. |
|  |  | 1.0 M KOH | 138 mV | 81 mV·dec^−1^ |  |
|  |  | 1.0 M PBS | 191 mV | − |  |
| 720 | CoO_x_/Fe-CoP*^iR^* | 1.0 M KOH | 50 mV | 73 mV·dec^−1^ | Sun et al., *Chem. Eng. J.*, **2023**, 478, 147374. |
| 721 | WMo-CoP@NM*^NiR^* | 1.0 M KOH | − | 42.3 mV·dec^−1^ | Xu et al., *Adv. Energy Mater.*, **2025**, 2500926. |
| 722 | HMF CoP*^iR^* | 0.5 M H_2_SO_4_ | 106 mV | 44.5 mV·dec^−1^ | Guo et al., *Electrochim. Acta*, **2019**, 322, 134768. |
| 723 | W,Fe@CoP/CNTs*^iR^* | 1.0 M KOH | 130 mV | 73 mV·dec^−1^ | Ding et al., *ACS Appl. Energy Mater.*, **2019**, 2, 8022. |
| 724 | CoP-B/CNTs/CC | 1.0 M KOH | 73.8 mV | 51.5 mV·dec^−1^ | Guo et al., *J. Colloid Interface Sci.*, **2023**, 651, 172. |
| 725 | MoO_3_@CoP@rGO*^iR^* | 1.0 M KOH | 83 mV | 58.5 mV·dec^−1^ | Hou et al., *J. Mater. Sci. Technol.*, **2022**, 104, 194. |
| 726 | CoP/CNT*^iR^* | 0.5 M H_2_SO_4_ | 87 mV | − | Wu et al., *J. Phys. Chem. C*, **2018**, 122, 2848. |
|  |  | 1.0 M KOH | 114 mV | − |  |
| 727 | CoP/Co_2_P/NCNT@CF*^iR^* | 1.0 M KOH | 133 mV | 95.11 mV·dec^−1^ | Zhang et al., *ACS Omega*, **2022**, 7, 12846. |
| 728 | Mo-CoP (1/2.3) | 0.5 M H_2_SO_4_ | 116 mV | 69 mV·dec^−1^ | Li et al., *Langmuir*, **2021**, 37, 5986. |
|  |  | 1.0 M KOH | 118 mV | 76 mV·dec^−1^ |  |
| 729 | CoP–Mo_2_C@NC*^iR^* | 1.0 M KOH | 94 mV | 79.7 mV·dec^−1^ | Dutta et al., *ChemSusChem*, **2018**, 11, 3956. |
|  |  | 0.5 M H_2_SO_4_ | 107 mV | 70.8 mV·dec^−1^ |  |
| 730 | CoP/Ni_2_P*^iR^* | 0.5 M H_2_SO_4_ | 36 mV | − | Liao et al., *Mater. Today Phys.*, **2022**, 22, 100589. |
|  |  | 1.0 M PBS | 54 mV | − |  |
|  |  | 1.0 M KOH | 57 mV | − |  |
| 731 | Cu–CoP/CF*^iR^* | 0.5 M H_2_SO_4_ | 39 mV | 39.4 mV·dec^−1^ | Zhu et al., *Chem*, **2025**, 11, 102331. |
| 732 | CoP/CC*^iR^* |  | 115 mV | − |  |
| 733 | CoP-Ti_3_C_2_T_x_*^iR^* | 0.5 M H_2_SO_4_ | 135 mV | 48 mV·dec^−1^ | Sun et al., *Acta Phys. -Chim. Sin.*, **2024**, 40, 2308015. |
| 734 | NiFe-LDH@CoP-Ni_5_P_4_*^iR^* | 1.0 M KOH | 48 mV | 92.1 mV·dec^−1^ | Tan et al., *ACS Sustainable Chem. Eng.*, **2024**, 12, 5139. |
| 735 | A-Co_0.9_V_0.1_P/TF*^iR^* | 1.0 M KOH | 107 mV | 72 mV·dec^−1^ | Dong et al., *J. Mater. Chem. A*, **2022**, 10, 17477. |
| 736 | F_0.25_CP-G*^iR^* | 1.0 M KOH | 66 mV | 61 mV·dec^−1^ | Tahmasebi et al., *Dalton Trans.*, **2021**, 50, 12168. |
| 737 | Fe–CoP@NC/N-rGO | 1.0 M KOH | 84 mV | 44 mV·dec^−1^ | Amirpoor et al., *Int. J. Hydrogen Energy*, **2025**, 109, 1023. |
| 738 | NF/O-CoP*^iR^* | 1.0 M KOH | − | 57.2 mV·dec^−1^ | Dong et al., *Small Methods*, **2023**, 7, 2300071. |
| 739 | CoFe-LDH@CoP/NF*^iR^* | 1.0 M KOH | 63 mV | 54.8 mV·dec^−1^ | Song et al., *Chem. Eng. Sci.*, **2024**, 294, 120100. |
| 740 | CoP/Co_2_P_1*^iR^* | 0.5 M H_2_SO_4_ | 99 mV | 51.4 mV·dec^−1^ | Chen et al., *Nanoscale*, **2018**, 10, 21019. |
|  |  | 1.0 M KOH | 103 mV | 61.2 mV·dec^−1^ |  |
| 741 | Fe–CoP | 1.0 M KOH | 78.5 mV | 60.7 mV·dec^−1^ | Li et al., *J. Mater. Chem. A*, **2025**, 13, 7263. |
| 742 | Co_2_P&CoP@N–C*^iR^* | 1.0 M KOH | 62.8 mV | 60 mV·dec^−1^ | Yi et al., *ACS Appl. Mater. Interfaces*, **2021**, 13, 56064. |
| 743 | CoP@NPC*^iR^* | 1.0 M KOH | 184.35 mV | 69.2 mV·dec^−1^ | Sun et al., *J. Alloys Compd.*, **2023**, 932, 167253. |
| 744 | MoP/CoP/Cu_3_P/CF*^iR^* | 1.0 M KOH | 64.6 mV | 68.2 mV·dec^−1^ | Chen et al., *Mater. Horiz.*, **2023**, 10, 3761. |
| 745 | Mn_10_-CoP@CNFs*^iR^* | 1.0 M KOH | 62 mV | 87 mV·dec^−1^ | Liu et al., *Inorg. Chem. Front.*, **2025**, 12, 2792. |
| 746 | CoP@NiFe LDH/Ni*^iR^* | 1.0 M KOH | 260 mV | 71.8 mV·dec^−1^ | Liu et al., *J. Alloys Compd.*, **2024**, 973, 172886. |
| 747 | CoP@CoOOH/CP*^iR^* | 1.0 M PBS | 89.6 mV | 64.4 mV·dec^−1^ | Zhang et al., *Small*, **2022**, 18, 2106012. |
|  |  | 1.0 M KOH | 81.7 mV | 50.7 mV·dec^−1^ |  |
| 748 | CoSe_2_/a-CoP*^iR^* | 0.5 M H_2_SO_4_ | 65 mV | 54 mV·dec^−1^ | Shen et al., *Adv. Mater.*, **2022**, 34, 2110631. |
|  |  | 1.0 M KOH | 151 mV | 80 mV·dec^−1^ |  |
|  |  | 1.0 M PBS | 185 mV | 100 mV·dec^−1^ |  |
| 749 | Ru/CoP*^iR^* | 1.0 M KOH | 36 mV | 73.0 mV·dec^−1^ | Zhu et al., *Adv. Sustainable Syst.*, **2024**, 2400041. |
| 750 | CFC-CNT-CoO_x_/CoP*^iR^* | 1.0 M KOH | 108 mV | 60 mV·dec^−1^ | Zhan et al., *Chem. Eng. J.*, **2021**, 416, 128943. |
| 751 | W−CoP/CC*^iR^* | 0.5 M H_2_SO_4_ | 32 mV | 57 mV·dec^−1^ | Ren et al., *ChemElectroChem*, **2019**, 6, 5229. |
|  |  | 1.0 M KOH | 77 mV | 65 mV·dec^−1^ |  |
| 752 | Mn-CoP/NiPO*^iR^* | 1.0 M KOH | 116 mV | 47.03 mV·dec^−1^ | Liu et al., *Inorg. Chem.*, **2022**, 61, 14201. |
| 753 | CoP–Ru | 0.5 M H_2_SO_4_ | 32 mV | 37.6 mV·dec^−1^ | Wang et al., *Chin. J. Struct. Chem.*, **2023**, 42, 100035. |
| 754 | CoP |  | 56 mV | 44.9 mV·dec^−1^ |  |
| 755 | CoP/Co_2_Fe_1_P/CC*^iR^* | 1.0 M KOH | 73 mV | 72.1 mV·dec^−1^ | Sun et al., *ACS Materials Lett.*, **2024**, 6, 3977. |
| 756 | CoP@CNCs | 0.5 M H_2_SO_4_ | 144 mV | 58.3 mV·dec^−1^ | Li et al., *J. Mater. Chem. A*, **2019**, 7, 8443. |
| 757 | Co_x_P NPs/CC*^iR^* | 0.5 M H_2_SO_4_ | 90 mV | 67.9 mV·dec^−1^ | Tao et al., *Int. J. Hydrogen Energy*, **2019**, 44, 531. |
| 758 | Co–Fe–P/NF*^iR^* | 1.0 M KOH | − | 50.7 mV·dec^−1^ | Li et al., *Int. J. Hydrogen Energy*, **2021**, 46, 27883. |
| 759 | CoP/NF*^iR^* |  | − | 85.6 mV·dec^−1^ |  |
| 760 | Al-CoP/Ni_2_P@NF*^iR^* | 1.0 M KOH | 55 mV | 55.6 mV·dec^−1^ | Chen et al., *Fuel*, **2025**, 394, 135070. |
| 761 | CoP-NiCoP-Ti_3_C_2_T_x_*^iR^* | 1.0 M KOH | 46 mV | 75.3 mV·dec^−1^ | Xu et al., *J. Colloid Interface Sci.*, **2023**, 639, 223. |
| 762 | Ni_5%_CoP*^iR^* | 1.0 M KOH | 84 mV | 67 mV·dec^−1^ | Zhao et al., *J. Energy Chem.*, **2022**, 74, 203. |
| 763 | CoP@NC/NCNT*^iR^* | 0.5 M H_2_SO_4_ | 106 mV | 64.8 mV·dec^−1^ | Wu et al., *J. Colloid Interface Sci.*, **2023**, 629, 22. |
|  |  | 1.0 M KOH | 177 mV | 79 mV·dec^−1^ |  |
| 764 | NiMo_450_/CoP | 1.0 M KOH | 30 mV | 68 mV·dec^−1^ | Luo et al., *Fuel*, **2025**, 399, 135675. |
| 765 | V-CoP*^NiR^* | 1.0 M KOH | 46 mV | 51 mV·dec^−1^ | Zhang et al., *Adv. Energy Mater.*, **2021**, 11, 2101758. |
| 766 | CoP*^NiR^* |  | 67 mV | 53 mV·dec^−1^ |  |
| 767 | CoP/CC*^iR^* | 1.0 M KOH | − | 90 mV·dec^−1^ | Li et al., *Energy Fuels*, **2022**, 36, 13212. |
| 768 | Se–CoP/CC*^iR^* |  | − | 56 mV·dec^−1^ |  |
| 769 | V-Co_x_P@NC NPs*^iR^* | 1.0 M KOH | 106 mV | 93 mV·dec^−1^ | Guo et al., *Electrochim. Acta*, **2020**, 357, 136850. |
| 770 | Co_3_S_4_/CoP | 0.5 M H_2_SO_4_ | 86 mV | 45 mV·dec^−1^ | Wang et al., *Sci. Rep.*, **2017**, 7, 11891. |
| 771 | CoP-0.15M*^iR^* | 1.0 M KOH | 71 mV | 60.75 mV·dec^−1^ | Xu et al., *Inorg. Chem.*, **2021**, 60, 10781. |
| 772 | CoP@N,P–C*^iR^* | 0.5 M H_2_SO_4_ | 140 mV | 63 mV·dec^−1^ | Zhao et al., *ACS Appl. Mater. Interfaces*, **2021**, 13, 9820. |
|  |  | 1.0 M KOH | 197 mV | 40 mV·dec^−1^ |  |
| 773 | FeP/CoP@NPC*^iR^* | 0.5 M H_2_SO_4_ | 198 mV | 53.6 mV·dec^−1^ | Liu et al., *Dalton Trans.*, **2022**, 51, 12307. |
|  |  | 1.0 M PBS | 286 mV | 75.3 mV·dec^−1^ |  |
|  |  | 1.0 M KOH | 339 mV | 58.8 mV·dec^−1^ |  |
| 774 | CoP | 1.0 M KOH | 80 mV | 120 mV·dec^−1^ | Yang et al., *Appl. Surf. Sci.*, **2021**, 550, 149355. |
| 775 | N-CoP/NC | 0.5 M H_2_SO_4_ | 150 mV | 95 mV·dec^−1^ | Xu et al., *Nanoscale*, **2022**, 14, 17995. |
| 776 | Ni–CoxP NSs*^iR^* | 1.0 M KOH | 106 mV | 62.3 mV·dec^−1^ | Dai et al., *Ionics*, **2022**, 28, 2895. |
|  |  | 0.5 M H_2_SO_4_ | 172 mV | 68.3 mV·dec^−1^ |  |
| 777 | Mo-CoP/Co-N-C*^iR^* | 1.0 M KOH | 45 mV | 65 mV·dec^−1^ | Li et al., *J. Mater. Sci. Technol.*, **2023**, 166, 58. |
|  |  | 0.5 M H_2_SO_4_ | 41 mV | 64 mV·dec^−1^ |  |
|  |  | 1.0 M PBS | 98 mV | 101 mV·dec^−1^ |  |
| 778 | CoP–Ni_2_P@U-NCNTs/NF | 1.0 M KOH | − | 57.1 mV·dec^−1^ | Chen et al., *Rare Met.*, **2025**, 44, 4740. |
| 779 | MnO-CoP/NF*^iR^* | 1.0 M KOH | − | 55.3 mV·dec^−1^ | Dong et al., *Nano Lett.*, **2023**, 23, 9087. |
| 780 | Se-CoP*^iR^* | 1.0 M KOH | 123 mV | − | Zhang et al., *Angew. Chem. Int. Ed.*, **2025**, e202507040. |
| 781 | Asn-CoP*^iR^* | 0.5 M H_2_SO_4_ | − | 55.8 mV·dec^−1^ | Gao et al., *ACS Sustainable Chem. Eng.*, **2023**, 11, 13747. |
| 782 | 3% Bi/CoP*^NiR^* | 1.0 M KOH | 122 mV | 60.2 mV·dec^−1^ | Guo et al., *Chem. Commun.*, **2020**, 56, 7702. |
| 783 | Fe_2_P–CoP/CeO_2_-20*^iR^* | 1.0 M KOH | 45 mV | 47.6 mV·dec^−1^ | Ding et al., *Chem. Eng. J.*, **2023**, 451, 138550. |
| 784 | HI-CoP/CNT*^iR^* | 0.5 M H_2_SO_4_ | 73 mV | 54.6 mV·dec^−1^ | Adam et al., *ACS Appl. Mater. Interfaces*, **2018**, 10, 29407. |
|  |  | 1.0 M KOH | 125 mV | − |  |
| 785 | Ru/Co_x_P@NC*^iR^* | 0.5 M H_2_SO_4_ | 165 mV | 54.7 mV·dec^−1^ | Si et al., *ACS Sustainable Chem. Eng.*, **2019**, 7, 9737. |
| 786 | Ni-doped Co_2_P/CoP@N–C*^iR^* | 1.0 M KOH | 161 mV | 94 mV·dec^−1^ | Arunkumar et al., *ACS Appl. Energy Mater.*, **2022**, 5, 2975. |
| 787 | CoP–Ni_5_P_4_/NF*^iR^* | 1.0 M KOH | 118 mV | 86 mV·dec^−1^ | Jiang et al., *J. Mater. Chem. A*, **2023**, 11, 1801. |
| 788 | CoP NPs/CC*^NiR^* | 0.5 M H_2_SO_4_ | 48 mV | 70 mV·dec^−1^ | Li et al., *Int. J. Hydrogen Energy*, **2014**, 39, 16806. |
| 789 | CoFeP/CoP-400 | 1.0 M KOH | 78 mV | 55.5 mV·dec^−1^ | Tan et al., *Inorg. Chem.*, **2023**, 62, 9964. |
| 790 | CoP NWs/CC | 0.5 M H_2_SO_4_ | 118 mV | 73 mV·dec^−1^ | Lyu et al., *Mater. Chem. Front.*, **2018**, 2, 323. |
|  |  | 1.0 M PBS | 130 mV | 144 mV·dec^−1^ |  |
|  |  | 1.0 M KOH | 69 mV | 70 mV·dec^−1^ |  |
| 791 | CoP/NF*^iR^* | 1.0 M KOH | − | 83 mV·dec^−1^ | Chen et al., *Dalton Trans.*, **2023**, 52, 2262. |
|  |  | 0.5 M H_2_SO_4_ | − | 60 mV·dec^−1^ |  |
|  |  | 1.0 M PBS | − | 110 mV·dec^−1^ |  |
| 792 | CoP-Ni_2_P/CNT | 1.0 M KOH | 82 mV | 62.6 mV·dec^−1^ | Wu et al., *Catal. Today*, **2023**, 424, 113852. |
| 793 | CoP/CNT |  | 94 mV | 90.5 mV·dec^−1^ |  |
| 794 | FeP@CoP | 1.0 M KOH | 50 mV | 51.1 mV·dec^−1^ | Lyu et al., *Appl. Catal. B Environ.*, **2022**, 317, 121799. |
| 795 | Ni-CoP-2*^iR^* | 0.5 M H_2_SO_4_ | 110 mV | 65 mV·dec^−1^ | Sun et al., *Appl. Energy*, **2022**, 326, 119999. |
|  |  | 1.0 M KOH | 157 mV | 86 mV·dec^−1^ |  |
| 796 | V–CoP/VG/CC*^iR^* | 1.0 M KOH | 40 mV | 40 mV·dec^−1^ | Truong et al., *RSC Adv.*, **2020**, 10, 13016. |
| 797 | CoP/VG/CC*^iR^* |  | 93 mV | 55 mV·dec^−1^ |  |
| 798 | P-MoS_2_@CoP-2*^iR^* | 1.0 M KOH | 64 mV | 51.2 mV·dec^−1^ | Hu et al., *ChemSusChem*, **2021**, 14, 1565. |
|  |  | 0.5 M H_2_SO_4_ | 72 mV | 59.7 mV·dec^−1^ |  |
| 799 | CoP_x_/Co_9_S_8_-Cu NAs*^iR^* | 1.0 M KOH | 118 mV | 66.75 mV·dec^−1^ | Jiang et al., *J. Colloid Interface Sci.*, **2019**, 550, 10. |
| 800 | S-CoP/CC*^iR^* | 0.5 M H_2_SO_4_ | 96 mV | 56 mV·dec^−1^ | Zhang et al., *Fuel*, **2025**, 397, 135436. |
| 801 | CoP eletrodeposit*^iR^* | 0.5 M H_2_SO_4_ | 85 mV | − | Saadi et al., *J. Phys. Chem. C*, **2014**, 118, 29294. |
| 802 | CoP NB/C*^iR^* | 1.0 M KOH | 101 mV | 66.5 mV·dec^−1^ | Yan et al., *Mater. Today Energy*, **2019**, 12, 443. |
| 803 | CoP@C*^iR^* |  | 93 mV | 50.7 mV·dec^−1^ |  |
| 804 | Ultrafine CoP nanocrystals | 0.5 M H_2_SO_4_ | 125 mV | 68 mV·dec^−1^ | Li et al., *Langmuir*, **2020**, 36, 1916. |
|  |  | 1.0 M KOH | 119 mV | 77 mV·dec^−1^ |  |
| 805 | CoP/Ni_3_FeN*^iR^* | 1.0 M KOH | − | 53 mV·dec^−1^ | Liao et al., *Sci. China Mater.*, **2023**, 66, 3520. |
| 806 | HNDCM-Co/CoP*^iR^* | 0.5 M H_2_SO_4_ | 135 mV | 64 mV·dec^−1^ | Wang et al., *ACS Nano*, **2017**, 11, 4358. |
|  |  | 1.0 M KOH | 138 mV | 66 mV·dec^−1^ |  |
| 807 | CoP@PS/NCNT*^iR^* | 0.5 M H_2_SO_4_ | 80 mV | 53 mV·dec^−1^ | Li et al., *Adv. Energy Mater.*, **2018**, 8, 1702806. |
| 808 | CoP/FeCoP_x_*^NiR^* | 1.0 M KOH | 74 mV | 65.1 mV·dec^−1^ | Zhang et al., *Small Methods*, **2025**, 9, 2401139. |
| 809 | CoP/Co(OH)_2_@NF*^iR^* | 1.0 M KOH | 76 mV | 44.29 mV·dec^−1^ | Chu et al., *Inorg. Chem.*, **2023**, 62, 18189. |
| 810 | CoP@NF*^iR^* |  | 77 mV | 59.56 mV·dec^−1^ |  |
| 811 | Ni_5_Cu_3_/CoP*^iR^* | 1.0 M KOH | 66 mV | 72 mV·dec^−1^ | Luo et al., *Appl. Surf. Sci.*, **2025**, 697, 163025. |
| 812 | Ni2P–Ni5P4–CoP–C/NF*^iR^* | 1.0 M KOH | 58 mV | 58 mV·dec^−1^ | Ni et al., *Dalton Trans.*, **2024**, 53, 8732. |
| 813 | CoP*^iR^* | 1.0 M KOH | 118 mV | 58.6 mV·dec^−1^ | Wang et al., *Appl. Catal. B Environ. Energy*, **2024**, 352, 124002. |
| 814 | Fe-CoP*^iR^* |  | 76 mV | 51.1 mV·dec^−1^ |  |
| 815 | V_P_-Fe-CoP*^iR^* | 1.0 M KOH | 143 mV | 91.6 mV·dec^−1^ | Na et al., *Adv. Sustainable Syst.*, **2023**, 7, 2300130. |
| 816 | CoP@Ni_2_P*^iR^* | 1.0 M KOH | 101 mV | 79 mV·dec^−1^ | Jin et al., *RSC Adv.*, **2021**, 11, 22467. |
| 817 | CoP*^iR^* |  | 122 mV | 91 mV·dec^−1^ |  |
| 818 | 3D H-NP-CoP NFs/G IN*^iR^* | 0.5 M H_2_SO_4_ | 98.1 mV | 40.9 mV·dec^−1^ | Fan et al., *Sustain. Energy Fuels*, **2017**, 1, 2172. |
| 819 | V-CoP/MXene@NF*^iR^* | 1.0 M KOH | 78 mV | 56.14 mV·dec^−1^ | Wang et al., *Nano Res.*, **2025**, 18, 94907238. |
| 820 | CoP@C-2 | 1.0 M KOH | 127 mV | 78 mV·dec^−1^ | Shi et al., *CrystEngComm*, **2021**, 23, 1777. |
| 821 | CoP@NiCo LDH-100*^iR^* | 1.0 M KOH | 62 mV | 47 mV·dec^−1^ | Wang et al., *Nano Energy*, **2023**, 114, 108681. |
| 822 | CoP*^iR^* |  | 104 mV | 63 mV·dec^−1^ |  |
| 823 | O-CoP-40*^iR^* | 1.0 M KOH | 67 mV | 41.1 mV·dec^−1^ | Dong et al., *J. Colloid Interface Sci.*, **2022**, 622, 239. |
| 824 | Ni_2_P−CoP/NF*^iR^* | 1.0 M KOH | 96 mV | 57 mV·dec^−1^ | Wu et al., *ChemSusChem*, **2021**, 14, 5450. |
| 825 | Ru/B-CoP*^iR^* | 0.5 M H_2_SO_4_ | 39 mV | 51.2 mV·dec^−1^ | Wang et al., *Chem. Eng. J.*, **2023**, 452, 139175. |
|  |  | 1.0 M KOH | 52 mV | 63.6 mV·dec^−1^ |  |
|  |  | 1.0 M PBS | 87 mV | 89.8 mV·dec^−1^ |  |
| 826 | CoP@M-CA*^iR^* | 0.5 M H_2_SO_4_ | 107 mV | 61.9 mV·dec^−1^ | Wu et al., *Adv. Mater.*, **2021**, 33, 2007368. |
| 827 | CoP/Ni_3_P@NF*^iR^* | 1.0 M KOH | − | 75 mV·dec^−1^ | Zhang et al., *Adv. Funct. Mater.*, **2023**, 33, 2212381. |
| 828 | CoP/NF*^iR^* |  | − | 84 mV·dec^−1^ |  |
| 829 | Ru, Ni–CoP porous nanofibers*^iR^* | 1.0 M KOH | 45 mV | 53.9 mV·dec^−1^ | Song et al., *Appl. Catal. B Environ.*, **2021**, 298, 120488. |
| 830 | CoP*^iR^* | 1.0 M KOH | − | 74.6 mV·dec^−1^ | Lu et al., *Chin. J. Catal.*, **2025**, 69, 163. |
| 831 | Ni_2_P/CoP*^iR^* |  | − | 46.8 mV·dec^−1^ |  |
| 832 | Co_2_P/CoP/CF*^iR^* | 1.0 M KOH | 95 mV | 64.14 mV·dec^−1^ | Xiao et al., *Adv. Funct. Mater.*, **2025**, e07040. |
| 833 | Ni_0.5_Co_0.5_P-300*^iR^* | 1.0 M KOH | 87 mV | 58 mV·dec^−1^ | Li et al., *Electrochim. Acta*, **2017**, 249, 301. |
| 834 | 2D Ni_2_P/CoP*^iR^* | 1.0 M KOH | 46.1 mV | 53.5 mV·dec^−1^ | Li et al., *Nano Energy*, **2024**, 130, 110139. |
| 835 | CoP/MoO_2_/NC*^iR^* | 1.0 M KOH | 55 mV | 66 mV·dec^−1^ | Li et al., *Int. J. Hydrogen Energy*, **2024**, 80, 440. |
|  |  | 0.5 M H_2_SO_4_ | 50 mV | 55 mV·dec^−1^ |  |
|  |  | 1.0 M PBS | 99 mV | 92 mV·dec^−1^ |  |
| 836 | p-CoP/CP*^iR^* | 1.0 M KOH | 57 mV | 52 mV·dec^−1^ | Zeng et al., *Chem. Commun.*, **2018**, 54, 1465. |
|  |  | 0.5 M H_2_SO_4_ | 39 mV | 53 mV·dec^−1^ |  |
|  |  | 1.0 M PBS | 60 mV | 38 mV·dec^−1^ |  |
| 837 | CoP@BCMT*^NiR^* | 1.0 M KOH | 49 mV | 75 mV·dec^−1^ | Zhang et al., *Chem. Eng. J.*, **2022**, 440, 135884. |
| 838 | CoP*^NiR^* |  | 84 mV | 79 mV·dec^−1^ |  |
| 839 | CoP-Co_2_P@PC/PG NHs*^iR^* | 1.0 M KOH | 39 mV | 59 mV·dec^−1^ | Yang et al., *Small*, **2019**, 15, 1804546. |
| 840 | CoP@PC/PG*^iR^* |  | 46 mV | 65 mV·dec^−1^ |  |
| 841 | CoP-Co_2_P/C-CFs*^iR^* | 0.5 M H_2_SO_4_ | 84 mV | 70.7 mV·dec^−1^ | Lou et al., *J. Colloid Interface Sci.*, **2025**, 692, 137461. |
| 842 |  | 1.0 M KOH | 115 mV | 118.9 mV·dec^−1^ |  |
| 843 | Fe–CoP-1 | 1.0 M KOH | 85 mV | 68.7 mV·dec^−1^ | Zhou et al., *Int. J. Hydrogen Energy*, **2025**, 145, 139. |
| 844 | CoP_h_/NG*^iR^* | 1.0 M KOH | 83 mV | 57 mV·dec^−1^ | Yu et al., *Nanoscale*, **2016**, 8, 10902. |
| 845 | Co_2_P/CoP-NC-0.1*^iR^* | 1.0 M KOH | 91 mV | 86 mV·dec^−1^ | Li et al., *Sustain. Mater. Technol.*, **2022**, 32, e00421. |
|  |  | 0.5 M H_2_SO_4_ | 84 mV | 51 mV·dec^−1^ |  |
| 846 | CoP/C@Nb_2_CT_x_ | 0.1 M KOH | 50 mV | 49 mV·dec^−1^ | Murtaza et al., *Fuel*, **2025**, 394, 135095. |
| 847 | pCoMo-P/NF*^NiR^* | 1.0 M KOH | 49 mV | 43.31 mV·dec^−1^ | Han et al., *ACS Appl. Energy Mater.*, **2019**, 2, 6302. |
| 848 | CoP/BMHNC*^iR^* | 0.5 M H_2_SO_4_ | 91.3 mV | 42.6 mV·dec^−1^ | Wang et al., *ChemSusChem*, **2017**, 10, 1014. |
| 849 | 2D Fe-doped CoP arrays | 1.0 M KOH | − | 76 mV·dec^−1^ | Zhou et al., *Nano Energy*, **2017**, 41, 583. |
| 850 | Co_2_N/CoP@CC*^iR^* | 1.0 M KOH | 44 mV | 49 mV·dec^−1^ | Guo et al., *Chem. Eng. J.*, **2023**, 470, 144242. |
| 851 | CoP@CC*^iR^* |  | 103 mV | 72 mV·dec^−1^ |  |
| 852 | NiMoP/CoP/NF*^iR^* | 1.0 M KOH | 38 mV | 83 mV·dec^−1^ | Wang et al., *Phys. Chem. Chem. Phys.*, **2024**, 26, 11667. |
| 853 | Zn_0.1_-CoP*^iR^* | 1.0 M KOH | 98 mV | 44 mV·dec^−1^ | Wang et al., *J. Alloys Compd.*, **2023**, 934, 167828. |
| 854 | V_P_-V-CoP@Cu_3_P HNTAs/CF*^iR^* | 1.0 M KOH | 35 mV | 33.4 mV·dec^−1^ | Sun et al., *Adv. Funct. Mater.*, **2025**, 2505867. |
| 855 | V-CoP/CF*^iR^* |  | 80 mV | 70.1 mV·dec^−1^ |  |
| 856 | Ce_0.05_-doped CoP CNTs | 0.5 M H_2_SO_4_ | 146 mV | 78 mV·dec^−1^ | Chen et al., *Nanotechnology*, **2020**, 31, 125402. |
| 857 | Mn_0.6_Co_0.4_P–rGO*^iR^* | 1.0 M KOH | 54 mV | 63 mV·dec^−1^ | Xu et al., *Nanoscale Adv.*, **2019**,1, 177. |
| 858 | Co_2_P/CoP*^iR^* | 0.5 M H_2_SO_4_ | 144 mV | 58 mV·dec^−1^ | Tian et al., *J. Mater. Chem. A*, **2016**, 4, 13011. |
| 859 | Fe-CoP/Ni(OH)_2_*^iR^* | 1.0 M KOH | 91 mV | 48 mV·dec^−1^ | Yu et al., *Adv. Funct. Mater.*, **2021**, 31, 2101578. |
| 860 | V,Fe-CoP nanowire*^NiR^* | 1.0 M KOH | 62 mV | 64 mV·dec^−1^ | Gan et al., *Nanoscale*, **2025**,17, 12660. |
| 861 | NiCoP/CC | 0.5 M H_2_SO_4_ | 44 mV | 39 mV·dec^−1^ | Du et al., *ACS Catal.*, **2017**, 7, 4131. |
|  |  | 1.0 M KOH | 62 mV | 66.5 mV·dec^−1^ |  |
| 862 | CoP/BMHNC*^iR^* | 0.5 M H_2_SO_4_ | 95.8 mV | 33 mV·dec^−1^ | Yuan et al., *ACS Appl. Mater. Interfaces*, **2016**, 8, 20720. |
| 863 | NC-G-CoP/NF*^NiR^* | 1.0 M KOH | 68 mV | 69.15 mV·dec^−1^ | Li et al., *Int. J. Hydrogen Energy*, **2021**, 46, 18224. |
| 864 | CoP/NF*^NiR^* |  | 110 mV | 119.16 mV·dec^−1^ |  |
| 865 | Co/CoP/NC/CoF | 1.0 M KOH | 64 mV | 49.8 mV·dec^−1^ | Chen et al., *J. Colloid Interface Sci.*, **2023**, 645, 513. |
| 866 | CoP*^NiR^* | 1.0 M KOH | 208 mV | 88 mV·dec^−1^ | Meng et al., *ACS Appl. Mater. Interfaces*, **2019**, 11, 9023. |
| 867 | CoP/Co_9_S_8_*^NiR^* |  | 155 mV | 67 mV·dec^−1^ |  |
| 868 | CoP/CC*^NiR^* | 1.0 M KOH | − | 105 mV·dec^−1^ | Jiang et al., *ACS Appl. Energy Mater.*, **2024**, 7, 7895. |
| 869 | N-doped CoP ultrathin nanosheets*^iR^* | 1.0 M KOH | 50 mV | 55.4 mV·dec^−1^ | Wang et al., *ACS Sustainable Chem. Eng.*, **2020**, 8, 8949. |
| 870 | Hollow Co_x_P@NPS-C polyhedrons*^iR^* | 1.0 M KOH | 80 mV | 57.6 mV·dec^−1^ | Li et al., *Energy Technol.*, **2019**, 7, 1800757. |
| 871 | CoPv/CP*^NiR^* | 1.0 M KOH | 50 mV | 87 mV·dec^−1^ | Li et al., *Nano Lett.*, **2025**, 25, 10114. |
| 872 | Ce-CoP NWs/CC | 1.0 M KOH | 80 mV | 68.1 mV·dec^−1^ | Li et al., *J. Electroanal. Chem.*, **2022**, 922, 116748. |
| 873 | CoP@NiCoP*^NiR^* | 1.0 M KOH | 71 mV | 148 mV·dec^−1^ | Hu et al., *Small*, **2023**, 19, 2304132. |
| 874 | rGO/CoP-Rh-2.5*^iR^* | 0.5 M H_2_SO_4_ | 72 mV | 43 mV·dec^−1^ | Zheng et al., *J. Energy Chem.*, **2019**, 34, 72. |
| 875 | CoNiP/Co_x_P*^NiR^* | 1.0 M KOH | 36 mV | 70 mV·dec^−1^ | Liu et al., *Small*, **2021**, 17, 2007557. |
|  |  | 1.0 M PBS | 117 mV | − |  |
| 876 | Co_x_P*^NiR^* | 1.0 M KOH | 59 mV | 81 mV·dec^−1^ |  |
|  |  | 1.0 M PBS | 141 mV | − |  |
| 877 | CoP@NC-3/1*^iR^* | 1.0 M KOH | 143 mV | 74 mV·dec^−1^ | Li et al., *Appl. Surf. Sci.*, **2021**, 569, 151099. |
| 878 | V_0.2_Co_0.8_P/NF*^iR^* | 1.0 M KOH | 79 mV | 47.41 mV·dec^−1^ | Wang et al., *Eur. J. Inorg. Chem.*, **2023**, 26, e202300014. |
| 879 | MS-CoP_x_-350*^iR^* | 1.0 M KOH | 190 mV | 84 mV·dec^−1^ | Li et al., *Inorg. Chem. Front.*, **2023**, 10, 325. |
| 880 | Co-P@PC-750*^iR^* | 0.5 M H_2_SO_4_ | 72 mV | 49 mV·dec^−1^ | Wu et al., *Small*, **2020**, 16, 1900550. |
|  |  | 1.0 M PBS | 85 mV | − |  |
|  |  | 1.0 M KOH | 76 mV | − |  |
| 881 | Co–P–O2*^iR^* | 1.0 M KOH | 113 mV | 67 mV·dec^−1^ | Chen et al., *ACS Catal.*, **2023**, 13, 14802. |
| 882 | Co_x_P-NC-420 | 1.0 M KOH | 125 mV | 66 mV·dec^−1^ | Bo et al., *Electrochim. Acta*, **2022**, 403, 139643. |
| 883 | CoP-HS*^iR^* | 1.0 M KOH | 116 mV | 89 mV·dec^−1^ | Zhang et al., *Small*, **2022**, 18, 2103561. |
| 884 | C-Co_x_P*^iR^* | 0.5 M H_2_SO_4_ | 109 mV | 55 mV·dec^−1^ | Kim et al., *Small*, **2018**, 14, 1801284. |
|  |  | 1.0 M KOH | 121 mV | 62 mV·dec^−1^ |  |
| 885 | CoP_i_/PF-CNTs*^iR^* | 0.5 M H_2_SO_4_ | 105 mV | 32 mV·dec^−1^ | Ge et al., *Chin. J. Chem.*, **2021**, 39, 2113. |
| 886 | CoFeP/CF*^iR^* | 1.0 M KOH | 60.7 mV | 90.4 mV·dec^−1^ | Huang et al., *J. Colloid Interface Sci.*, **2020**, 569, 140. |

In the “Materials” column, the superscript “*iR*” indicates *iR*-corrected data, “*NiR*” denotes data collected without *iR*-compensation, and the absence of a superscript signifies that *iR*-correction details were not reported in the original literature. Based on the literature survey, approximately 68.4% of the reported HER studies employed *iR*-correction, whereas 10.0% explicitly reported uncorrected data and 21.6% did not specify whether *iR*-correction was applied. Despite these differences in data treatment, the overall trends in HER performance remain essentially unchanged.

Certain CoP-based materials exhibit overpotentials and Tafel slopes that drastically depart from the average values, which can likely be attributed to varied doping strategies or fabrication protocols. Based on our comprehensive data analysis, these rare deviations are considered statistical outliers and do not represent the bulk reaction characteristics of CoP as an HER catalyst.

**Supplementary Table 2.** Optimized crystallographic parameters of bulk cobalt phosphide (CoP) crystal (*Pnma*, orthorhombic, PDF#29-0497) compared to experimental and previous reported data.

| Lattice parameters | *a*/Å | *b*/Å | *c*/Å | *α*/° | *β*/° | *γ*/° |
| --- | --- | --- | --- | --- | --- | --- |
| Experimental  (*Acta Chem. Scand.*, **1962**, 16, 287.) | 5.077 | 3.281 | 5.587 | 90 | 90 | 90 |
| X. Cao, et al.  (*Phys. Chem. Chem. Phys.*, **2022**, 24, 4644.) | 5.032 | 3.242 | 5.480 | 90 | 90 | 90 |
| M. Huang, et al.  (*Dalton Trans.*, **2022**,51, 10420.) | 5.036 | 3.230 | 5.506 | 90 | 90 | 90 |
| D. Bahamon, et al.  (*RSC Adv.*, **2021**,11, 2947.) | 5.064 | 3.266 | 5.542 | 90 | 90 | 90 |
| Calculated  (this work, RPBE, VASP) | 5.089 | 3.288 | 5.572 | 90 | 90 | 90 |

As shown in Supplementary Table 2, the calculated crystallographic parameters of the optimized orthorhombic CoP agree well with the experimental and other reported results. In the bulk CoP unit cell, the coordination number is 6, namely, P is coordinated by 6 Co atoms and Co coordinated by 6 P atoms.

**Supplementary Table 3.** Calculated surface energies (γ, J/m^2^) for different exposed surfaces of CoP crystals. The “sym” and “asym” refer to the symmetrical and asymmetrical CoP slab models, respectively.

| Crystal planes | Lattice parameter | | | Surface areas (Å^2^) | Total energies of relaxed slabs  (*E*_slab_, eV) | Calculated  (this work, J/m^2^) |
| --- | --- | --- | --- | --- | --- | --- |
|  | *a*/Å | *b*/Å | *γ*/° |  |  |  |
| ^13, 14^(100)^*^ | 3.29 | 5.57 | 90.00 | 18.32 | -350.382 (asym)  -350.331 (sym) | 1.68  1.70 |
| ^15-17^(010)^*^ | 5.57 | 5.09 | 90.00 | 28.36 | -349.828 | 1.24 |
| ^18, 19^(001)^*^ | 5.09 | 3.29 | 90.00 | 16.73 | -351.688 | 1.21 |
| ^20-22^(011)^*^ | 5.09 | 6.47 | 90. 00 | 32.93 | -350.960 | 0.79 |
| ^20, 23^(111)^*^ | 6.06 | 6.47 | 106.01 | 37.68 | -348.699 (asym)  -349.172 (sym) | 1.17  1.07 |
| ^24, 25^(102)^*^ | 11.60 | 3.29 | 90.00 | 38.15 | -347.800 | 1.35 |
| ^26, 27^(201)^*^ | 12.25 | 3.29 | 90.00 | 40.28 | -347.170 | 1.40 |

^*^ Experimentally already observed in previous research

Noted that when cleaving different surfaces from the CoP cell, the surface dangling bonds will be produced by the fracture of Co–P bonds. The surface stability is strongly reversely correlated with the density of surface dangling bonds. To construct a stable facet needs to minimize the numbers of broken Co–P bonds in truncated surfaces for ensuring a lower energy of the exposed surface.

**Supplementary Table 4.** Calculated ∆*G*_*H_ (eV) for various pristine and HO*-covered surfaces of CoP crystals.

| *G* (eV) | Active sites of pristine surfaces | | | | Electrochemistry-induced HO* surfaces | | | |
| --- | --- | --- | --- | --- | --- | --- | --- | --- |
|  | (011) | (111) | (001) | (010) | (011) | (111) | (001) | (010) |
| ∆*G*_*H_ | 0.04 (P*)  0.28 (Co*) | –0.12 (P*)  0.15 (P*)  0.62 (Co*) | –0.42 (P*)  0.40 (Co*) | –0.16 (P*)  0.26 (Co*) | 0.12 (Co*) | 0.20 (P*)  0.30 (P*)  0.49 (Co*) | 0.19 (Co*)  0.25 (Co*) | 0.12 (Co*) |

^*^ P and Co in parentheses denote the active sites.

**Supplementary Table 5.** ICP-MS quantification of dissolved Co and P in the electrolytes following a 24 h static immersion of CoP (electrolyte-1) and after 24 h of HER electrocatalysis at 10 mA·cm^-2^ (electrolyte-2).

| Electrolytes | | Concentration of dissolved Co in the electrolyte (μg·L^-1^) | Concentration of dissolved P in the electrolyte (μg·L^-1^) |
| --- | --- | --- | --- |
| Electrolyte-1 | Acid | 1325.57 | 762.02 |
|  | Neutral | 409.72 | 451.65 |
|  | Alkaline | 977.29 | 526.63 |
| Electrolyte-2 | Acid | 120.13 | 98.60 |
|  | Neutral | 34.91 | 62.93 |
|  | Alkaline | 63.11 | 54.35 |

### References

(1) Hansen, H. A.; Rossmeisl, J.; Nørskov, J. K. Surface Pourbaix Diagrams and Oxygen Reduction Activity of Pt, Ag and Ni(111) Surfaces Studied by DFT. *Phys. Chem. Chem. Phys.* **2008**, *10* (25), 3722–3730. DOI: 10.1039/B803956A.

(2) Valdés, Á.; Qu, Z. W.; Kroes, G. J.; Rossmeisl, J.; Nørskov, J. K. Oxidation and Photo-Oxidation of Water on TiO2 Surface. *J. Phys. Chem. C* **2008**, *112* (26), 9872–9879. DOI: 10.1021/jp711929d.

(3) Guo, Z.; Yu, Y.; Li, C.; Campos dos Santos, E.; Wang, T.; Li, H.; Xu, J.; Liu, C.; Li, H. Deciphering Structure-Activity Relationship Towards CO_2_ Electroreduction over SnO_2_ by A Standard Research Paradigm. *Angew. Chem. Int. Ed.* **2024**, *63* (12), e202319913. DOI: 10.1002/anie.202319913.

(4) Wang, Y.; Zhang, D.; Sun, B.; Jia, X.; Zhang, L.; Cheng, H.; Fan, J.; Li, H. Divergent Activity Shifts of Tin-Based Catalysts for Electrochemical CO_2_ Reduction: pH-Dependent Behavior of Single-Atom Versus Polyatomic Structures. *Angew. Chem. Int. Ed.* **2025**, *64* (8), e202418228. DOI: 10.1002/anie.202418228.

(5) Guo, Z.; Wang, T.; Xu, J.; Cao, A.; Li, H. Surface Coverage and Reconstruction Analyses Bridge the Correlation Between Structure and Activity for Electrocatalysis. *Chem. Commun.* **2024**, *60* (97), 14346–14359. DOI: 10.1039/D4CC03875D.

(6) Liu, H.; Zhang, D.; Holmes, S. M.; D'Agostino, C.; Li, H. Origin of the Superior Oxygen Reduction Activity of Zirconium Nitride in Alkaline Media. *Chem. Sci.* **2023**, *14* (34), 9000–9009. DOI: 10.1039/D3SC01827J.

(7) Wang, Z.; Tang, M. T.; Cao, A.; Chan, K.; Nørskov, J. K. Insights into the Hydrogen Evolution Reaction on 2D Transition-Metal Dichalcogenides. *J. Phys. Chem. C* **2022**, *126* (11), 5151–5158. DOI: 10.1021/acs.jpcc.1c10436.

(8) Tang, M. T.; Liu, X.; Ji, Y.; Norskov, J. K.; Chan, K. Modeling Hydrogen Evolution Reaction Kinetics through Explicit Water–Metal Interfaces. *J. Phys. Chem. C* **2020**, *124* (51), 28083–28092. DOI: 10.1021/acs.jpcc.0c08310.

(9) Motagamwala, A. H.; Dumesic, J. A. Microkinetic Modeling: A Tool for Rational Catalyst Design. *Chem. Rev.* **2021**, *121* (2), 1049–1076. DOI: 10.1021/acs.chemrev.0c00394.

(10) Zhang, D.; Bao, Z.; Chu, Y.; Guo, Z.; Jia, X.; Jiang, Q.; Liu, H.; Liu, T.; Lu, T.; Lu, Y.; et al. Digital Catalysis Platform as a Gateway to Big Data and AI-powered Innovations in Catalysis. *Chem Catal.* **2026**, *6* (7). DOI: 10.1016/j.checat.2026.101775.

(11) Huang, Y.; Nielsen, R. J.; Goddard, W. A., III; Soriaga, M. P. The Reaction Mechanism with Free Energy Barriers for Electrochemical Dihydrogen Evolution on MoS_2_. *J. Am. Chem. Soc.* **2015**, *137* (20), 6692–6698. DOI: 10.1021/jacs.5b03329.

(12) Ye, S.; Liu, F.; She, F.; Chen, J.; Zhang, D.; Kumatani, A.; Shiku, H.; Wei, L.; Li, H. Hydrogen Binding Energy Is Insufficient for Describing Hydrogen Evolution on Single-Atom Catalysts. *Angew. Chem. Int. Ed.* **2025**, *64* (23), e202425402. DOI: 10.1002/anie.202425402.

(13) Li, X.; Jiang, L.; Liu, J.; Hua, Q.; Wang, E.; Xie, G. Size-dependent Catalytic Activity of Cobalt Phosphides for Hydrogen Evolution Reaction. *J. Energy Chem.* **2020**, *43*, 121–128. DOI: 10.1016/j.jechem.2019.08.018.

(14) Jiang, J.; Xu, G.; Li, Y.; Wang, C.; Zhang, L. Interfacial Engineering of Metal–organic Framework Derived Hierarchical CoP–Ni_5_P_4_ Nanosheet Arrays for Overall Water Splitting. *J. Mater. Chem. A* **2023**, *11* (4), 1801–1809. DOI: 10.1039/D2TA06866D.

(15) Alsabban, M. M.; Eswaran, M. K.; Peramaiah, K.; Wahyudi, W.; Yang, X.; Ramalingam, V.; Hedhili, M. N.; Miao, X.; Schwingenschlögl, U.; Li, L.-J.; et al. Unusual Activity of Rationally Designed Cobalt Phosphide/Oxide Heterostructure Composite for Hydrogen Production in Alkaline Medium. *ACS Nano* **2022**, *16* (3), 3906–3916. DOI: 10.1021/acsnano.1c09254.

(16) Zhang, K.; Jia, J.; Yang, E.; Qi, S.; Tian, H.; Chen, J.; Li, J.; Lou, Y.; Guo, Y. Work-function-induced Electron Rearrangement of In-plane FeP@CoP Heterojunction Enhances All pH Range and Alkaline Seawater Hydrogen Evolution Reaction. *Nano Energy* **2023**, *114*, 108601. DOI: 10.1016/j.nanoen.2023.108601.

(17) Xu, X.; Lu, Y.; Shi, J.; Hao, X.; Ma, Z.; Yang, K.; Zhang, T.; Li, C.; Zhang, D.; Huang, X.; et al. Corrosion-resistant Cobalt Phosphide Electrocatalysts for Salinity Tolerance Hydrogen Evolution. *Nat. Commun.* **2023**, *14* (1), 7708. DOI: 10.1038/s41467-023-43459-w.

(18) Chu, Y.; Wang, D.; Wang, J.; Zha, S.; Wu, M.; Liu, C.; Wang, W.; Mitsuzaki, N.; Chen, Z. Synergistic Interfacial Engineering of Heterostructured Cobalt Phosphide Spheres/Cobalt Hydroxide Nanosheets for Overall Water Splitting. *Inorg. Chem.* **2023**, *62* (44), 18189–18197. DOI: 10.1021/acs.inorgchem.3c02656.

(19) Wang, H.; Jiang, H.; Niu, Y.; Siddiqui, N. A.; Khan, A.; Pan, L.; Lin, J. Three-dimensional structured of V-doped CoP in situ grown on MXene as highly efficient bifunctional electrocatalyst for water splitting. *Nano Res.* **2025**, *18* (3), 94907238. DOI: 10.26599/NR.2025.94907238.

(20) Zhou, Q.; Shen, Z.; Zhu, C.; Li, J.; Ding, Z.; Wang, P.; Pan, F.; Zhang, Z.; Ma, H.; Wang, S.; et al. Nitrogen-Doped CoP Electrocatalysts for Coupled Hydrogen Evolution and Sulfur Generation with Low Energy Consumption. *Adv. Mater.* **2018**, *30* (27), 1800140. DOI: 10.1002/adma.201800140.

(21) Wang, X.; Na, Z.; Yin, D.; Wang, C.; Wu, Y.; Huang, G.; Wang, L. Phytic Acid-Assisted Formation of Hierarchical Porous CoP/C Nanoboxes for Enhanced Lithium Storage and Hydrogen Generation. *ACS Nano* **2018**, *12* (12), 12238–12246. DOI: 10.1021/acsnano.8b06039.

(22) Feng, J.-X.; Tong, S.-Y.; Tong, Y.-X.; Li, G.-R. Pt-like Hydrogen Evolution Electrocatalysis on PANI/CoP Hybrid Nanowires by Weakening the Shackles of Hydrogen Ions on the Surfaces of Catalysts. *J. Am. Chem. Soc.* **2018**, *140* (15), 5118–5126. DOI: 10.1021/jacs.7b12968.

(23) An, W.; Liu, Z.; Shen, T.; Du, Y.; Huang, C.; Duan, B.; Wang, C.; Huang, G.; Yan, Y.; Xu, S. Regulating d–p Hybridization in Mo-Doped CoP Nanoflowers with Superwetting Surfaces for Water Splitting at 3000 mA cm^–2^. *ACS Catal.* **2025**, *15* (12), 10358–10371. DOI: 10.1021/acscatal.4c07485.

(24) Dong, Y.; Deng, Z.; Xu, Z.; Liu, G.; Wang, X. Synergistic Tuning of CoO/CoP Heterojunction Nanowire Arrays as Efficient Bifunctional Catalysts for Alkaline Overall Water Splitting. *Small Methods* **2023**, *7* (7), 2300071. DOI: 10.1002/smtd.202300071.

(25) Popczun, E. J.; Read, C. G.; Roske, C. W.; Lewis, N. S.; Schaak, R. E. Highly Active Electrocatalysis of the Hydrogen Evolution Reaction by Cobalt Phosphide Nanoparticles. *Angew. Chem. Int. Ed.* **2014**, *53* (21), 5427–5430. DOI: 10.1002/anie.201402646.

(26) Su, L.; Cui, X.; He, T.; Zeng, L.; Tian, H.; Song, Y.; Qi, K.; Xia, B. Y. Surface reconstruction of cobalt phosphide nanosheets by electrochemical activation for enhanced hydrogen evolution in alkaline solution. *Chem. Sci.* **2019**, *10* (7), 2019–2024. DOI: 10.1039/C8SC04589E.

(27) Deng, M.; Yang, H.; Peng, L.; Zhang, L.; Tan, L.; He, G.; Shao, M.; Li, L.; Wei, Z. Insight into the Boosted Activity of TiO_2_–CoP Composites for Hydrogen Evolution Reaction: Accelerated Mass Transfer, Optimized Interfacial Water, and Promoted Intrinsic Activity. *J. Energy Chem.* **2022**, *74*, 111–120. DOI: 10.1016/j.jechem.2022.06.047.
